# Advantages of horizontal centrifugation of platelet‐rich fibrin in regenerative medicine and dentistry

**DOI:** 10.1111/prd.12625

**Published:** 2025-03-25

**Authors:** Nima Farshidfar, Karol Alí Apaza Alccayhuaman, Nathan E. Estrin, Paras Ahmad, Anton Sculean, Yufeng Zhang, Richard J. Miron

**Affiliations:** ^1^ Department of Periodontology University of Bern Bern Switzerland; ^2^ Department of Oral Biology University Clinic of Dentistry, Medical University of Vienna Vienna Austria; ^3^ Lake Erie College of Osteopathic Medicine School of Dental Medicine Bradenton Florida USA; ^4^ Department of Research Advanced PRF Education Venice Florida USA; ^5^ Department of Oral Implantology University of Wuhan Wuhan China

**Keywords:** Alb‐PRF, A‐PRF, Bio‐PRF, H‐PRF, leukocyte and platelet‐rich fibrin, L‐PRF

## Abstract

The aim of this comprehensive review was to evaluate comparative studies on horizontal and fixed‐angle centrifugation methods for preparing platelet‐rich fibrin (PRF). Furthermore, additional studies utilizing horizontal PRF (H‐PRF) were systematically investigated. This overview review article offers deeper insights into the advantages of H‐PRF when compared to fixed‐angle methods across a wide range of regenerative medical and dental applications. A comprehensive search was conducted in PubMed and Web of Science up to December 5, 2024. Grey literature was also searched via Google Scholar for additional relevant studies, and reference lists of eligible studies were screened for further potential inclusion. All in vitro, in vivo, and clinical studies that utilized horizontal or swing‐out centrifugation to prepare solid or liquid PRF, along with their subfractions such as the buffy coat, platelet‐poor plasma (PPP), or heated variants like albumin gel or albumin gel with liquid PRF (Alb‐PRF) as interventions, were included in this study. A total of 75 studies were included. Thirteen studies directly compared horizontal centrifugation to fixed‐angle centrifugation for producing PRF, while the remaining 62 studies were non‐comparative and focused on expanding the uses and clinical applications of H‐PRF. These studies spanned categories such as cell concentrations, fibrin matrix structure, growth factor release, antibacterial and anti‐inflammatory properties, and regenerative applications in bone, periodontal, cartilage, skin, hair, regenerative endodontics, corneal defect repair, wound healing, and soft tissue regeneration. Of the studies comparing horizontal to fixed‐angle centrifugation, 84.6% favored horizontal centrifugation, while 15.4% found no difference. None of the studies favored fixed‐angle centrifugation. Additionally, more optimized methods for concentrating liquid‐PRF (C‐PRF) using horizontal centrifugation and extending the resorption properties of PRF—ranging from 2 to 3 weeks to membranes lasting 4 months through an albumin denaturation process were—further discussed. Based on these findings, it remains logical to utilize H‐PRF in clinical practice owing to the greater superiority in results from the majority of studies. Nevertheless, further comparative clinical studies are needed to support these findings. While the current evidence is limited and further clinical trials are warranted, several studies have now indicated that horizontal centrifugation, compared to fixed‐angle, results in higher cell concentrations, more uniform cell distribution, and increased growth factor release. These advantages suggest that the use of H‐PRF may lead to enhanced clinical outcomes when the application of PRF is indicated. Since horizontal centrifugation can also lead to better cell separation, it should also be the preferred method for producing C‐PRF and Alb‐PRF for clinical applications.

## 1 INTRODUCTION

The utilization of autologous platelet concentrates (APCs) has gained tremendous attention over the past 25 years as an effective approach to stimulate tissue regeneration.^1^, ^2^, ^3^, ^4^, ^5^, ^6^, ^7^, ^8^ Platelet‐rich plasma (PRP) was a first‐generation APC that utilized anticoagulants and favored tissue regeneration with up to a six‐ to eightfold increase in platelet concentrations.^9^, ^10^, ^11^ Initial research indicated that PRP significantly enhanced the levels of several key growth factors, including platelet‐derived growth factor (PDGF), transforming growth factor β1 (TGF‐β1), and vascular endothelial growth factor (VEGF), compared to whole blood, thereby facilitating tissue wound healing.^12^, ^13^, ^14^ However, the use of anticoagulants in PRP preparation, coupled with its short‐term release of growth factors, limited its capacity for optimal tissue regeneration.^15^, ^16^, ^17^

To address these issues, platelet‐rich fibrin (PRF) was proposed as a second‐generation APC and developed to eliminate the use of anticoagulants.^18^, ^19^, ^20^, ^21^ Following centrifugation, PRF generates a solid autologous clot matrix that better mimics natural healing and facilitates a more controlled and gradual release of growth factors over time.^22^, ^23^, ^24^, ^25^ This autologous matrix, which contains platelets, leukocytes, and growth factors within its fibrin scaffold, creates a bioactive and biocompatible structure that enhances tissue repair and regeneration.^26^, ^27^, ^28^, ^29^ Additionally, a liquid form of PRF (i‐PRF) was introduced as the first liquid form of APC without necessitating the use of anticoagulants or other chemicals.^30^, ^31^, ^32^

Recently, it was demonstrated that the regenerative potential of PRF is highly influenced by the preparation technique employed by clinicians, particularly the centrifugation process.^33^, ^34^ Parameters such as relative centrifugal force (RCF), rotor angle, and centrifugation duration directly impact cell distribution, growth factor concentration, fibrin structure, and the mechanical stress exerted on cells during formation.^35^, ^36^, ^37^, ^38^ These variables can lead to substantial differences in PRF quality, potentially affecting its regenerative potential during clinical applications. More prominently, the orientation/angulation of centrifugation—whether fixed‐angle or horizontal—is particularly important, as it can affect biological characteristics and the distribution of critical cellular components within the final fibrin matrix.^39^

Although most scientific laboratories and medical centrifuges utilize horizontal swing‐out bucket rotors, which are more effective at separating layers based on density, PRF has been most commonly prepared using fixed‐angle centrifugation systems, typically designed for pelleting samples toward the bottom of centrifugation tubes.^33^ Fixed‐angle centrifugation systems hold tubes in an angulated position, creating uneven force distribution that pushes cells toward the outer tube walls, leading to non‐uniform layering and inconsistent cell separation. Studies indicate that the increased mechanical stress from this method can reduce cell viability and disrupt the integrity of the PRF matrix.^35^ Consequently, fixed‐angle centrifugation could limit the regenerative efficacy and potential of PRF.

Due to these limitations, horizontal centrifugation has been studied as a promising alternative in PRF therapy to offer enhanced biological properties by maintaining tubes in a horizontal position during rotation.^40^ Interestingly, this approach was initially utilized in the early development of PRP technology.^10^ This swing‐out bucket design allows for a balanced application of RCF and a more even distribution of cells throughout the various cell layers.^33^, ^39^ Additionally, it maximizes the difference between the RCF‐min and RCF‐max of the tubes, allowing faster cell separation towards their appropriate layers.^33^, ^39^ It has also previously been reported that horizontal centrifugation increases both platelet and leukocyte numbers and concentrations—up to fourfold increase in cells when compared to fixed‐angle centrifugation.^40^ Such findings imply that horizontal centrifugation not only preserves cellular integrity but may also improve the viability and potency of the PRF matrix. Table 1 also summarizes the key differences between fixed‐angle and horizontal centrifugation based on the available evidence.

**TABLE 1:** Key differences between fixed angle and horizontal centrifugation. Reprinted from Miron et al.^33^

Centrifugation method	Fixed‐angle centrifugation	Horizontal centrifugation
Optimized use	Fixed‐angle centrifugation is more useful for the pelleting of matter including cells to the bottom of tubes	Horizontal centrifugation is more useful for separating cells/matter based on density
Separation of cells	Cells accumulate against the back walls of PRF tubes	Cells are evenly distributed throughout the upper layers
Cell concentration	Capable of concentrating platelets. Not effective at separating leukocytes	Up to 4 times more cells and growth factor concentration; especially of leukocytes
Cell damage	Owing to higher g‐forces and fixed angles, cells are pushed toward the back walls and damaged. Clinicians can even visually see RBC accumulation along the back walls of tubes	Since cells are not driven toward the back walls, significantly lower chance of cell damage as a result of cells separating throughout the center of tubes
Protocols to create sticky bone	Most protocols combining liquid PRF and solid PRF require the removal of liquid PRF tubes within 3–5 min and thereafter to re‐spin the solid PRF tubes. Otherwise, a high chance of clotting may occur within the tubes	Both liquid and solid PRF tubes can be spun at the same time and same protocol. This is because cells are not driven toward the back walls of tubes that initiate clot formation
Protocols to create albumin gel	Not possible owing to the faster clotting times caused by fixed‐angle centrifugation	The protocol requires a horizontal centrifuge for optimization

Despite these promising findings, limited research exists comparing the effectiveness of horizontal centrifugation to fixed‐angle centrifugation. To address this gap, this comprehensive review aims to systematically evaluate existing comparative studies on horizontal and fixed‐angle centrifugation methods, investigating their effects on the biological properties of both solid and liquid PRF. Additionally, it encompasses all other non‐comparative studies utilizing horizontal centrifugation, offering deeper insights into its properties and potential applications in regenerative medicine and dentistry based on the available evidence.

## 2 SEARCH STRATEGY

An electronic search was conducted on December 5, 2024, utilizing databases including PubMed, Scopus, and Web of Science using the following keywords applied to the titles and abstracts of published studies: [((Platelet Rich Fibrin) OR (Platelet‐rich Fibrin) OR (PRF)) AND ((Horizontal) OR (Horizontal Centrifuge) OR (Horizontal Centrifugation) OR (Swing Out) OR (Swing‐out) OR (Swing‐out Rotor) OR (Swing‐out Bucket) OR (Swing‐out Rotor Centrifuge) OR (Swing‐out Bucket Centrifuge) OR (Swinging Bucket Rotor))] OR [(H‐PRF)] OR [((Platelet Rich Fibrin) OR (Platelet‐rich Fibrin) OR (PRF)) AND ((Albumin) OR (Albumin Gel) OR (Alb))] OR [(Alb‐PRF)]. Grey literature was also searched via Google Scholar for additional relevant studies, and reference lists of eligible studies were screened for further potential inclusions. The inclusion and exclusion criteria were established according to the Participants, Intervention, Comparison, and Outcome (PICO) framework. The inclusion criteria encompassed all in vitro, in vivo, and clinical studies that utilized horizontal or swing‐out centrifugation to prepare solid or liquid PRF, along with their subfractions, such as the buffy coat, platelet‐poor plasma (PPP), or its heated variants like albumin gel or albumin gel with liquid PRF (Alb‐PRF) as interventions. Studies utilizing other types of platelet concentrates, such as PRP, plasma rich in growth factors (PRGFs), concentrated growth factor (CGF), and others as intervention groups were excluded from our review. No specific criteria were set for comparison or outcome domains. Additionally, studies for which the PDF was unavailable, review articles, conference abstracts, protocol papers, technical notes, letters to the editor, and book chapters were excluded. Non‐English studies were also excluded due to the research team's language proficiency.

## 3 RESULTS

In total, the search strategies generated 987 articles from PubMed, Scopus, and Web of Science. After duplicate removal, 428 articles remained following abstract evaluation. A total of 359 papers were excluded due to a mismatch with our search criteria, and 69 articles were retained for the final full‐text review. Finally, 42 studies were included in our review from the aforementioned databases, as well as 33 additional studies from the grey literature and the reference list of relevant reviews. In sum, a total of 75 studies were included in this review. Based on the included papers, we divided the results section into two categories.

In the first category, we reviewed 13 comparative studies that compared horizontal to fixed‐angle centrifugation for producing PRF. Based on the findings, we divided this section into 10 clinically relevant subcategories to assess which method demonstrates superiority over the other. The subcategories were as follows: (1) PRF size and weight; (2) cell quantification, distribution, and fibrin structure; (3) growth factors and cytokine release; (4) antibacterial properties; (5) cellular functions; (6) tissue reaction; (7) bone regeneration; (8) sinus floor augmentation; (9) periodontal regeneration; and (10) overall comparative results.

In the second category, we reviewed the remaining 62 non‐comparative studies that utilized horizontal centrifugation to generate novel data that further extend its uses and clinical applications. This section was also divided into 23 clinically relevant subcategories: (1) centrifugation protocols; (2) concentrated platelet‐rich fibrin (C‐PRF); (3) albumin gel with liquid platelet‐rich fibrin (Alb‐PRF); (4) growth factors and cytokines release; (5) anti‐inflammatory properties; (6) antibacterial properties; (7) wound healing and soft tissue regeneration; (8) oral cancer treatment; (9) cartilage regeneration; (10) orthopedic treatment; (11) bone regeneration; (12) sinus floor augmentation; (13) dental implant; (14) Treatment of the palatogingival groove; (15) treatment of external root resorption and radicular cysts; (16) bone healing after tooth hemisection; (17) tooth autotransplantation; (18) regenerative endodontic treatment; (19) drug delivery; (20) corneal regeneration; (21) skin and hair regeneration; (22) myringoplasty; and (23) further optimization of H‐PRF.

### 3.1 Section 1: Studies comparing horizontal centrifugation versus fixed‐angle centrifugation

To date, 13 studies have been identified that compared horizontal and fixed‐angle centrifugation methods in preparing PRF matrices. Based on these studies, we have categorized these findings into the following sections: (1) PRF size and weight; (2) cell quantification, distribution, and fibrin structure; (3) growth factors and cytokines release; (4) antibacterial properties; (5) cellular functions; (6) tissue reaction; (7) bone regeneration; (8) sinus floor augmentation; (9) periodontal regeneration; and (10) overall comparative results, to assess which method demonstrates superiority across various biological properties. Table 2 provides a summary of studies comparing horizontal centrifugation of PRF versus fixed‐angle.

**TABLE 2:** Summary of the studies comparing fixed‐angle centrifugation versus horizontal centrifugation.

Authors (Year)	Area of focus	Aim of study	Type of study	Blood source	Fixed‐angle centrifugation group(s): preparation method and rotor angle (centrifuge device)	Horizontal centrifugation group(s): preparation method (centrifuge device)	Main measured variable(s): assessment method(s)	Main outcome(s)
Miron et al.^40^ (2019)	Cell quantification, distribution, and fibrin structure	To compare different commercially available centrifuges and their respective protocols utilizing a novel method to quantify cells	In vitro study	Human	(1) Solid PRF: 700 *g* (2700 rpm) × 12 min NR (IntraSpin Centrifuge, USA) (2) Liquid PRF: 700 *g* (2700 rpm) × 3 min NR (IntraSpin Centrifuge, USA) (3) Solid PRF: 200 *g* (1300 rpm) × 8 min NR (Duo Centrifuge, France) (4) Liquid PRF: 60 *g* (800 rpm) × 3 min NR (Duo Centrifuge, France)	(5) Solid PRF: 700 *g* × 8 min (5702 Eppendorf Centrifuge, Germany) (6) Liquid PRF: 200 *g* × 3 min (5702 Eppendorf Centrifuge, Germany)	Sequential quantification of cells: Hematology analyzer	PRF produced via horizontal centrifugation showed greater accumulation and concentration of platelets and leukocytes compared to fixed‐angle centrifugation
Viswa et al. (2019)	Cell quantification, distribution, and fibrin structure	To evaluate fibrinogen content and effectiveness in bone‐added osteotome sinus floor elevation of L‐PRF generated by the standard protocol (2700 RPM for 12 min) versus an RCF‐adjusted protocol (400 g) across two centrifuges with swing‐out rotors. The outcomes were also compared to a standard centrifuge configured to generate L‐PRF	In vitro and clinical study	Human	(1) Solid PRF: NR (Duo Quattro Centrifuge, France)	(2) Solid PRF: 2700 rpm × 12 min (Remi 8C Centrifuge, India) (3) Solid PRF: 400 *g* (1700 rpm) × 12 min (Remi 8C Centrifuge, India) (4) Solid PRF: 2700 rpm × 12 min (Remi C854 Centrifuge, India) (5) Solid PRF: 400 *g* (1400 rpm) × 14 min (Remi C854 Centrifuge, India)	Fibrinogen content: Fibrinogen assay kit Tissue healing and regeneration: Radiography	L‐PRF prepared using the horizontal centrifuge with an RCF‐adjusted protocol demonstrated fibrinogen content comparable to that produced by the Duo Quattro centrifuge. Additionally, L‐PRF generated with the horizontal centrifuge using the RCF‐adjusted protocol resulted in the highest postoperative bone height gain and bone fill, exceeding the outcomes achieved with the Duo Quattro centrifuge
	Sinus floor augmentation							
Aizawa et al.^41^ (2020)	Cell quantification, distribution, and fibrin structure	To quantitatively and qualitatively re‐evaluate the distribution of platelets in four different PRF matrices using a non‐invasive NIR imaging method	In vitro study	Human	(1) L‐PRF: 400 *g* × 12 min 33° of rotor angle (Hettich EBA 200 Centrifuge, Germany) (2) A‐PRF: 200 *g* × 14 min 41.3° of rotor angle (Duo Quattro Centrifuge, France) (3) CGF: 692 *g* × 2 min 547 *g* × 4 min 592 *g* × 4 min 885 *g* × 3 min 33° of rotor angle (Medifuge Centrifuge, Italy)	(4) H‐PRF: 700 *g* × 8 min (5702 Eppendorf Centrifuge, Germany)	Distribution of cells: Immunofluorescence staining and near‐infrared imaging Histology	In L‐PRF and CGF, platelets were primarily located on and beneath the distal surface, whereas in H‐PRF and A‐PRF, platelet distribution was widespread and uniform
Lourenço et al.^37^ (2020)	Cell quantification, distribution, and fibrin structure	To evaluate the impact of rotor angle (fixed‐angle and horizontal) and time of storage after centrifugation (20 min, 40 min, and 60 min) on the in vitro biological properties of PRF membranes	In vitro study	Human	(1) L‐PRF: 708 *g* × 12 min 41.3° of rotor angle (Duo Centrifuge, France)	(2) H‐PRF: 708 *g* × 12 min (B‐40 Centrifuge, Brazil)	Quantification of cells: Hematology analyzer 2 Structural analysis: Scanning electron microscopy 3 Quantification of growth factor and cytokine release: ELISA	Both centrifugation angles produced PRF membranes with similar structure and cellularity, but horizontal centrifugation induced a higher growth factor release. Storage time post‐centrifugation did not affect cell content or growth factor release
	Growth factors and cytokines release							
Sato et al.^42^ (2020)	Cell quantification, distribution, and fibrin structure	To visualize and evaluate the differences in the distribution of activated platelets using commonly utilized PRF preparation protocols	In vitro study	Human	(1) L‐PRF: 700 *g* (2700 rpm) × 12 min NR (Hettich EBA 200 Centrifuge, Germany) (2) A‐PRF: 200 *g* (1300 rpm) × 14 min NR (Duo Quattro Centrifuge, France) (3) CGF: 685 *g* × 2 min 540 *g* × 4 min 685 *g* × 4 min 845 *g* × 3 min NR (Medifuge Centrifuge, Italy)	(4) H‐PRF: 700 *g* (2200 rpm) × 8 min (5702 Eppendorf Centrifuge, Germany)	Distribution of cells: Immunofluorescence staining and near‐infrared imaging	Qualitative morphological analysis showed that intact platelets were widely and evenly distributed in H‐PRF and A‐PRF but were concentrated along the distal tube surface in L‐PRF and CGF. Activated platelets followed a similar distribution pattern to whole platelets in A‐PRF, L‐PRF, and CGF, while in H‐PRF, they were primarily located in the buffy coat region
Feng et al.^43^ (2020)	PRF Size and weight	To compare the antimicrobial effects of PRFs against *Staphylococcus aureus* and *Escherichia coli* in vitro and to determine whether the antibacterial effects correlated with the number of immune cells. Blood samples were obtained from eight healthy donors to prepare L‐PRF and H‐PRF	In vitro study	Human	(1) L‐PRF: 700 *g* × 12 min NR (Chixin Biotech Centrifuge, China)	(2) H‐PRF: 700 *g* × 8 min (NR)	PRF size and weight Quantification of cells: Flow cytometry 3 Antibacterial activity: Inhibition ring test Plate‐counting test	No significant differences in size or weight were found between the L‐PRF and H‐PRF groups. However, the H‐PRF group contained a higher leukocyte count than the L‐PRF group. Both PRF groups showed substantial antimicrobial activity against *S. aureus* and *E. coli*, but H‐PRF exhibited a significantly stronger antibacterial effect compared to L‐PRF
	Cell quantification, distribution, and fibrin structure							
	Antibacterial properties							
Fujioka‐Kobayashi et al.^35^ (2021)	Cell quantification, distribution, and fibrin structure	To visually and histologically characterize the cell separation manner and blood cell localization on the whole PRF clots prepared by two different centrifugation systems, fixed‐angle and horizontal centrifugation	In vitro study	Human	(1) L‐PRF: 400 *g* (2700 rpm) × 12 min NR (IntraSpin Centrifuge, USA)	(2) H‐PRF: 700 *g* × 8 min (5702 Eppendorf Centrifuge, Germany)	Macroscopic parameter: Visual observation Structural analysis: Scanning electron microscopy Distribution of cells: Histology	Horizontal centrifugation led to more uniform blood cell separation in PRF clots compared to the cell accumulation along the distal tube surfaces observed with fixed‐angle centrifugation
Shirakata et al.^44^ (2021)	Periodontal regeneration	To histologically compare the effects of PRF produced using different protocols on periodontal wound healing/regeneration in periodontal defects in dogs	In vivo study	Animal (Beagle Dog)	(1) PRF: 700 *g* (2700 rpm) × 12 min NR (IntraSpin Centrifuge, USA)	(2) H‐PRF: 700 *g* × 8 min (Bio‐PRF Centrifuge, USA) (3) Alb‐PRF: 700 *g* × 8 min + PPP (heating for 10 min at 75°C) (Bio‐PRF Centrifuge, USA)	Tissue healing and regeneration: Histology Histomorphometry	In PRF‐treated intrabony defects, new bone and cementum formation occurred to different extents regardless of the PRF production protocols used. Notably, in two‐wall intrabony defects, new bone formation in H‐PRF‐treated sites extended from the host bone toward the coronal area of the defects more than in the OFD, Fixed‐angle‐PRF, and Alb‐PRF groups, with the H‐PRF group showing the highest amount of newly formed cementum
Gheno et al.^36^ (2021)	Cell quantification, distribution, and fibrin structure	To evaluate the inflammatory reaction, biocompatibility, and extended degradation properties of a new autologous Alb PRF membrane in comparison to commonly utilized standard PRF after nude mice implantation	In vitro and in vivo study	Human	(1) L‐PRF: 700 *g* (2700 rpm) × 12 min NR (IntraSpin Centrifuge, USA)	(2) H‐PRF: 700 *g* × 8 min (5702 Eppendorf Centrifuge, Germany) (3) Alb‐PRF: 700 *g* × 8 min + PPP (heating for 10 min at 75°C) (Bio‐PRF Centrifuge, USA)	Tissue reaction: Histology PRF Degradation: Surface area measurement Histology Structural analysis: Scanning electron microscopy Quantification of growth factor release: ELISA	All groups showed excellent biocompatibility. L‐PRF and H‐PRF membranes were substantially or completely resorbed by 21 days, while the Alb‐PRF membrane remained volume‐stable. Scanning electron microscopy revealed L‐PRF has a dense fibrin network entrapping cells, H‐PRF has similar but higher cell entrapment, and Alb‐PRF has a dense protein layer coating fibrin fibers and encasing cells and platelets. FGF‐2 release was similar across all groups, but VEGF and PDGF release in H‐PRF was nearly double that of L‐PRF and Alb‐PRF
	growth factors and cytokines release							
	Tissue reaction							
Al‐Maawi et al.^45^ (2022)	PRF size and weight	To evaluate the hypothesis whether PRF components and bioactivity are modified using a horizontal centrifuge compared with a fixed angle rotor when applying the same RCF and centrifugation time in three different investigated centrifuges	In vitro study	Human	(1) Solid PRF: 710 *g* (2400 rpm) × 8 min NR (Process for PRF, France) (2) Liquid PRF: 44 *g* (600 rpm) × 8 min NR (Process for PRF, France)	(3) Solid PRF: 710 *g* (2240 rpm) × 8 min (Bio‐PRF Centrifuge, USA) (4) Liquid PRF: 51 *g* (600 rpm) × 8 min (Bio‐PRF Centrifuge, USA) (5) Solid PRF: 710 *g* (2254 rpm) × 8 min (Hettich EBA, Germany) (6) Liquid PRF: 51 *g* (600 rpm) × 8 min (Hettich EBA, Germany)	PRF size Quantification of cells: Hematology analyzer 3 Distribution of cells: Histology 4 Quantification of growth factor release: ELISA 5 Cell adhesion: DAPI staining 6 Cell viability: MTS assay	Solid PRF clots were significantly larger in the fixed rotor centrifuge group than in the two horizontal rotor groups, with no differences in cellular distribution. Cell density in liquid PRF and growth factor release over 7 days showed no significant differences. Osteoblast and fibroblast viability with PRF was similar across all groups
	Cell quantification, distribution, and fibrin structure							
	Growth factors and cytokines release							
	Cellular functions							
Ferreira Sávio et al.^46^ (2023)	Cell quantification, distribution, and fibrin structure	To evaluate bone neoformation in critical‐size defects treated with PRF membranes produced by fixed‐angle and horizontal centrifugation protocols	In vitro and in vivo study	Animal (Wistar Rat)	(1) L‐PRF: 700 *g* (2700 rpm) × 12 min 33° of rotor angle (IntraSpin Centrifuge, USA) (2) A‐PRF: 200 *g* (1500 rpm) × 14 min 33° of rotor angle (IntraSpin Centrifuge, USA)	(3) H‐PRF: 700 *g* (2000 rpm) × 8 min (5702 Eppendorf Centrifuge, Germany)	Sequential quantification of cells: Hematology analyzer Tissue healing and regeneration: Micro‐computed tomography Confocal scanning laser microscopy Histology Histomorphometry	The A‐PRF and H‐PRF protocols were more effective at concentrating platelets and achieving a more even distribution throughout the PRF matrix than the original L‐PRF protocol. In terms of leukocytes, H‐PRF protocol resulted in a twofold increase in cell numbers and a more uniform distribution across the PRF layers compared to fixed‐angle centrifugation protocols. The H‐PRF group demonstrated higher values for bone volume, number of trabeculae, newly formed bone area, and increased precipitation of alizarin compared to both the A‐PRF and L‐PRF groups
	Bone regeneration							
Chiara et al.^47^ (2024)	PRF size and weight	To evaluate the feasibility of A‐PRF+ production in the field and the correlation between hematological parameters, macroscopic and microscopic features in equine A‐PRF+, obtained with fixed‐angle and horizontal centrifuges	In vitro study	Animal (Horse)	(1) A‐PRF+: 1300 rpm × 8 min NR (Duo Centrifuge, France)	(2) H‐PRF: 1300 rpm × 8 min (5702 Eppendorf Centrifuge, Germany)	PRF size and weight Quantification of cells: Hematology analyzer Distribution of cells: Histology	Clot and membrane weights, lengths, widths, and heights showed no differences between horizontal and fixed‐angle centrifugation. Hematological parameters did not significantly correlate with clot or membrane size. Both protocols produced membranes with a loose fibrin structure and evenly distributed cells
	Cell quantification, distribution, and fibrin structure							
Lourenço et al.^48^ (2024)	Cell quantification, distribution, and fibrin structure	To evaluate Alb‐PRF's capacity for cytokine and growth factor release, along with its effects on the proliferation, differentiation, and mineralization of human osteoblasts in vitro	In vitro study	Human	(1) L‐PRF: 700 *g* (2700 rpm) × 12 min NR (Duo Centrifuge, France)	(2) Alb‐PRF: 700 *g* × 8 min + PPP (heating for 10 min at 75°C) (Bio‐PRF Centrifuge, USA)	Distribution of cells and structural analysis: Histology Scanning electron microscopy Fluorescent microscopy 2 Quantification of growth factor and cytokine release: Multiplex assay 3 Cell viability: XTT assay 4 Cell proliferation: Crystal violet test 5 Mineralization: ALP activity test Alizarin red staining 6 Gene expression: RT‐PCR	Structural analysis revealed that Alb‐PRF is a biphasic, highly cellularized material that releases lower levels of inflammatory cytokines while producing higher concentrations of PDGF and VEGF compared to L‐PRF. Alb‐PRF also demonstrated greater early ALP activity, enhanced in vitro mineralization, and a significant increase in the OPG/RANKL mRNA ratio
	Growth factors and cytokines release							
	Cellular functions							

Abbreviations: Alb‐PRF, albumin gel and liquid platelet‐rich fibrin; ALP, alkaline phosphatase; A‐PRF, advanced platelet‐rich fibrin; CGF, concentrated growth factors; EGF, vascular endothelial growth factor; ELISA, enzyme‐linked immunosorbent assay; FGF, fibroblast growth factor 2; H‐PRF, horizontal platelet‐rich fibrin; L‐PRF, leukocyte platelet‐rich fibrin; mRNA, messenger ribonucleic acid; NIR, near infra‐red; NR, Not reported; OPG, osteoprotegerin; PDGF, platelet‐derived growth factor; PPP, platelet‐poor plasma; PRF, platelet‐rich fibrin; RANKL, receptor activator of nuclear factor kappa‐B ligand; RCF, relative centrifugal force; RPM, revolution per minute; RT‐PCR, reverse transcription polymerase chain reaction.

#### 3.1.1 PRF size and weight

Three studies compared the size and weight of solid PRF prepared using horizontal and fixed‐angle centrifugation.^43^, ^45^, ^47^ While two studies found no significant difference in the PRF produced by either method,^43^, ^47^ one study reported that solid PRF clots were significantly larger in the fixed‐angle centrifuge group.^45^ Overall, it has previously been reported that the size of the membrane has more to do with a higher RCF value/time and little relevance on cellular content;^33^ in fact, the lower speeds and times have typically been shown to lead to higher cell contents, as discussed below.^49^

#### 3.1.2 Cell quantification, distribution, and fibrin structure

**FIGURE 1:**
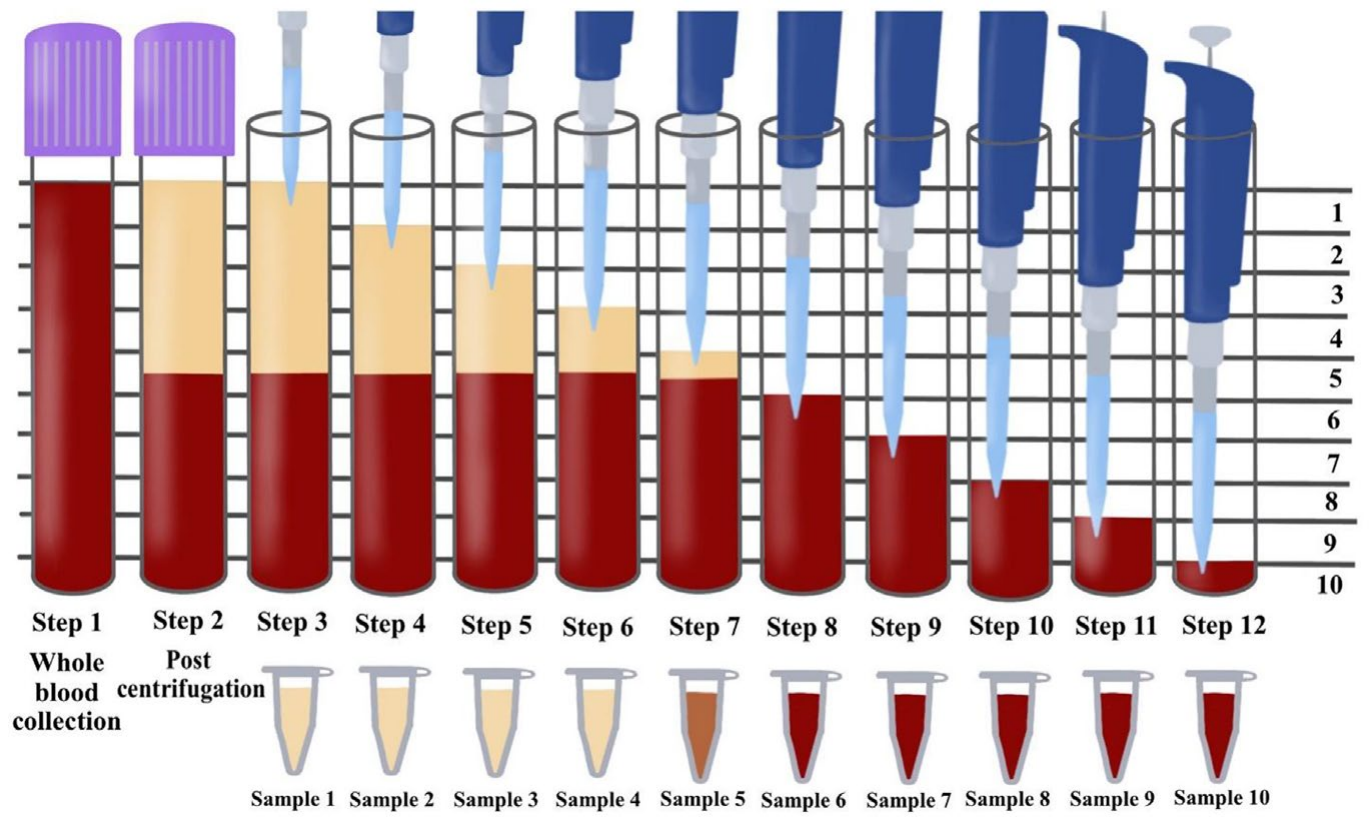
Illustration demonstrating the proposed novel method to quantify cell types following centrifugation of PRF. Currently, one of the limitations is that whole blood is compared to the total plasma concentration following centrifugation. This, however, does not give a proper representation regarding the location of cells following centrifugation. By utilizing the proposed technique in this study by sequentially pipetting 1 mL of volume from the top layer downward, it is then possible to send each of the 10 samples for CBC analysis and accurately determine the precise location of each cell type following centrifugation at various protocols. Notice that one layer (in this case layer 5) will contain some yellow plasma and red blood cells. This is typically the location of the buffy coat where a higher concentration of platelets is typically located. Reprinted with permission from Miron et al.^40^

**FIGURE 2:**
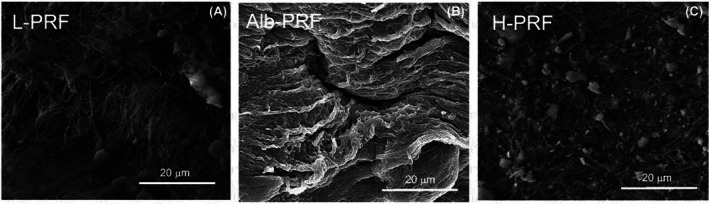
Scanning electron micrographs of the (A) L‐PRF, (B) Alb‐PRF, and (C) H‐PRF membranes obtained with a SEM (JEOL JSM‐6490 LV, JEOL, Japan) at 15 kV. Reprinted with permission from Gheno et al.^36^

Twelve of the 13 studies that compared horizontal centrifugation with fixed‐angle centrifugation investigated either cell quantification and distribution or its fibrin matrix structure.^35^, ^36^, ^37^, ^40^, ^41^, ^42^, ^43^, ^45^, ^46^, ^47^, ^48^, ^50^ In 2019, Miron et al. were the first to study cell quantification and distribution between fixed‐angle and horizontal PRF (H‐PRF) protocols.^40^ In this study, 1 mm blood layers from both fixed‐angle and horizontal centrifugations were sequentially pipetted from the top layer downward until the entire 10 mL was collected in samples (Figure 1). These samples were then analyzed via cell blood count (CBC) to accurately quantify cell numbers within each distinct blood layer.^40^ The results demonstrated that leukocyte‐ and platelet‐rich fibrin (L‐PRF) protocols (2700 rpm × 12 min) produced a clot with most platelets and leukocytes concentrated within the buffy coat, with minimal cell presence in the upper 4 mL of L‐PRF. In contrast, slower centrifugation speeds for less time using advanced platelet‐rich fibrin (A‐PRF) protocols (1300 rpm × 8 min) yielded a more even distribution of platelets throughout the PRF matrix, however, with a lack of leukocytes. Horizontal centrifugation significantly increased both platelet and leukocyte numbers and concentrations (up to fourfold higher for both solid and liquid PRF) compared to fixed‐angle centrifugation.^40^ Supporting these findings, Ferreira Sávio et al. conducted sequential cell quantification for L‐PRF (2700 rpm × 12 min), A‐PRF (1300 rpm × 8 min), and H‐PRF (700 *g* × 8 min) protocols, showing that low‐speed (A‐PRF) and horizontal (H‐PRF) centrifugation protocols more effectively concentrated platelets and distributed them evenly across the PRF matrices compared to the original L‐PRF protocol.^46^ For leukocytes, horizontal centrifugation resulted in a twofold increase in cell numbers and a more uniform distribution between PRF layers compared to fixed‐angle centrifugations.^46^ Feng et al. also demonstrated that the horizontal centrifugation protocol (700 *g* × 8 min) can produce an H‐PRF matrix containing more leukocytes than the L‐PRF matrix generated using fixed‐angle centrifugation (700 *g* × 12 min).^43^ Similarly, Gheno et al., using scanning electron microscopy (SEM), showed that H‐PRF membranes have a similarly dense fibrin network as L‐PRF membranes but contain more entrapped cells than L‐PRF membranes. Alb‐PRF membranes, which were also prepared by horizontal centrifugation, showed a dense surface layer of denatured protein, fully coating the fibrin fibers and encasing trapped cells and platelets (Figure 2).^36^

**FIGURE 3:**
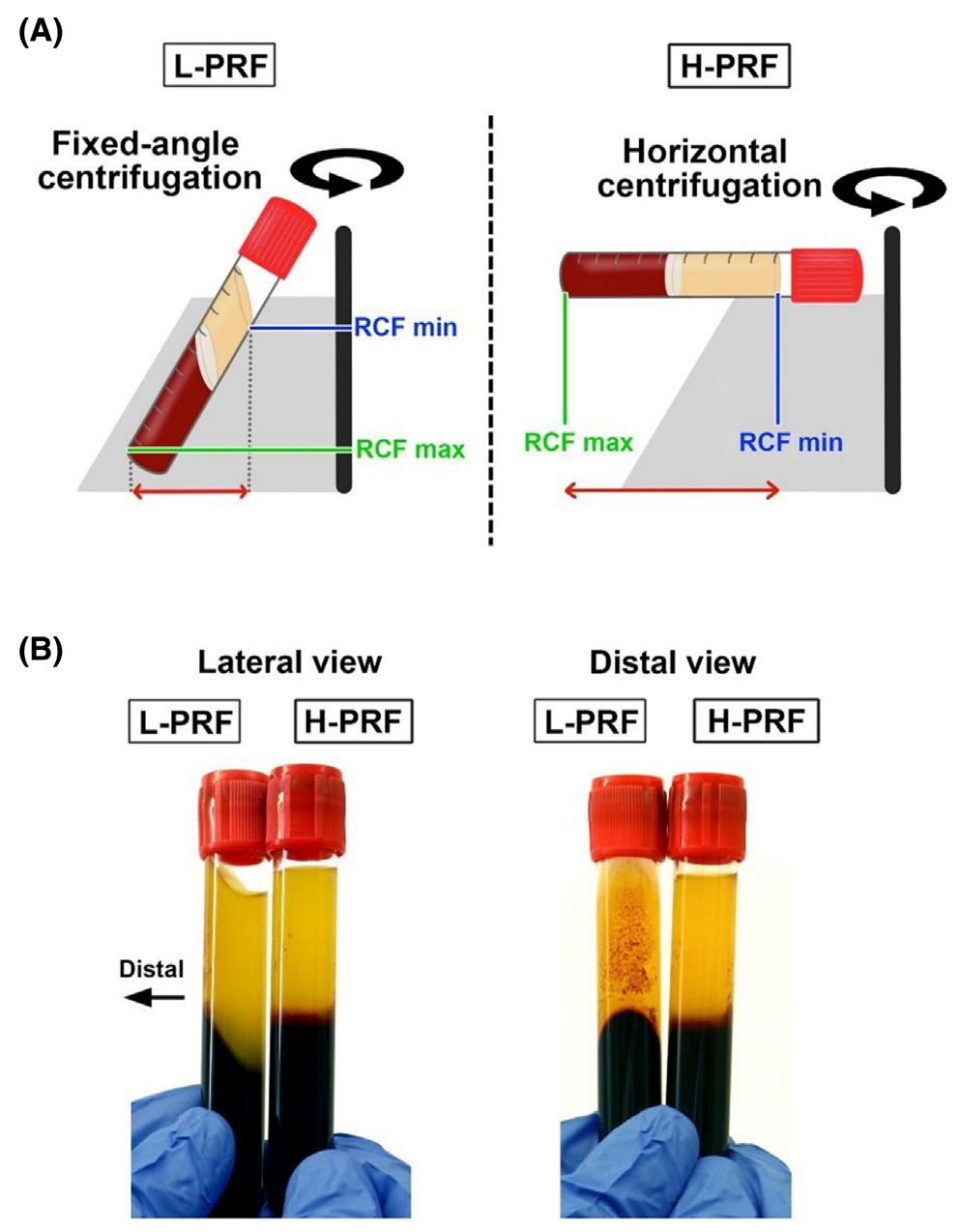
(A) Illustration of PRF produced via fixed‐angle (L‐PRF) and horizontal centrifugation (H‐PRF). (B) Visual representation of layer separation following either L‐PRF or H‐PRF protocols. L‐PRF clots are prepared with a sloped shape and multiple red dots are often observed on the distal surface of PRF tubes while H‐PRF was prepared with a horizontal layer separation between the upper plasma and lower red corpuscle layer. Reprinted with permission from Fujioka‐Kobayashi et al.^35^

**FIGURE 4:**
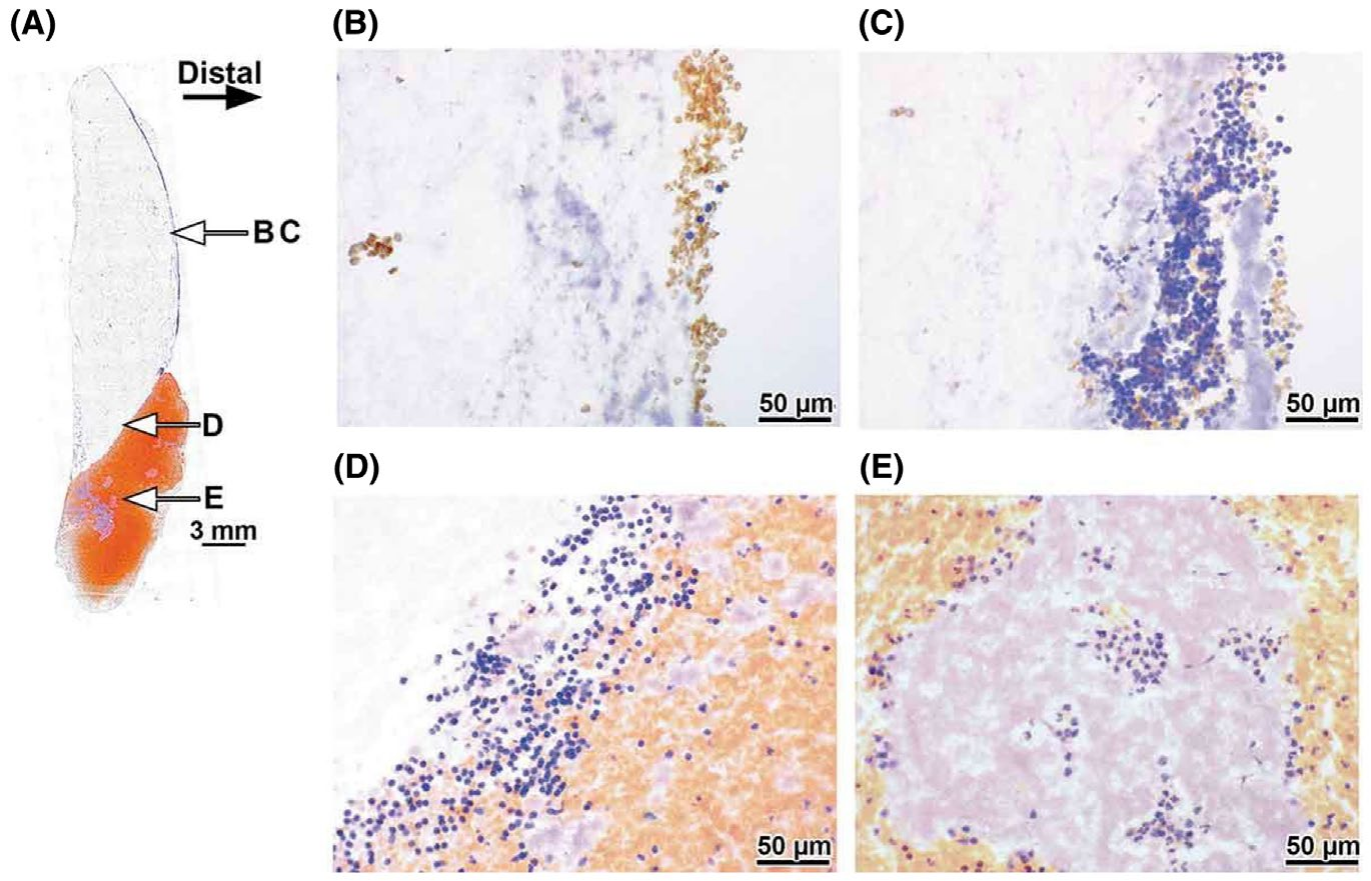
Histological observation of the frozen section of L‐PRF sectioned trans‐axially. (A) The panoramic view of the sections from the whole PRF clot including the RBC layer stained with hematoxylin. The L‐PRF clots and RBC layer were separated by a fixed‐angle. The distal wall showed two typical patterns shown in B and C. (B) A lot of RBCs with few leukocytes were located on fibrin networks on the distal surface. (C) The aggregated cluster consisting of platelets, leukocytes, and RBCs were occasionally observed. (D) Many leukocytes were located at the border between the PRF clot and the RBC layer. (E) The aggregated clusters of cells containing leukocytes were occasionally observed within the RBC layer within the red buffy coat zone. Reprinted with permission from Fujioka‐Kobayashi et al.^35^

**FIGURE 5:**
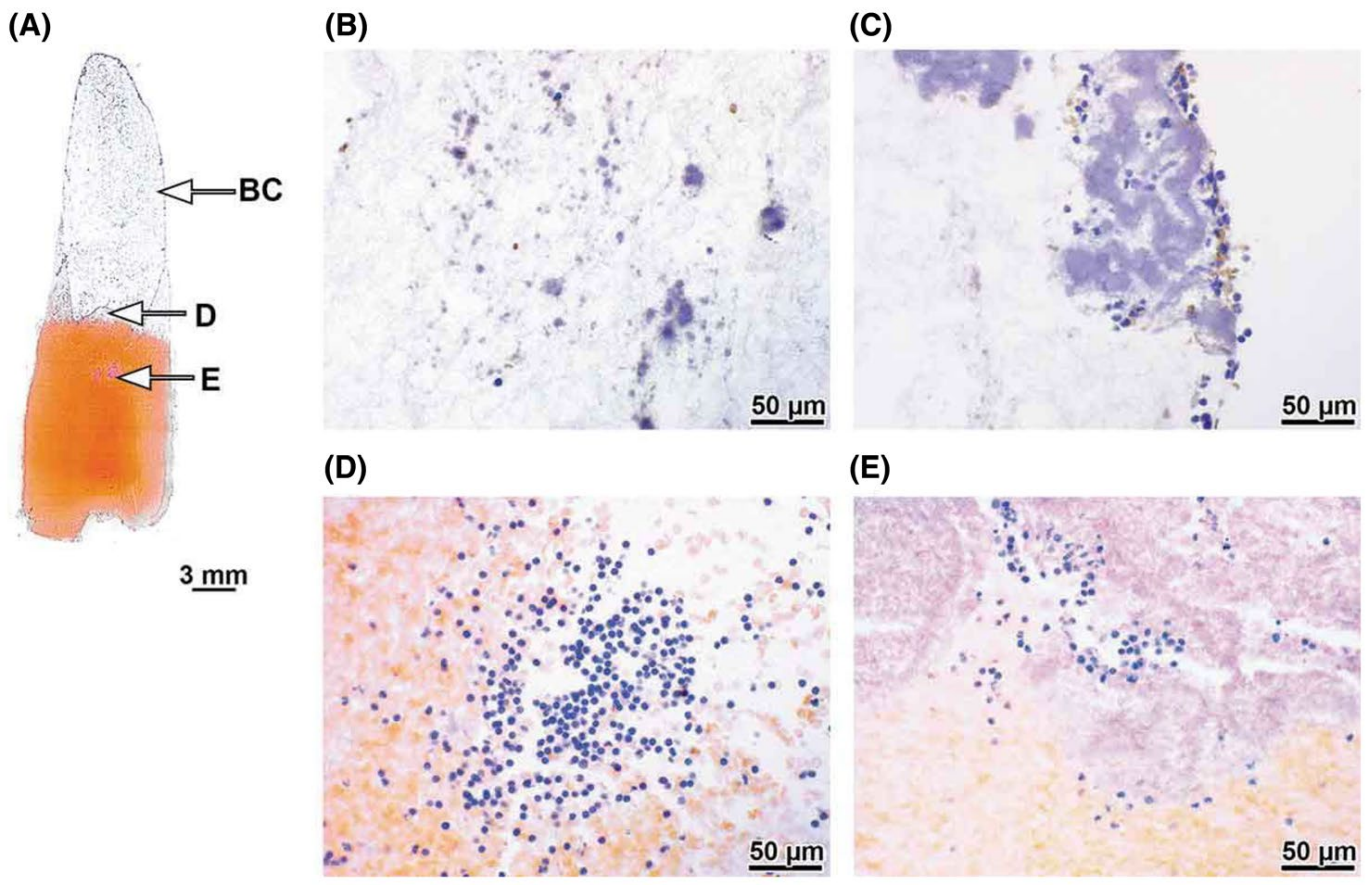
Histological observation of the frozen section of H‐PRF sectioned trans‐axially. (A) The panoramic view of the sections from the whole PRF clot including the RBC layer stained with hematoxylin. The H‐PRF clots and RBC layers were separated evenly and horizontally with no obvious accumulation of cells on the distal surface. The clots showed two typical patterns in B and C. (B) The fibrin networks were observed in the clots with many platelets with few leukocytes/RBCs. (C) The aggregated cluster consisting of leukocytes and a few RBCs were occasionally observed. (D) Many leukocytes were located at the border between the clots and the RBC layer. (E) The aggregated clusters of cells containing leukocytes were occasionally observed in the RBC layer within the red buffy coat zone. Reprinted with permission from Fujioka‐Kobayashi et al.^35^

**FIGURE 6:**
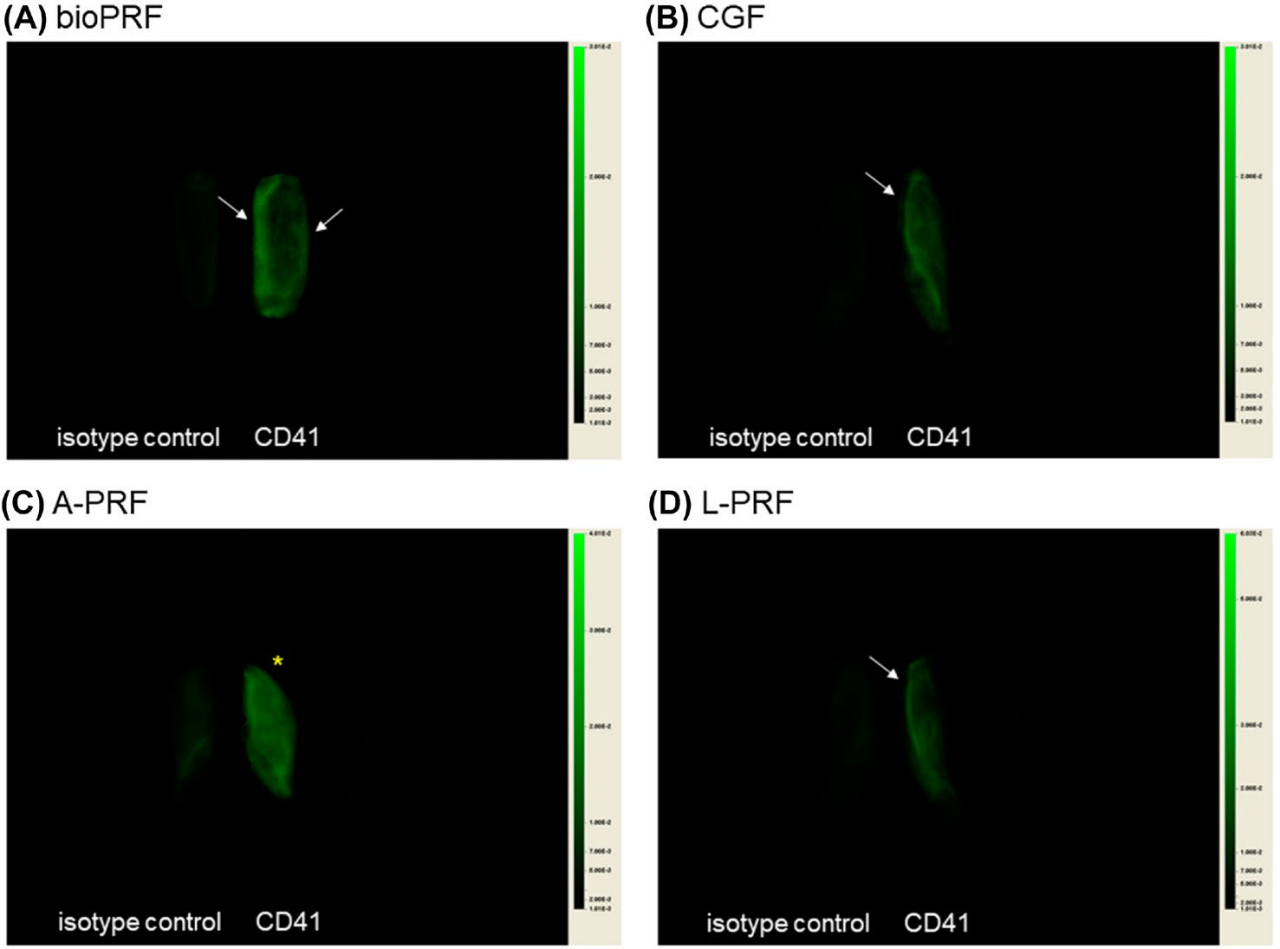
NIR images of compressed half PRF matrices: (A) bio‐PRF (horizontal, fast spin); (B) CGF (fixed angle, fast programmed spin); (C) A‐PRF (fixed angle, slow spin); (D) L‐PRF (fixed angle, fast spin). White arrows represent platelet localization. An asterisk denotes homogeneous platelet distribution. Reprinted from Aizawa et al.^41^

Fujioka‐Kobayashi et al.^35^ were the first to conduct a histological analysis of cells found within PRF following various protocols. Once again, it was observed histologically that L‐PRF clots prepared by fixed‐angle centrifugation exhibited a sloped boundary between the upper plasma and lower red blood cell (RBC) layers, with the majority of cells found at the buffy coat or along the back walls of PRF tubes (Figures 3 and 4).^35^ In contrast, clots produced by horizontal centrifugation displayed a smoother cell layer distribution along the tube surfaces, with platelets more evenly dispersed throughout the PRF clots (Figures 3 and 5).^35^ Similarly, Lourenço et al. used nuclei labeling to compare the cellular distribution of L‐PRF, prepared using fixed‐angle centrifugation, with Alb‐PRF, prepared using horizontal centrifugation.^48^ They observed that in the L‐PRF membrane, cell density increased progressively from the upper portion to the lower portion (buffy coat). In contrast, the Alb‐PRF membrane exhibited a more uniform cellular distribution across its entire length.^48^ Aizawa et al.^41^ also investigated platelet distribution across four PRF matrices (L‐PRF, A‐PRF, concentrated growth factor (CGF), and H‐PRF) using a non‐invasive near‐infrared (NIR) imaging method. Similarly, they found that in L‐PRF and CGF, platelets were primarily localized on and below the distal surface of the matrices, whereas in A‐PRF and H‐PRF, platelets were more evenly and widely distributed (Figure 6).^41^ In another study, Sato et al. examined the distribution of activated platelets within the same PRF matrices.^42^ They demonstrated similarly that, while platelets were distributed widely and evenly in H‐PRF and A‐PRF, they were localized at the distal surface of the tubes in L‐PRF and CGF.^42^

**FIGURE 7:**
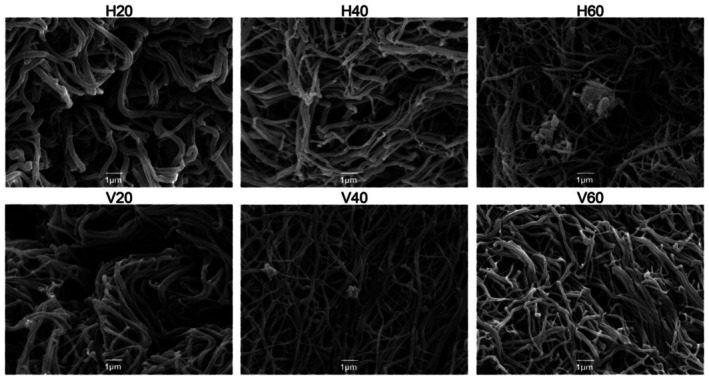
Electron micrographs, produced at ×2000 magnification, of the fibrin scaffolds produced with either vertical or horizontal rotor centrifugation, 20, 40, or 60 min after centrifugation. Reprinted with permission from Lourenço et al.^37^

One study conducted by Al‐Maawi et al. found little difference in cell distribution or density between fixed‐angle or horizontal centrifugation protocols.^45^ Lourenço et al. further assessed the impact of rotor angle (fixed‐angle vs. horizontal) and different storage times post‐centrifugation (20, 40, or 60 min) on cell content and the fibrin matrix.^37^ They found that cell content was consistent across all experimental groups, and the fibrin matrix showed no differences between groups (Figure 7).^37^ Similar results were observed in the blood samples of horses.^47^ In another study by Viswa et al., the fibrinogen content of PRF produced by a fixed‐angle centrifuge (DUO Quattro) was compared to that of PRF produced by two horizontal centrifuges (Remi C854 and Remi 8C), each using both a standard protocol and an RCF‐adjusted protocol.^50^ The results showed that PRF prepared using the RCF‐adjusted protocol with the Remi C854 centrifuge demonstrated high fibrinogen content similar to that obtained with a fixed‐angle centrifuge.^50^

#### 3.1.3 Growth factors and cytokines release

**FIGURE 8:**
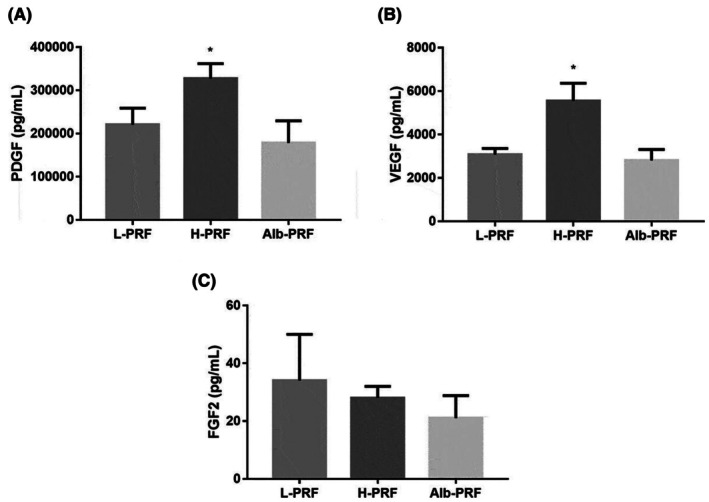
Assessment of the release of growth factors PDGF (A), VEGF (B), and FGF‐2 (C) from the L‐PRF, H‐PRF, and Alb‐PRF group, respectively, after 7 days of elution into cell culture media. Bars represent the mean ± SD of three biological and five technical replicates. An asterisk indicates a statistically significant difference between groups (*p* < 0.005). Reprinted with permission from Gheno et al.^36^

Four of the 13 studies investigated fixed‐angle versus horizontal centrifugation protocols on growth factor and cytokine release.^36^, ^37^, ^45^, ^48^ Lourenço et al. investigated the effects of rotor angle (fixed‐angle or horizontal) and varying storage times post‐centrifugation (20, 40, or 60 min) on the release of growth factors (fibroblasts growth factor 2 (FGF‐2), PDGF‐BB, and VEGF) and cytokines (interleukin 6 (IL‐6) and IL‐1β) over 21 days.^37^ They reported that horizontal centrifugation led to a twofold increase in PDGF‐BB and a 1.7‐fold increase in FGF‐2 release compared to fixed‐angle samples at day 21 in the 40‐min resting samples, while IL‐1β was significantly reduced in the 20‐min samples prepared using horizontal centrifugation.^37^ Additionally, Gheno et al. compared the release of growth factors (FGF‐2, PDGF, and VEGF) among three groups: L‐PRF produced by fixed‐angle centrifugation, and H‐PRF and Alb‐PRF produced by horizontal centrifugation, over 7 days.^36^ They found that while the release of FGF‐2 was similar across all three groups, the H‐PRF group exhibited a significantly higher release of VEGF and PDGF, with concentrations nearly twice that of the L‐PRF and Alb‐PRF groups (Figure 8).^36^ In another study, Lourenço et al. compared L‐PRF, prepared using fixed‐angle centrifugation, to Alb‐PRF, prepared using horizontal centrifugation.^48^ They demonstrated that Alb‐PRF released lower levels of inflammatory cytokines while exhibiting higher concentrations of regenerative growth factors such as PDGF and VEGF when compared to L‐PRF.^48^ In contrast, Al‐Maawi et al., compared the release of endothelial growth factor (EGF), VEGF, and TGF‐β1 from liquid PRF matrices prepared using either fixed‐angle or horizontal centrifugation protocols, demonstrated that no statistically significant differences were observed in the release of these growth factors over 7 days.^45^

#### 3.1.4 Antibacterial properties

**FIGURE 9:**
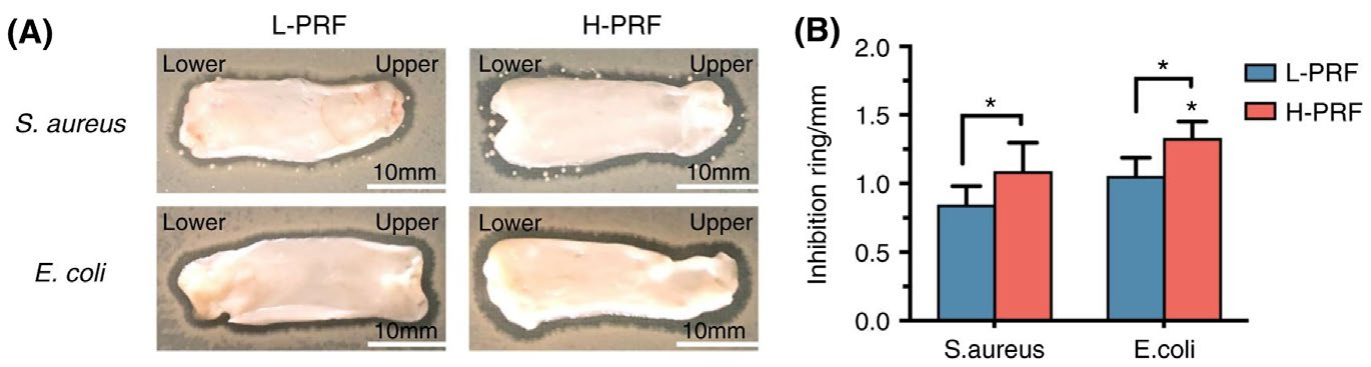
Antibacterial effects of L‐PRF and H‐PRF. (A, B) Photos and quantification of the inhibition zones of L‐PRF and H‐PRF membranes incubated with *S. aureus* or *E. coli* for 24 h. *p < 0.05, **p < 0.01, and ***p < 0.001. Reprinted from Feng et al.^43^

Only one study compared the antibacterial activity of PRF matrices produced by horizontal versus fixed‐angle centrifugation.^43^ In this study, Feng et al. demonstrated that while both PRFs exhibited notable antibacterial activity against *Staphylococcus aureus* and *Escherichia coli*, the H‐PRF group showed a significantly stronger antibacterial effect when compared to fixed‐angle (Figure 9).^43^ To further investigate whether the antibacterial properties varied across different layers, they divided the liquid‐state PRF into five equal portions after centrifugation in plastic tubes.^43^ They also measured the immune cell distribution within each layer, finding that the overall immune cell count in H‐PRF was 10‐fold higher than in fixed‐angle PRF, particularly in the upper layers. In the L‐PRF group, most immune cells were concentrated solely in the fifth layer, nearest to the buffy coat, whereas H‐PRF showed a more evenly distributed increase in immune cells across the upper layers.^43^

#### 3.1.5 Cellular functions

Two studies have examined the effects of PRF matrices produced by horizontal versus fixed‐angle centrifugation on cellular functions.^45^, ^48^ In the first study, Al‐Maawi et al. compared liquid PRF obtained from two horizontal centrifuges and one fixed‐angle centrifuge, focusing on cell viability and adhesion. Primary human osteoblasts and human fibroblasts were used in these experiments. The results showed no statistically significant differences in cell viability between the protocols. However, the number of adherent cells was significantly higher when treated with PRF obtained using the fixed‐angle rotor compared to those treated with PRF from horizontal rotors.^45^ Additionally, Lourenço et al. compared L‐PRF, prepared using fixed‐angle centrifugation, with Alb‐PRF, prepared using horizontal centrifugation.^48^ Their findings revealed that Alb‐PRF exhibited high biocompatibility across all tested concentrations (12.5%, 25%, 50%, and 100%), whereas L‐PRF demonstrated cytotoxic effects at concentrations exceeding 25%. Moreover, when osteoblasts were exposed to 25% extracts of the two biomaterials over 1–7 days, no significant differences were observed for proliferation between the L‐PRF and Alb‐PRF groups.^48^ They also demonstrated that Alb‐PRF resulted in increased early alkaline phosphatase (ALP) activity, enhanced in vitro mineralization, and a significantly higher osteoprotegerin (OPG)/receptor activator of nuclear factor kappa‐Β ligand (RANKL) mRNA ratio compared to L‐PRF.^48^

#### 3.1.6 Tissue reaction

Only one study evaluated the biocompatibility and degradation rate of PRF matrices produced using either horizontal or fixed‐angle centrifugation.^36^ In this study, Gheno et al. prepared L‐PRF using fixed‐angle centrifugation (700 *g* × 12 min) and H‐PRF and Alb‐PRF (700 *g* × 8 min) using horizontal centrifugation, assessing the anti‐inflammatory responses, biocompatibility, and degradation properties of PRF matrices after subcutaneous implantation over 7, 14, and 21 days.^36^ All groups exhibited excellent biocompatibility due to their autologous origin. However, it was notable that while both L‐PRF and H‐PRF membranes showed significant or complete resorption by day 21, the Alb‐PRF produced via horizontal centrifugation remained volume‐stable throughout the study period.^36^

#### 3.1.7 Bone regeneration

One study evaluated the bone regenerative potential of PRF matrices produced using either horizontal or fixed‐angle centrifugation. In this study, Ferreira Sávio et al. created critical‐size calvarial defects in thirty‐two rats and treated them with fixed‐angle L‐PRF (2700 rpm × 12 min), fixed‐angle A‐PRF (1500 rpm × 12 min), and horizontal H‐PRF (700 *g* × 8 min). They injected calcein and alizarin at 14 and 30 days to investigate the rate of bone growth and evaluated bone regeneration using micro‐computed tomography, laser confocal microscopy, and histomorphometric analyses.^46^ The results indicated that all experimental groups treated with L‐PRF, A‐PRF, and H‐PRF exhibited higher values for bone volume, newly formed bone area, and the precipitation of calcein and alizarin compared to the control group (which was treated with a blood clot). Notably, the H‐PRF group demonstrated significantly greater values for bone volume, the number of trabeculae, newly formed bone area, and higher precipitation of alizarin compared to all other groups.^46^

#### 3.1.8 Sinus floor augmentation

In a study by Viswa et al., PRF produced by a fixed‐angle centrifuge (DUO Quattro) was compared to PRF from two horizontal centrifuges (Remi C854 and Remi 8C), each evaluated using both a commonly utilized protocol (2700 rpm for 12 min) and an RCF‐adjusted protocol (400 *g* for 12 min) for indirect sinus elevation procedures.^50^ PRF produced using the second H‐PRF protocol resulted in the highest postoperative bone height gain (7.01 ± 1.44 mm) and bone fill (13.50 ± 4.51 mm^2^), surpassing the outcomes produced on the fixed‐angle device (6.82 ± 2.92 mm and 12.32 ± 5.31 mm^2^).^50^

#### 3.1.9 Periodontal regeneration

One study conducted by Shirakata et al. histologically compared the effects of various PRF protocols produced using fixed‐angle and horizontal centrifugation on gingival recession coverage and 2‐wall intrabony defect regeneration in beagle dogs.^44^ The recession defects were treated with a coronally advanced flap (CAF) alone, CAF combined with PRF produced via fixed‐angle centrifugation, or CAF with PRF produced via horizontal centrifugation (H‐PRF).^44^ For the two‐wall intrabony defects, the treatments included open flap debridement (OFD), OFD + L‐PRF, OFD + H‐PRF, and OFD + Alb‐PRF.^44^ Eight weeks after the second reconstructive surgery, the animals were euthanized for histological evaluation.^44^ In the defects treated with PRF, new bone and cementum formation occurred to varying extents, regardless of the PRF production protocol used. Notably, in the two‐wall intrabony defects, new bone formation extended from the host bone toward the coronal region of the defects in the sites treated with H‐PRF compared to those in the OFD, OFD + L‐PRF, and OFD + Alb‐PRF groups. The H‐PRF group also exhibited the greatest amount of newly formed cementum.^44^

#### 3.1.10 Overall comparative results

Of the 13 studies comparing horizontal to fixed‐angle centrifugation, 84.6% (11 studies) favored horizontal centrifugation, 15.4% (2 studies) reported no difference, and none favored fixed‐angle centrifugation over horizontal centrifugation (Table 3).

**TABLE 3:** Summary of the outcomes from the included comparative studies, highlighting which centrifugation method demonstrated superior performance overall.

Authors	Horizontal centrifugation	Fixed‐angle centrifugation	No difference
Miron et al.^40^ (2019)	✓		
Aizawa et al.^41^ (2020)	✔		
Lourenço et al.^37^ (2020)	✔		
Sato et al.^42^ (2020)	✔		
Feng et al.^43^ (2020)	✔		
Viswa et al.^50^ (2020)	✔		
Fujioka‐Kobayashi et al.^35^ (2021)	✔		
Shirakata et al.^44^ (2021)	✔		
Gheno et al.^36^ (2021)	✔		
Al‐Maawi et al.^45^ (2022)			✔
Ferreira Sávio et al.^46^ (2023)	✔		
Chiara et al.^47^ (2024)			✔
Lourenço et al.^48^ (2024)	✔		

### 3.2 Section 2: Use of horizontal centrifugation in additional non‐comparative studies

In addition to the comparative studies, 62 studies exclusively used horizontal centrifugation to produce novel data that further extend its uses and clinical applications. These were categorized into the following sections: (1) centrifugation protocols; (2) concentrated platelet‐rich fibrin (C‐PRF); (3) albumin gel with liquid platelet‐rich fibrin (Alb‐PRF); (4) growth factor and cytokine release; (5) anti‐inflammatory properties; (6) antibacterial properties; (7) wound healing and soft tissue regeneration; (8) oral cancer treatment; (9) cartilage regeneration; (10) orthopedic treatment; (11) bone regeneration; (12) sinus floor augmentation; (13) dental implant; (14) Treatment of palatogingival groove; (15) treatment of external root resorption and radicular cysts; (16) bone healing after tooth hemisection; (17) tooth autotransplantation; (18) regenerative endodontic treatment; (19) drug delivery; (20) corneal regeneration; (21) skin and hair regeneration; (22) myringoplasty; and (23) further optimization of H‐PRF. Through this, we aimed to provide deeper insights into the properties and potential applications of H‐PRF in regenerative medicine and dentistry based on current evidence. Table 4 presents a summary of these studies.

**TABLE 4:** Summary of the additional non‐comparative studies using horizontal centrifugation.

Authors (Year)	Aim of study	Type of study	Blood source	Study groups	Horizontal centrifugation group(s): preparation method (centrifuge device)	Main measured variable(s): assessment method(s)	Main outcome(s)
*Centrifugation protocols*							
Miron et al.^38^ (2020)	To evaluate 24 protocols for the production of PRF produced via horizontal centrifugation to better understand cell separation following centrifugation at various times and speeds	In vitro study	Human	(1) H‐PRF	(1) H‐PRF: 100 *g* × 3/5/8/12 min 200 *g* × 3/5/8/12 min 400 *g* × 3/5/8/12 min 700 *g* × 3/5/8/12 min 1000 *g* × 3/5/8/12 min 1200 *g* × 3/5/8/12 min (5702 Eppendorf Centrifuge, Germany)	Quantification of cells: Hematology analyzer	The protocol of 700g for 8 min achieved the highest yield of platelets and leukocytes, evenly distributed throughout the upper layers of the H‐PRF
Feng et al.^51^ (2023)	To determine the optimal centrifugation force for preparing liquid PRF using horizontal centrifugation	In vitro study	Human	(1) Liquid H‐PRF	(1) Liquid H‐PRF: 100 *g* × 8 min 300 *g* × 8 min 500 *g* × 8 min 700 *g* × 8 min (Plasmatrident Centrifuge, China)	PRF volume, weight, and solidification time Mechanical properties: Tensile testing machine Structural analysis: Scanning electron microscopy Rheologic properties: Stress‐controlled rheometer Quantification of cells: Hematology analyzer Distribution of cells: Histology	Complete blood count analysis revealed that the 500 *g* group had the highest number of leukocytes and neutrophils, whereas the 100 *g* group yielded the highest concentration of leukocytes and platelets. Additionally, histological analysis suggested that cells obtained at 500 *g* for 8 min were the most evenly distributed in liquid H‐PRF
Sneha et al.^52^ (2022)	To evaluate the impact of the original and modified centrifugation protocols (adjusted to ~400 *g*) on platelet concentration, clot size, and growth factor release in L‐PRF, using two commercially available horizontal centrifuges, with assessments conducted at 20, 40, and 60 min of resting time	In vitro study	Human	(1) L‐PRF (Original Protocol) (2) L‐PRF (Modified Protocol) (3) L‐PRF (Original Protocol) (4) L‐PRF (Modified Protocol)	(1) L‐PRF (Original Protocol): 2700 rpm × 12 min (REMI 8C Centrifuge, India) (2) L‐PRF (Modified Protocol): 400 *g* (1690 rpm) × 12 min (REMI 8C Centrifuge, India) (3) L‐PRF (Original Protocol): 2700 rpm × 12 min (REMI C854 Centrifuge, India) (4) L‐PRF Modified Protocol: 400 *g* (1650 rpm) × 12 min (REMI C854 Centrifuge, India)	PRF size Quantification of growth factor release: ELISA Quantification of cells: Histology	This study demonstrated that the centrifuge type and relative centrifugal force significantly influenced the quality and quantity of cells and growth factors. Maintaining an optimal balance between g‐force/rpm was crucial to producing H‐PRF with adequate cell viability and optimal growth factor release
*Concentrated Platelet‐rich Fibrin (C‐PRF)*							
Fujioka‐Kobayashi et al.^53^ (2020)	To investigate the regenerative properties and effects on growth factor release and cellular activity of C‐PRF compared to standard i‐PRF protocols	In vitro study	Human	(1) i‐PRF (2) C‐PRF	(1) i‐PRF: 300 *g* × 5 min (5702 Eppendorf Centrifuge, Germany) (2) C‐PRF: 3000 *g* × 8 min (5702 Eppendorf Centrifuge, Germany)	Quantification of cells: Hematology analyzer Quantification of growth factor release: ELISA Cell viability: Live/Dead assay Cell migration: Transwell assay Cell proliferation: Luminescent cell viability assay Gene and protein expression: RT‐PCR Immunofluorescence staining	C‐PRF, collected specifically from the buffy coat layer using higher horizontal centrifugation protocols, demonstrated up to a threefold increase in growth factor release compared to standard i‐PRF. This notably enhanced gingival fibroblast migration, proliferation, gene expression, and collagen I synthesis
Kargarpour et al.^54^ (2020)	To compare the TGF‐β activity in the lysates of PRF membranes produced by horizontal centrifugation using tubes with clot activators at different g‐forces	In vitro study	Human	(1) Three different types of H‐PRF	(1) H‐PRF: 210 *g* × 12 min 650 *g* × 12 min 1500 *g* × 12 min (Z306 Hermle Universal Centrifuge, Germany)	Gene and protein expression: RT‐PCR Western blot Immunofluorescence staining	H‐PRF exhibited significantly higher TGF‐β activity when harvested from the buffy coat fraction across both high‐speed protocols (650 *g* and 1500 *g*) when compared to the PPP group
Kargarpour et al.^55^ (2022)	To evaluate the distribution of fibrinogen in liquid H‐PRF	In vitro study	Human	(1) Three different types of liquid H‐PRF	(1) Liquid H‐PRF: 300 *g* × 8 min 700 *g* × 8 min 2000 *g* × 8 min (Z306 Hermle Universal Centrifuge, Germany)	Fibrinogen concentration: ELISA Clot weight: Weight measurement	The findings suggested that PPP serves as the primary source of clottable fibrinogen, whereas the buffy coat layer primarily provided cells
*Albumin Gel with Liquid Platelet‐rich Fibrin (Alb‐PRF)*							
Fujioka‐Kobayashi et al.^56^ (2021)	To perform histological evaluation including the distribution of cells within Alb‐PRF and to characterize the release of growth factors, cell biocompatibility, migration potential, proliferation assay, and expression of TGF‐β1 and collagen 1 were investigated in human gingival fibroblasts	In vitro study	Human	(1) Alb‐PRF (2) Control	(1) Alb‐PRF: 700 *g* × 8 min + PPP (heating for 10 min at 75°C) (5702 Eppendorf Centrifuge, Germany)	Distribution of cells and structural analysis: Histology Quantification of growth factor release: ELISA Cell viability: Live/Dead assay Cell migration: Transwell assay Cell proliferation: Luminescent cell viability assay Gene and protein expression: RT‐PCR Immunofluorescence staining	Histological analysis showed an even distribution of viable cells in Alb‐PRF. Growth factor release, especially TGF‐β1 and PDGF‐AA/AB, was gradually released over a 10‐day period. Alb‐PRF also demonstrated higher cell biocompatibility at 24 h and enhanced fibroblast proliferation at 5 days compared to the control. It also induced higher mRNA expression of TGF‐β at 3 and 7 days, and collagen 1 at 7 days
Del Pont and Zupi^57^ (2024)	To evaluate the effect of Alb‐PRF on vestibuloplasty	Case report	Human	(1) Alb‐PRF	(1) Alb‐PRF: 700 *g* × 8 min + PPP (heating for 10 min at 75°C) (Zhejiang Gongdong Medical Technology Co., China)	Tissue healing and regeneration: Clinical examination	Alb‐PRF application resulted in an increase in vestibular depth and keratinized tissue width, after nine weeks
Lima Barbosa et al.^58^ (2024)	To evaluate the association of Alb‐PRF with nanostructured carbonated hydroxyapatite microspheres (ncHAp) and to establish a comparison with the Alb‐PRF membrane without the spheres	In vitro study	Human	(1) Alb‐PRF (2) Alb‐ncHAp‐PRF	(1) Alb‐PRF: 700 *g* × 8 min + PPP (heating for 10 min at 75°C) (Bio‐PRF Centrifuge, USA)	Structural analysis: Scanning electron microscopy Distribution of cells and structural analysis: Histology Quantification of growth factor release: Multiplex assay Cell viability: Live/Dead assay XTT assay Cell proliferation: Crystal violet test Mineralization: ALP activity test Alizarin red staining	These findings suggest that the addition of ncHAp spheres reduced the biological activity of Alb‐PRF, impairing its initial effects on osteoblast behavior
Zeng et al.^59^ (2024)	To explore the feasibility of constructing cartilage tissue engineering using ALB–PRF combined with chondrocytes	In vitro and in vivo study	Human	(1) Alb‐PRF	(1) Alb‐PRF: 700 *g* × 8 min + PPP (heating for 10 min at 75°C) (5702 Eppendorf Centrifuge, Germany)	Structural analysis: Scanning electron microscopy Quantification of growth factor release: ELISA Cell proliferation: EdU assay CCK‐8 assay Cell migration: Transwell assay Cell adhesion: H&E staining Gene and protein expression: RT‐PCR Western blot GAG expression: Alcian blue staining Cartilage regeneration: Histology Immunohistochemistry	Alb‐PRF was shown to exhibit a porous structure and a slow release of growth factors over time. It also enhanced the proliferation, migration, adhesion, phenotype maintenance, and extracellular matrix secretion of rabbit chondrocytes. Alb‐PRF also promoted in vivo chondrogenesis, producing regenerative cartilage that was histologically like natural knee joint cartilage
*Growth factors and cytokines release*							
Lourenço et al.^60^ (2018)	To evaluate the morphological characteristics and in vitro structural stability of leukocyte‐ and platelet‐ rich fibrin membranes produced with horizontal rotor centrifuges, as well as their capacity of releasing growth factors, as well as pro‐ and anti‐inflammatory cytokines, during the first weeks after production	In vitro study	Human	(1) H‐PRF	(1) H‐PRF: 400 *g* × 10 min (B‐40 Centrifuge, Brazil)	Structural analysis: Scanning electron microscopy Cell distribution: Fluorescence microscopy Quantification of growth factor and cytokine release: Multiplex assay Structural stability: Photographic recordings	The results showed that blood‐derived fibrin membranes with high structural stability and cell content can be produced using horizontal centrifugation, capable of sustaining the prolonged release of growth factors and pro‐ and anti‐inflammatory cytokines
*Anti‐inflammation properties*							
Kargarpour et al.^61^ (2020)	To examine whether standard H‐PRF, non‐heated and heated PPP, as well as the buffy coat layer of H‐PRF, could neutralize the toxic concentration of exogenous hydrogen peroxide	In vitro Study	Human	(1) H‐PRF (2) PPP (3) Buffy Coat (C‐PRF) (4) Heated PPP (Albumin Gel) (5) Blood Clot	(1) H‐PRF: 400 *g* × 12 min (Z306 Hermle Universal Centrifuge, Germany) (2) PPP and Buffy Coat (C‐PRF): 700 *g* × 8 min (NR) (3) Heated PPP (Albumin Gel): 700 *g* × 8 min + Heating for 10 min at 75°C (NR)	Cell viability: MTT assay Trypan blue staining Live/Dead assay Oxygen measurement: Bubble assay Gene and protein expression: RT‐PCR Western blot	The results showed that lysates from H‐PRF, PPP and the buffy coat layer of H‐PRF effectively neutralized hydrogen peroxide toxicity, whereas heated PPP did not exhibit this effect
Kargarpour et al.^62^ (2020)	To investigate the in vitro TGF‐β activity of all fractions of Alb‐PRF	In vitro Study	Human	(1) Buffy Coat (C‐PRF) (2) PPP (3) Heated PPP (Albumin Gel) (4) Red Clot	(1) PPP, Buffy Coat (C‐PRF), and Red Clot: 700 *g* × 8 min (Z306 Hermle Universal Centrifuge, Germany) (2) Heated PPP (Albumin Gel): 700 *g* × 8 min + Heating for 10 min at 75°C (Z306 Hermle Universal Centrifuge, Germany)	Gene and protein expression: RT‐PCR Western blot Immunoassay Immunofluorescence staining	The findings revealed that both the cell‐rich buffy coat and PPP layers contained TGF‐β activity, which is, however, heat‐sensitive
Nasirzadeh et al.^63^ (2020)	To evaluate the effect of H‐PRF on macrophage polarization.	In vitro Study	Human	(1) H‐PRF	(1) H‐PRF: 400 *g* (1570 rpm) × 12 min (Z306 Hermle Universal Centrifuge, Germany)	Gene and protein expression: RT‐PCR Immunoassay Immunofluorescence staining	The results indicated that H‐PRF exhibits anti‐inflammatory activity and promotes the polarization of macrophages from an M1 to an M2 phenotype
Li et al.^64^ (2023)	To explore the potential role of liquid H‐PRF in managing inflammation, with a comparison to the widely used HA treatments	In vitro study	Human	(1) Liquid H‐PRF (2) HA	(1) Liquid H‐PRF: 500 *g* × 8 min (NR)	Gene and protein expression: RT‐PCR Western blot	Liquid H‐PRF exhibited the capability to reduce inflammatory levels in chondrogenic cells, with this effect further enhanced when PRF from the buffy coat zone was included, when compared to the HA group.
Kargarpour et al.^65^ (2021)	Murine cells (ST2 mesenchymal stromal and 3T3‐L1 preadipocyte cells) and gingival fibroblasts were used to investigate the possible anti‐inflammatory activity of various H‐PRF preparations in vitro	In vitro study	Human	(1) Buffy Coat (C‐PRF) (2) PPP (3) Heated PPP (Albumin Gel) (4) Red Clot (5) H‐PRF	(1) PPP, Buffy Coat (C‐PRF), and Red Clot: 2000 *g* × 8 min (Z306 Hermle Universal Centrifuge, Germany) (2) Heated PPP (Albumin Gel): 2000 *g* × 8 min + Heating for 10 min at 75°C (Z306 Hermle Universal Centrifuge, Germany) (3) H‐PRF: 400 *g* (1570 rpm) × 12 min (Z306 Hermle Universal Centrifuge, Germany)	Gene and protein expression: RT‐PCR Western blot Immunoassay Immunofluorescence staining	These findings indicated that both liquid and solid H‐PRF exhibit strong anti‐inflammatory properties in murine mesenchymal cells but not in human gingival fibroblasts or epithelial cells
Kargarpour et al.^66^ (2022)	To compare lysates prepared from H‐PRF and UBC based on bioassays and degradation of the respective membranes	In vitro study	Human	(1) H‐PRF (2) UBC	(1) H‐PRF: 700 *g* × 8 min (Z306 Hermle Universal Centrifuge, Germany)	Mechanical properties: Compression test Gene and protein expression: RT‐PCR Western blot Immunoassay Immunofluorescence staining	The findings suggested that while both UBC and H‐PRF exhibit strong TGF‐β and anti‐inflammatory activity, UBC lacks the structural strength required for the clinical preparation of functional membranes
Kargarpour et al.^67^ (2023)	To investigate whether the lipid fraction of H‐PRF has anti‐inflammatory properties	In vitro study	Human	(1) Buffy Coat (C‐PRF) (2) PPP (3) Red Clot (4) PRF	(1) PPP, Buffy Coat (C‐PRF), and Red Clot: 2000 *g* × 8 min (Z306 Hermle Universal Centrifuge, Germany) (2) H‐PRF: 400 *g* (1570 rpm) × 12 min (Z306 Hermle Universal Centrifuge, Germany)	Gene and protein expression: RT‐PCR Western blot Immunoassay	The findings indicated that the lipid fraction is at least partially responsible for the anti‐inflammatory activity of H‐PRF
*Antibacterial properties*							
Qiu et al.^68^ (2023)	To compare the barrier function during bacterial invasion of 3 commonly used membranes including H‐PRF against two commercially available resorbable collagen membranes	In vitro study	Human	(1) H‐PRF (2) Bio‐Gide Collagen Membrane (Gesitlich) (3) Megreen Collagen Membrane (Shanxi Ruisheng, Biotechnology Co)	(1) H‐PRF: 700 *g* × 8 min (Plasmatrident Centrifuge, China)	Antibacterial activity: CFU counting assay after permeability experiment Scanning electron microscopy after permeability experiment	H‐PRF membranes demonstrated superior barrier function against *S. aureus* during 2 days of inoculation when compared to collagen membranes
*Wound healing and soft tissue regeneration*							
Qiu et al.^68^ (2023)	To compare the wound healing properties of 3 commonly used membranes including H‐PRF against two commercially available resorbable collagen membranes	In vitro study	Human	(1) H‐PRF (2) Bio‐Gide Collagen Membrane (Gesitlich) (3) Megreen Collagen Membrane (Shanxi Ruisheng, Biotechnology Co) (4) Control group	(1) H‐PRF: 700 *g* × 8 min (Plasmatrident Centrifuge, China)	Wound healing: Scratch wound healing assay	The wound healing assay demonstrated significantly better wound closure rates in the H‐PRF group when compared to natural healing
Qiu et al.^69^ (2024)	To evaluate the effect of combined CHA and H‐PRF on human gingival fibroblasts	In vitro study	Human	(1) Liquid H‐PRF (2) CHA (3) CHA + Liquid H‐PRF (CHA‐PRF) (4) Control group	(1) Liquid H‐PRF: 500 *g* × 8 min (Plasmatrident Centrifuge, China)	Coagulation time Cell viability: CCK‐8 assay Cell migration: Scratch would healing assay Transwell assay Gene and protein expression: RT‐PCR Immunofluorescence staining	The CHA‐PRF group showed a greater potential to promote soft tissue regeneration by stimulating cell proliferation, collagen synthesis, and migration in human gingival fibroblasts compared to the pure CHA or H‐PRF group
Imani et al.^70^ (2024)	To evaluate the activity retained in H‐PRF membranes versus what is released in H‐PRF serum	In vitro study	Human	(1) H‐PRF (2) H‐PRF Serum	(1) H‐PRF and H‐PRF Serum: 700 *g* × 8 min (Z306 Hermle Universal Centrifuge, Germany)	Gene expression: RNA sequencing	H‐PRF membrane lysates, compared to H‐PRF serum, induced a more complex response towards gingival fibroblasts, although both increased chemokine expression in these cells
*Oral cancer treatment*							
Afradi et al.^71^ (2024)	To examine the impact of lysates derived from solid H‐PRF membranes on inflammation in oral squamous carcinoma cell lines (HSC2 and TR146) and primary oral epithelial cells	In vitro study	Human	(1) H‐PRF	(1) H‐PRF: 700 *g* × 8 min (Z306 Hermle Universal Centrifuge, Germany)	Gene and protein expression: RT‐PCR Western blot Immunoassay Immunofluorescence staining	The results suggested that H‐PRF may decrease inflammation in a malignant environment while eliciting an immunological response in healthy oral epithelium
*Cartilage regeneration*							
Li et al.^64^ (2023)	To investigate the effects of liquid H‐PRF on chondrocyte proliferation and cartilage regeneration, with a comparison to the widely used HA treatments	In vitro study	Human	(1) Liquid H‐PRF (2) HA	(1) Liquid H‐PRF: 500 *g* × 8 min (NR)	Cell morphology: Light microcopy Cell viability: CCK‐8 assay Cell Proliferation: EdU assay GAG expression: Toluidine blue and alcian blue staining Gene and protein expression: RT‐PCR Western blot Immunofluorescence staining	Liquid H‐PRF had significant effects on chondrocytes, impacting their proliferation, inflammatory responses, and chondrogenic differentiation. The H‐PRF group showed significantly higher expression of chondrogenic markers, such as collagen 2a1, compared to cells treated with HA, while aggrecan expression was notably higher in the HA group
*Orthopedic treatment*							
Ogéus et al.^72^ (2024)	To evaluate the regenerative potential of stromal vascular fraction combined with H‐PRF matrices as a novel therapeutic approach for managing hip and knee osteoarthritis, based on a 2‐year follow‐up of 104 patients	Clinical study	Human	(1) Stromal vascular fraction + C‐PRF + Alb‐PRF	(1) C‐PRF: 2000 *g* × 8 min (Bio‐PRF Centrifuge, USA) (2) Alb‐PRF: 2000 *g* × 8 min + PPP (heating for 10 min at 75°C) (Bio‐PRF Centrifuge, USA)	Tissue healing and regeneration: WOMAC Osteoarthritis Index Radiography	Significant improvements in WOMAC scores were observed for both hip and knee osteoarthritis. Radiographic evaluations further revealed an increase in joint space, with an average gain of 2 mm in knee joints and 1.6 mm in hip joints. Overall patients reported significantly lower pain scores even 2 years following joint injections
Ogéus et al.^73^ (2024)	To evaluate the effectiveness of H‐PRF matrices—a combination of Liquid H‐PRF, C‐PRF, and Alb‐PRF—in patients diagnosed with lateral epicondylitis	Clinical study	Human	(1) Liquid H‐PRF + C‐PRF + Alb‐PRF	(1) Liquid H‐PRF: 300 *g* × 5 min (Bio‐PRF Centrifuge, USA) (2) C‐PRF: 300 *g* × 5 min + 2000 *g* × 4 min (Bio‐PRF Centrifuge, USA) (3) Alb‐PRF: 2000 *g* × 8 min + PPP (heating for 10 min at 75°C) (Bio‐PRF Centrifuge, USA)	Pain perception: Numerical rating scale Tissue healing and regeneration: Sonography	The findings demonstrated that H‐PRF matrices significantly reduced NRS pain scores and enhanced clinical orthopedic test results. Furthermore, sonographic imaging revealed substantial improvements within 3 months post‐treatment. These outcomes, supported by 1‐year follow‐up data, indicate notable long‐term benefits
Ogéus et al.^74^ (2024)	To evaluate the effect of H‐PRF matrices (C‐PRF and Alb‐PRF) in a patient with Osgood‐Schlatter disease	Case report	Human	(1) C‐PRF + Alb‐PRF	(1) C‐PRF: 2000 *g* × 8 min (Bio‐PRF Centrifuge, USA) (2) Alb‐PRF: 2000 *g* × 8 min + PPP (heating for 10 min at 75°C) (Bio‐PRF Centrifuge, USA)	Pain perception: Visual analogue scale Tissue healing and regeneration: Clinical examination	The patient reported a significant reduction in pain levels 3 months post‐treatment and showed no palpation pain during the examination. Two months after treatment, the patient demonstrated near‐complete remission of patellar tendon ossification and was able to resume sports after a 3‐year break
Ogéus et al.^75^ (2024)	To evaluate the effect of H‐PRF matrices (C‐PRF and Alb‐PRF) in a patient with a fractured humerus and partially torn supraspinatus tendon	Case report	Human	(1) C‐PRF + Alb‐PRF	(1) C‐PRF: 2000 *g* × 8 min (Bio‐PRF Centrifuge, USA) (2) Alb‐PRF: 2000 *g* × 8 min + PPP (heating for 10 min at 75°C) (Bio‐PRF Centrifuge, USA)	Tissue healing and regeneration: Clinical examination Radiography	Within 1 month, the patient showed near‐complete recovery from both injuries and was able to return to professional boxing after 3 months
Ogéus et al.^76^ (2024)	To evaluate the effect of intra‐articular injections of H‐PRF matrices (C‐PRF and Alb‐PRF), SVF, and amniotic‐derived exosomes in patients with bone and cartilage degeneration	Case series	Human	(1) C‐PRF + Alb‐PRF + SVF + Exosomes	(1) C‐PRF: 2000 *g* × 8 min (Bio‐PRF Centrifuge, USA) (2) Alb‐PRF: 2000 *g* × 8 min + PPP (heating for 10 min at 75°C) (Bio‐PRF Centrifuge, USA)	Pain perception: Verbal rating scale Tissue healing and regeneration: Clinical examination Radiography	Patients reported a significant reduction in pain and an improvement in physical function during the follow‐up period after the joint injection
*Bone regeneration*							
Apaza Alccayhuaman et al.^77^ (2024)	To compare the effects of human and rat C‐PRF prepared using horizontal centrifugation on the osteoconductive properties of resorbable collagen membranes	In vivo study	Human and Animal (Rat)	(1) Human C‐PRF (2) Rat C‐PRF	(1) C‐PRF 2000 *g* × 8 min (Z306 Hermle Universal Centrifuge, Germany)	Tissue healing and regeneration: Micro‐computed tomography Histomorphometry	Rat PRF promoted new bone growth and hybrid bone formation with collagen fibers, while human PRF lacked new bone formation in the defect center and showed minimal bone at the margins. Rat PRF also produced significantly higher bone volume than human PRF
Kargarpour et al.^78^ (2020)	To elucidate the cellular and molecular mechanism by which H‐PRF supports ridge preservation	In vitro study	Human	(1) H‐PRF	(1) H‐PRF: 400 *g* (1570 rpm) × 12 min (Z306 Hermle Universal Centrifuge, Germany)	Cell viability and proliferation: Formazan formation assay Live/Dead assay BrdU assay Caspase‐3 activity assay Histochemical analysis: TRAP staining Pit formation assay Gene expression: RT‐PCR	The findings suggested that H‐PRF membranes can inhibit osteoclast formation from hematopoietic progenitors in bone marrow cultures. However, H‐PRF membranes cannot reverse osteoclastogenesis once it has initiated/progressed
Kargarpour et al.^79^ (2021)	To evaluate the effects of liquid H‐PRF on inflammation and osteoclast formation	In vitro study	Human	(1) Buffy Coat (C‐PRF) (2) PPP (3) Heated PPP (Albumin Gel) (4) Red Clot	(1) PPP, Buffy Coat (C‐PRF), and Red Clot: 2000 *g* × 8 min (Z306 Hermle Universal Centrifuge, Germany) (2) Heated PPP (Albumin Gel): 2000 *g* × 8 min + Heating for 10 min at 75°C (Z306 Hermle Universal Centrifuge, Germany)	Gene and protein expression: RT‐PCR Western blot Immunoassay Immunofluorescence staining Histochemical staining	Liquid H‐PRF exhibited potent, heat‐sensitive anti‐inflammatory activity in macrophages in vitro, which is associated with the inhibition of osteoclastogenesis
Khan et al.^80^ (2023)	To clinically compare a commercially available combination of 70:30 nHAp and β‐TCP along with H‐PRF with DFDBA along with H‐PRF in small maxillofacial osseous defects	Clinical study	Human	(1) nHAp + β‐TCP + H‐PRF (2) DFDBA + H‐PRF	(1) H‐PRF: 3000 rpm × 10 min (REMI Centrifuge, NR)	Pain perception: Visual analogue scale Presence of swelling, dehiscence, and infection: Clinical examination Graft fate: MDP ^99m^Tc scan	No significant difference was observed between the two groups for any of the parameters. However, significant improvements were noted for pain and swelling in both groups at various intervals. The MDP ^99m^Tc scan also showed increased tracer uptake in the representative patient of each group
Anaya‐Sampayo et al.^81^ (2024)	To develop scaffolds composed of nHAp, GEL, and CH, with or without ALG and lyophilized H‐PRF, to evaluate the scaffold's properties, growth factor release, and DPSCs, and OB derived from DPSCs viability	In vitro study	Human	(1) nHAp‐CH‐GEL (2) nHAp‐CH‐GEL + H‐PRF (3) nHAp‐CH‐GEL‐ALG (4) nHAp‐CH‐GEL‐ALG + H‐PRF	(1) H‐PRF: 1019 *g* (2700 rpm) × 12 min (Thermo Scientific Heraeus Primo R Centrifuge, NR)	Scaffold size and pore morphology: Scanning electron microscopy Elemental analysis composition: Energy dispersive X‐ray spectroscopy Swelling and degradation profile: Weight calculation Quantification of growth factor release: ELISA Cytotoxicity: MTS assay Cell viability: MTT assay	The nHAp‐CH‐GEL‐PRF scaffold exhibited optimal physical and biological characteristics for enhancing DPSCs and OB‐DPSCs cell viability. Therefore, it was suggested that lyophilized H‐PRF enhanced the scaffold's biocompatibility for bone tissue regeneration
Kargarpour et al.^82^ (2021)	To investigate whether solid H‐PRF could enhance BMP‐2 expression in mesenchymal cells during bone regeneration	In vitro study	Human	(1) H‐PRF (2) Buffy Coat (C‐PRF) (3) PPP (4) Heated PPP (Albumin Gel) (5) Red Clot	(1) H‐PRF, PPP, Buffy Coat (C‐PRF), and Red Clot: 700 *g* × 8 min (Z306 Hermle Universal Centrifuge, Germany) (2) Heated PPP (Albumin Gel): 700 *g* × 8 min + Heating for 10 min at 75°C (Z306 Hermle Universal Centrifuge, Germany)	Gene and protein expression: Proteomics RT‐PCR Western blot Immunoassay Immunofluorescence staining	The data suggested that H‐PRF could activate TGF‐β receptor 1 kinase, thereby inducing the production of BMP‐2 in mesenchymal lineage cells
*Sinus floor augmentation*							
Yu et al.^83^ (2022)	To prepare and apply H‐PRF bone block in a rabbit maxillary sinus augmentation model and to investigate the potential benefits of H‐PRF bone block on bone formation through microcomputed examination and Histomorphometric evaluation	In vivo study	Animal (Rabbit)	(1) DBBM (2) DBBM + H‐PRF	(1) Solid H‐PRF: 700 *g* × 8 min (5702 Eppendorf Centrifuge, Germany) (1) Liquid H‐PRF: 700 *g* × 8 min (5702 Eppendorf Centrifuge, Germany)	Tissue healing and regeneration: Micro‐computed tomography Histomorphometry	H‐PRF + DBBM (H‐PRF bone block) demonstrated superior potential for sinus augmentation compared to DBBM alone, enhancing angiogenesis, bone formation, and bone remodeling in a rabbit model
Yu et al.^84^ (2023)	To evaluate the early tissue and healing responses during maxillary sinus grafting using H‐PRF bone blocks compared to DBBM alone using a rabbit model	In vitro and in vivo study	For in vitro: Human For in vivo: Animal (Rabbit)	(1) DBBM (2) DBBM + H‐PRF	(1) Solid H‐PRF: 700 *g* × 8 min (5702 Eppendorf Centrifuge, Germany) (2) Liquid H‐PRF: 700 *g* × 8 min (5702 Eppendorf Centrifuge, Germany)	Cell migration: Transwell assay Tissue reaction and healing: Histology Histomorphometry	It was shown that H‐PRF + DBBM (H‐PRF bone block) can enhance early immune cell infiltration, leading to accelerated neovascularization and faster bone metabolism in vivo following maxillary sinus grafting with DBBM
*Dental implant*							
Di Summa et al.^85^ (2020)	To investigate if the growth factors, that cause the most robust gene expression changes, adsorb to titanium and collagen membranes	In vitro study	Human	(1) H‐PRF	(1) H‐PRF: 400 *g* (1570 rpm) × 12 min (Z 306 Hermle Universal Centrifuge, Germany)	Cell differentiation: ALP staining Gene and protein expression: Proteomic analysis Microarray analysis RT‐PCR Immunoassay Western blot Immunofluorescence staining	It was demonstrated that H‐PRF‐derived TGF‐β activity binds to titanium implants and collagen membranes, as evidenced by changes in gene expression and immunoassay results
*Treatment of the palatogingival groove*							
Durai et al.^86^ (2019)	To provide treatment strategies for PGG which include eradication of microbes, sealing the PGG to eliminate bacterial colonization and regenerating the attachment apparatus	Case report	Human	(1) MTA + Xenograft + H‐PRF Membrane	(1) H‐PRF: 3000 rpm × 10 min (REMI R‐8C Centrifuge, NR)	Tissue healing and regeneration: Clinical examination Radiography	The treatment outcomes achieved in this case include a clinical attachment gain of 8 mm, no increase in gingival recession, and the disappearance of the periapical radiolucency
Johns et al.^87^ (2014)	To eliminate the PGG and to regenerate the attachment apparatus	Case report	Human	(1) Biodentine + Bone Graft + H‐PRF Membrane	(1) H‐PRF: 3000 rpm × 10 min (REMI R‐8C Centrifuge, NR)	Tissue healing and regeneration: Clinical examination Radiography	This treatment modality led to attachment gain, reduction in pocket depth, and bone deposition in the osseous defect
Sakkir et al.^88^ (2018)	To describe the combined endodontic and surgical management of a complex type of PGG in a maxillary right lateral incisor with two canals	Case report	Human	(1) MTA + Bone Graft + H‐PRF Membrane	(1) H‐PRF: 3000 rpm × 10 min (REMI R‐8C Centrifuge, NR)	Tissue healing and regeneration: Clinical examination Radiography	H‐PRF promoted the regeneration of the attachment apparatus, demonstrating its cost‐effectiveness and regenerative potential
*Treatment of external root resorption and radicular cysts*							
Shivashankar et al.^89^ (2013)	To add knowledge to the existing literature regarding the combined use of graft material (H‐PRF and HAp) and barrier membrane in the treatment of large periapical lesions	Case report	Human	(1) MTA+ H‐PRF + HAp + H‐PRF Membrane	(1) H‐PRF: ≈ 400 *g* (3000 rpm) × 10 min (REMI R‐8C Centrifuge, NR)	Tissue healing and regeneration: Clinical examination Radiography	Clinical examination showed uneventful wound healing. Radiologically, the HAp crystals were completely replaced by new bone after 2 years
Mohammed Sadique et al.^90^ (2016)	To describe the healing of a defect which was treated using H‐PRF alone	Case report	Human	(1) MTA + H‐PRF	(1) H‐PRF: 3000 rpm × 10 min (REMI R‐8C Centrifuge, NR)	Tissue healing and regeneration: Clinical examination Radiography	Clinical examination showed uneventful wound healing. Radiologically, the defect was almost entirely replaced by new bone after 8 months
Manjushree and Prasad^91^ (2021)	To present the management of a lateral incisor and a dilacerated maxillary central incisor associated with a radicular cyst and external root resorption using CBCT	Case report	Human	(1) MTA + H‐PRF	(1) H‐PRF: 3000 rpm × 12 min (REMI R‐8C Centrifuge, NR)	Tissue healing and regeneration: Radiography	At the 3‐month, 6‐month, and 1‐year recall visits, healing was observed, as indicated by a significant reduction in the size of the peri‐radicular radiolucency
Teja et al.^92^ (2021)	To prospectively observe and analyze the 3‐year healing outcomes of surgically managed external root resorption using autologous H‐PRF membrane alone without any bone graft as a scaffold along with the Biodentine as a matrix	Case report	Human	(1) Biodentine + H‐PRF	(1) H‐PRF: 3000 rpm × 10 min (REMI R‐8C Centrifuge, NR)	Tissue healing and regeneration: Radiography	At the 3‐year follow‐up, postoperative CBCT showed satisfactory healing, with a noticeable reduction in lesion size and a thick radiopaque seal at the middle third, indicating repair of the resorptive site
Johri et al.^93^ (2022)	To evaluate the amniotic membrane's potential on bone healing post endodontic surgery and compare it with H‐PRF using color doppler sonography	Clinical study	Human	(1) H‐PRF Membrane + HAp bone graft (2) Amniotic Membrane + HAp bone graft	(1) H‐PRF: 3000 rpm × 10 min (REMI R‐8C Centrifuge, NR)	Tissue healing and regeneration: Sonography	The results showed that the amniotic membrane significantly enhanced angiogenesis at 1 month and led to a greater reduction in lesion surface area at 6 months, compared to H‐PRF. However, both biomaterials displayed similar osteogenic potential
*Bone healing after tooth hemisection*							
Gupta et al.^94^ (2020)	To examine the use of hemisection combined with H‐PRF with severely carious first and second mandibular molars and furcation involvement	Case report	Human	(1) H‐PRF	(1) H‐PRF: 3000 rpm × 10 min (REMI R‐8C Centrifuge, NR)	Tissue healing and regeneration: Clinical examination Radiography	Radiographs at the 6th and 12th months showed minimal resorptive changes, with better healing, occlusion, and function observed clinically
*Tooth auto transplantation*							
Chaudhary et al.^95^ (2015)	To expedite bone regeneration and provide a favorable environment for a transplanted maxillary central incisor	Case report	Human	(1) H‐PRF + Allograft + H‐PRF Membrane	(1) H‐PRF: ≈ 400 *g* (3000 rpm) × 10 min (REMI R‐8C Centrifuge, NR)	Tissue healing and regeneration: Clinical examination Radiography	A 1‐year follow‐up revealed no signs of bone loss, root resorption, or ankylosis, confirming the promising results of combining allograft with H‐PRF products
*Regenerative endodontic treatment*							
Shivashankar et al.^96^ (2012)	To investigate the usage of H‐PRF in revitalization of a necrotic and open apex tooth	Case report	Human	(1) H‐PRF + MTA	(1) H‐PRF: 3000 rpm × 10 min (REMI R‐8C Centrifuge, NR)	Tissue healing and regeneration: Clinical examination Radiography	At the 1‐year follow‐up, the tooth was asymptomatic, with positive pulp test responses. Radiographs showed root lengthening, thickened dentinal walls, regression of the periapical lesion, and apical closure
Johns et al.^97^ (2014)	To describe a new proposal for pulp revascularization with disinfection of pulp canal spaces using a unique combination of a photosensitizer solution and low‐power laser light	Case report	Human	(1) Photodynamic Therapy + H‐PRF + MTA	(1) H‐PRF: 3000 rpm × 10 min (REMI R‐8C Centrifuge, NR)	Tissue healing and regeneration: Clinical examination Radiography	Clinical examination showed no sensitivity to percussion or palpation. Radiographs revealed thickening of the dentinal walls, root lengthening, regression of the periapical lesion, and complete apical closure at 10 months. However, the teeth were not responsive to the electric pulp test
Faizuddin et al.^98^ (2015)	To investigate the usage of H‐PRF for the revitalization of immature nonvital tooth	Case report	Human	(1) H‐PRF + MTA	(1) H‐PRF: 3000 rpm × 10 min (REMI R‐8C Centrifuge, NR)	Tissue healing and regeneration: Clinical examination Radiography	At the 3‐, 6‐, 9‐, 12‐, and 14‐month follow‐ups, the patient remained asymptomatic, with no sensitivity to percussion or palpation. Radiographs showed regression of the periapical lesion and the initiation of root‐end closure
Yadav et al.^99^ (2015)	To highlight the non‐surgical management of symptomatic revascularization‐attempted tooth with immature apex using H‐PRF membrane matrix and MTA to promote periapical healing	Case report	Human	(1) H‐PRF + MTA	(1) H‐PRF: 400 *g* × 10 min (REMI R‐8C Centrifuge, NR)	Tissue healing and regeneration: Clinical examination Radiography	The 6‐month and 2‐year follow‐ups showed a reduction in periapical radiolucency and an adequately functional tooth
Prasanthi et al.^100^ (2018)	To describe the management of two pulpally involved carious human adult permanent molars with established symptomatic irreversible pulpitis	Case series	Human	(1) H‐PRF + Biodentine	(1) H‐PRF: ≈ 400 *g* (3000 rpm) × 10 min (REMI R‐8C Centrifuge, NR)	Tissue healing and regeneration: Clinical examination Radiography	At the 6‐, 12‐, and 24‐month recall, both teeth responded positively to pulp sensibility tests, and radiographic examination showed a normal periodontal ligament space
*Drug delivery*							
Monika et al.^101^ (2023)	To inspect the effect of i‐PRF and C‐PRF with and without metronidazole incorporation in connection with the rise of periodontal ligament fibroblast cell proliferation in vitro	In vitro study	Human	(1) i‐PRF (2) i‐PRF with Metronidazole (3) C‐PRF (4) C‐PRF with Metronidazole (5) Control Group	(1) i‐PRF with/without Metronidazole: 300 *g* × 5 min (2) C‐PRF with/without Metronidazole: 2000 *g* × 8 min	Cell proliferation: MTT assay	It was suggested that i‐PRF and C‐PRF with metronidazole incorporation can enhance cell proliferation more than i‐PRF and C‐PRF alone
*Corneal regeneration*							
Baadsgaard Bruun et al.^102^ (2023)	To describe the effect of autologous H‐PRF membrane for corneal reconstruction surgery in dogs	Case series	Animal (Dog)	(1) H‐PRF	(1) H‐PRF: 700 *g* × 8 min (Bio‐PRF Centrifuge, USA)	Tissue healing and regeneration: Clinical examination	H‐PRF membranes were effective as a graft material for corneal ulceration reconstruction surgery. The mean healing time for all dogs was 9 ± 5.5 days. At the final long‐term follow‐up, conducted 288 ± 44 days post‐surgery, minimal scarring, corneal pigmentation, and vascularization were noted
*Skin and hair regeneration*							
Shashank and Bhusha^103^ (2021)	To demonstrate the efficacy of i‐PRF in treating various dermatological conditions	Case series	Human	(1) i‐PRF	(1) i‐PRF: 800 rpm × 4 min (Remi R‐4C Centrifuge, NR)	Tissue healing and regeneration: Clinical examination	The study highlighted the effectiveness of i‐PRF in managing androgenetic alopecia, rejuvenating the under‐eye area, temporarily correcting facial skin folds, and promoting the healing of difficult wounds and ulcers
Mohamamad et al.^104^ (2022)	To study the effect of PRP and i‐PRF in patients suffering from androgenetic alopecia	Clinical study	Human	(1) PRP (2) i‐PRF	(1) i‐PRF: 700 rpm × 4 min (Remi Centrifuge, NR)	Hair growth: Hair density	Hair density increased by 18% at 3 months after applying PRP, and by 24% at 3 months after applying i‐PRF. This increase was maintained 6 months after treatment, with a mean of 185.53 ± 68.20 hairs/cm^2^ in the PRP group and 198.53 ± 68.20 hairs/cm^2^ in the i‐PRF group
*Myringoplasty*							
Sharma et al.^105^ (2018)	To find the efficacy of autologous H‐PRF during myringoplasty in the closure of tympanic membrane perforation by subjecting the patient to otoscopic examination and pure tone audiometry	Clinical study	Human	(1) Myringoplasty (2) Myringoplasty + H‐PRF	(1) H‐PRF: 2700 rpm × 12 min (NR)	Closure of tympanic perforation: Endoscopy Hearing assessment: Audiometry	H‐PRF improved the overall success rate of myringoplasty and led to a significant improvement in the air‐bone gap at 500 Hz, 1000 Hz, and 2000 Hz frequencies
Riaz et al.^106^ (2021)	To determine the efficacy of the usage of topical H‐PRF in improving outcomes of myringoplasty regarding graft uptake and hearing improvement	Clinical study	Human	(1) Myringoplasty (2) Myringoplasty + H‐PRF	(1) H‐PRF: 2300 rpm × 7 min (Cells Horizon Centrifuge, USA)	Graft uptake: Endoscopy Presence of post‐operative infection: Endoscopy Hearing assessment: Audiometry	After a 3‐month follow‐up, graft uptake was reported at 78% in the cases and 52% in the controls. The mean hearing improvement was 18 dB in the cases and 6 dB in the controls. Postoperative infection occurred in 8% of the cases and 32% of the controls
*Further optimization of H‐PRF*							
Wei et al.^107^ (2024)	To modify the inner wall surface of the PRF tubes with plasma gas to increase hydrophilicity and create an anaerobic environment inside the blood collection tubes, and then evaluate the size, mechanical properties, biological properties, and platelet activation of the H‐PRF clots prepared by super‐hydrophilic anaerobic plasmatrix tubes (SHAP tubes)	In vitro study	Human	(1) H‐PRF	(1) H‐PRF: 700 *g* × 8 min (Plasmatrident Centrifuge, China)	PRF size Mechanical properties: Tensile strength test Degradation rate: Degradation test Structural analysis: Scanning electron microscopy Platelet activation: Flow cytometry Antibacterial activity: Inhibition ring test Cell proliferation CCK‐8 assay Protein expression: Immunofluorescence staining Mineralization: ALP activity test ALP and alizarin red S staining	The SHAP tubes exhibited improved platelet activation properties, resulting in better mechanical strength, a longer degradation period, and enhanced regenerative properties in oral cell types, including gingival fibroblasts and alveolar osteoblasts
Wei et al.^108^ (2022)	To evaluate the effect of resting and compression time after centrifugation on the physical properties of H‐PRF membranes, and to provide optimal guidance regarding the clinical preparation of H‐PRF	In vitro study	Human	(1) Liquid H‐PRF	(1) Liquid H‐PRF: 700 *g* × 8 min (Plasmatrident Centrifuge, China)	PRF size and weight Mechanical properties Tensile strength test Structural analysis: Scanning electron microscopy	The findings demonstrated that H‐PRF membranes reached their maximum weight, volume, and mechanical properties after resting for 3–5 min in the tube post‐centrifugation, followed by a compression time of 120 s
Zheng et al.^109^ (2022)	To discover and confirm the optimum temperature for heat treatment before obtaining H‐PRF gels by investigating their structure, mechanical properties, and bioactivity of the H‐PRF gels after heating treatment	In vitro study	Human	(1) Alb‐PRF (2) Liquid H‐PRF	(1) H‐PRF Gel: 700 *g* × 8 min + PPP (heating for 10 min at 45°C or 60°C or 75°C or 90°C) (Bio‐PRF, USA) (2) Liquid H‐PRF: 700 *g* × 8 min (Bio‐PRF, USA)	PRF weight, and solidification time Degradation rate: Degradation test Structural analysis: Scanning electron microscopy Rheologic properties: Stress‐controlled rheometer Cell viability: Live/Dead assay	It was demonstrated that the H‐PRF gel obtained at 75°C for 10 min produced a uniform, moldable gel with a short solidification time, excellent rheological behavior, and a high percentage of live cells in the H‐PRF gel
Wu et al.^110^ (2023)	To investigate the effects of heating on the biological and mechanical characteristics of H‐PRF and explore the optimum heating temperature for H‐PRF thermal treatment	In vitro study	Human	(1) H‐PRF	(1) H‐PRF: 700 *g* × 8 min + No heating or heating at 50°C or 75°C or 90°C or 105°C for 10 s both side (Plasmatrident Centrifuge, China)	Structural analysis: Scanning electron microscopy Degradation rate: Degradation test Mechanical properties: Tensile strength test Cell viability and proliferation: CCK‐8 assay	Excessive heating beyond 90°C significantly prolonged the degradation of H‐PRF membranes and enhanced their mass stress. However, treatment at 105°C reduced cell activity beneath the membranes by over 50%, drastically decreasing their biological effects on human osteoblasts
Jagati et al.^111^ (2019)	To prepare H‐PRF matrix without using any compression device over a scaffold of collagen sheet and to evaluate its efficacy in chronic nonhealing ulcers	Clinical study	Human	(1) H‐PRF (With Compression) (2) H‐PRF + Collagen (Without Compression)	(1) H‐PRF: 2200‐3000 × 3 min (NR)	Wound healing: Photography	H‐PRF prepared using this new technique overcame the limitations of the compression method and showed comparable efficacy. The results from comparisons in weeks 0, 3, and 6 were statistically significant

Abbreviations: Alb‐PRF, albumin gel with liquid platelet‐rich fibrin; ALG, alginate; ALP, alkaline phosphatase; BMP, bone morphogenic protein; β‐TCP, beta tricalcium phosphate; CBCT, cone beam computed tomography; CCK‐8, cell counting kit‐8; CFU, colony‐forming unit; CHA, combined hyaluronic acid; CH, chitosan; C‐PRF: concentrated platelet‐rich fibrin; dB: decibel; DBBM: deproteinized bovine bone mineral; DFDBA: demineralized freeze‐dried bone allograft; DPSCs: dental pulp stem cells; EdU assay: 5‐ethynyl 2′‐deoxyuridine assay; ELISA, enzyme‐linked immunosorbent assay; GEL, gelatin; GAG, glycosaminoglycan; HA, hyaluronic acid; HAp, hydroxyapatite; H&E, hematoxylin and eosin; H‐PRF, horizontal platelet‐rich fibrin; i‐PRF, injectable platelet‐rich fibrin; L‐PRF, leukocyte platelet‐rich fibrin; MDP 99mTc, technetium‐99m methylene diphosphonate; MTA, mineral trioxide aggregate; mRNA, messenger ribonucleic acid; ncHAp, nanostructured carbonated hydroxyapatite; nHAp, nanostructured hydroxyapatite; NR, not reported; NRS, numerical rating scale; OB, osteoblasts; PDGF, platelet‐derived growth factor; PGG, palatogingival groove; PPP, platelet‐poor plasma; PRF, platelet rich‐fibrin; RNA, ribonucleic acid; RPM, revolution per minute; RT‐PCR, reverse transcription polymerase chain reaction; SHAP tubes, super‐hydrophilic anaerobic plasmatrix tubes; SVF, stromal vascular fraction; TGF, transforming growth factor; TRAP, tartrate‐resistant acid phosphatase; WOMAC, Western Ontario and McMaster Universities Osteoarthritis Index.

#### 3.2.1 Centrifugation protocols

**FIGURE 10:**
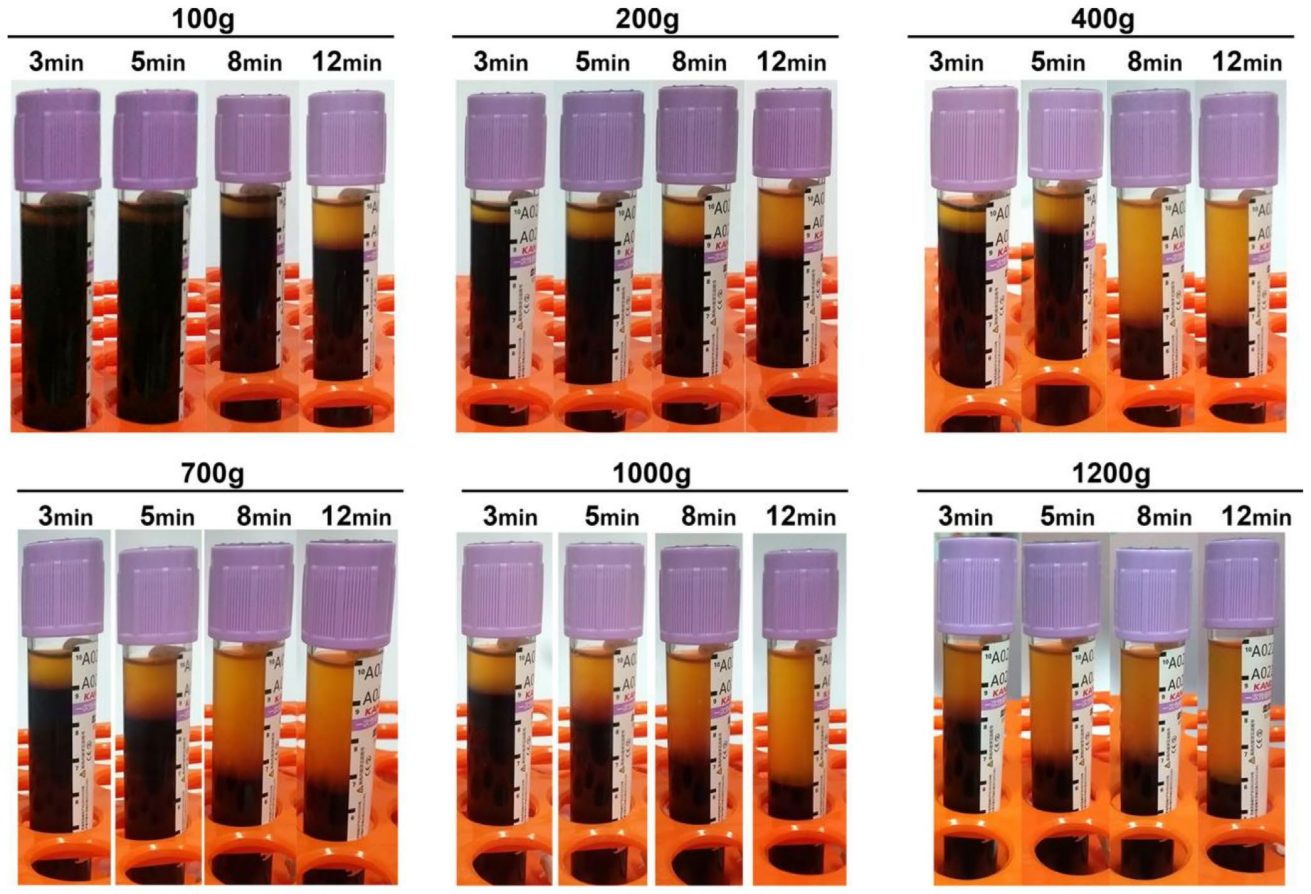
Clinical image demonstrating the plasma layer separation for the 24 protocols investigated in this study. Note that while some protocols reveal roughly identical plasma layer separation, the underlying cellular content in the various protocols may be drastically different. Reprinted from Miron et al.^38^

Three studies investigated centrifugation protocols for H‐PRF.^38^, ^51^, ^52^ Following initial studies that demonstrated that horizontal centrifugation produced more concentrated cells when compared to fixed‐angle, an additional study aimed to evaluate a wide range of protocols to determine the most optimized settings.^38^ In that study, 24 protocols were utilized systematically with varying speeds/RCF (100, 200, 400, 700, 1000, and 1200 *g*) and times (3, 5, 8, and 12 min), to better understand cell separation (Figure 10). As in previous studies, 1 mL blood layers were sequentially pipetted from the upper‐most layer downward until the entire 10 mL was collected. The samples were then analyzed via CBC to accurately quantify cell numbers within each distinct blood layer.^38^ The findings showed that: (1) the platelets accumulated more readily in the upper four layers than leukocytes due to their lower cellular density; (2) the centrifugation time had a greater influence on cell layer separation than speed; (3) the protocols exceeding 8 min typically resulted in more cells accumulating within the buffy coat layer rather than being evenly distributed throughout the entirety of the PRF clot; (4) the protocols at or below 200 *g* were ineffective at accumulating platelets or leukocytes; (5) the optimal centrifugation speed and time for solid PRF ranged between 400 and 700 *g* for 8 min, with 700 *g* for 8 min yielding the highest yield of platelets and leukocytes, which were better evenly distributed throughout the upper layers of the PRF; (6) the optimum centrifugation speed and time for liquid PRF ranged between 200 and 400 *g* for 5 min, resulting in the highest production of platelets and leukocytes (albeit less volume).^38^

**FIGURE 11:**
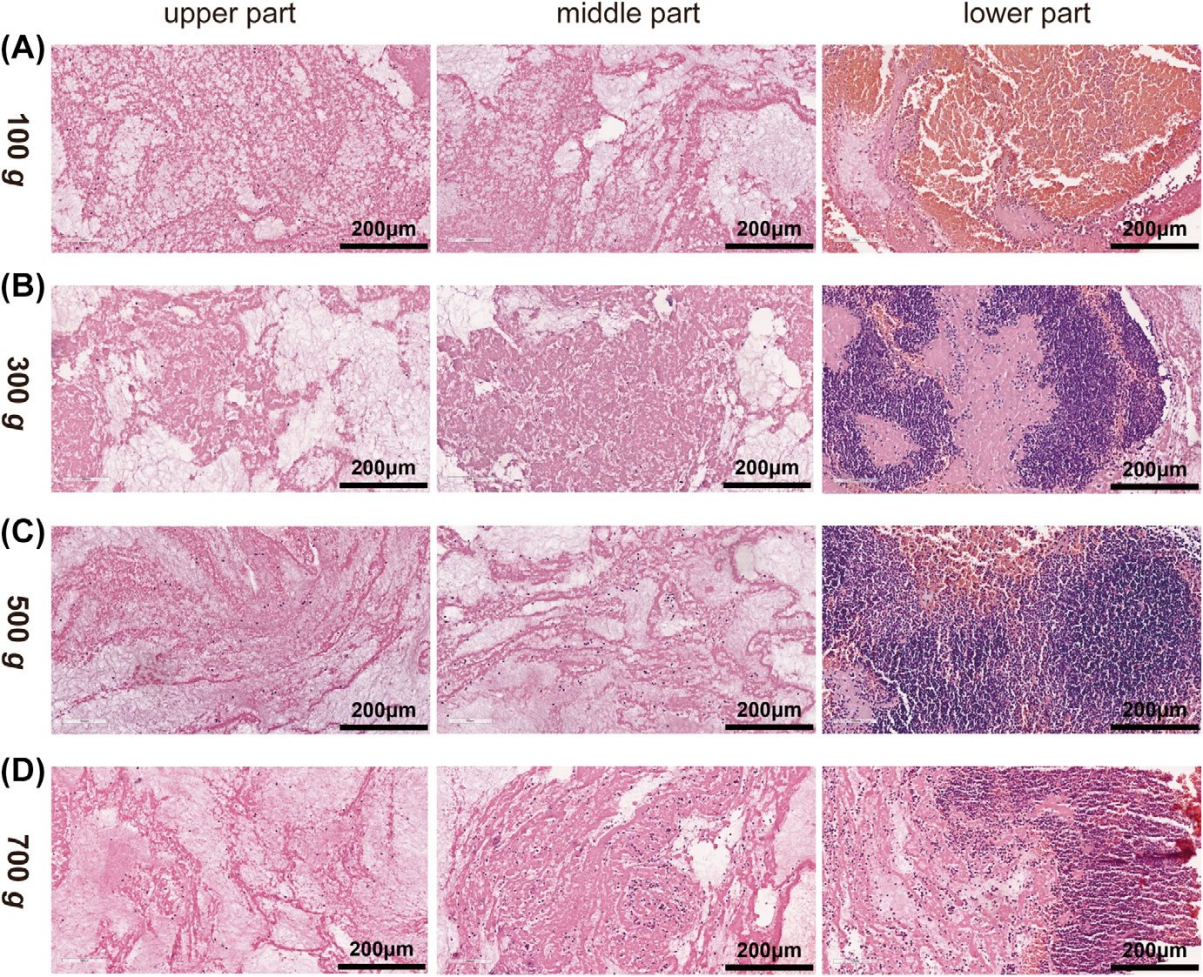
H&E staining observation of the three parts of liquid H‐PRF gels sectioned. (A, B, D) Most of the cells remained in the lower red blood cell portion at the centrifuge forces of 100 *g* and 300 *g*. (C) Cells obtained by the centrifugation protocol of 500 *g* were most evenly distributed in liquid H‐PRF (samples were performed in one). Reprinted with permission from Feng et al.^51^

Additionally, Feng et al also sought to determine the optimal centrifugation protocol for preparing liquid H‐PRF.^51^ In this study, they used horizontal centrifugation with RCF values of 100 *g*, 300 *g*, 500 *g*, and 700 *g* for 8 min, which had previously been established as the optimal centrifugation time for preparation.^51^ They found that as RCF values increased, both the volume and weight of liquid H‐PRF increased proportionally. SEM images and rheologic tests also demonstrated that higher RCF values resulted in a denser fibrin network and enhanced mechanical properties. CBC analysis indicated that the 500 *g* group had the highest total counts of leukocytes and neutrophils, while the 100 *g* group showed the greatest concentration of leukocytes and platelets. Furthermore, the histological analysis suggested that cells processed at 500 *g* for 8 min were the most uniformly distributed within the liquid H‐PRF (Figure 11).^51^

Lastly, the third study further evaluated the impact of the original (2700 rpm for 12 min) and modified (adjusted to ~400 *g* for 12 min) centrifugation protocols on the PRF matrices, using two commercially available horizontal centrifuges (Remi 8C® and Remi C854®), with assessments conducted at 20, 40, and 60 min of resting time.^52^ The modified protocol resulted in higher concentrations of VEGF and EGF compared to the original protocol in both devices. Additionally, platelet concentrations were higher with the modified protocol than with the original protocol across both centrifuge devices by the end of the second and third resting periods. The findings suggested that centrifuge type and RCF optimization are critical for achieving H‐PRF with improved cell viability and growth factor release.^52^

#### 3.2.2 Concentrated platelet‐rich fibrin (C‐PRF)

**FIGURE 12:**
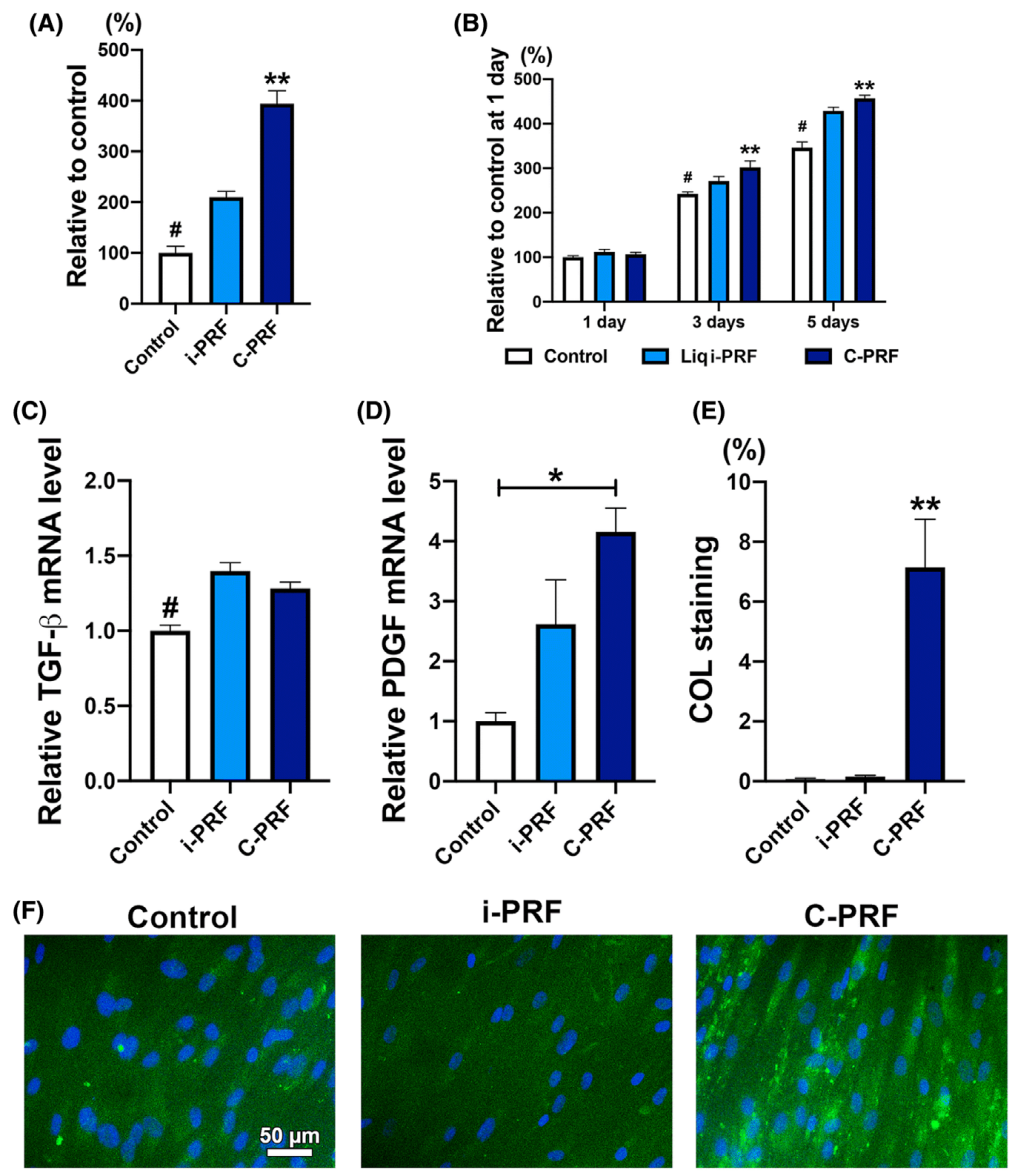
(A) Cell migration at 24 h and (B) cell proliferation at 1, 3, and 5 days in HGF‐1 cells. (C, D) Real‐time PCR analysis of the mRNA levels of (C) TGF‐β and (D) PDGF in human gingival fibroblasts treated with i‐PRF and C‐PRF at 3 days. (E) Quantitative and (F) representative staining of collagen I at 14 days (data represents means ± SE, * indicates significantly higher than the control group (*p* < 0.05), ** indicates significantly higher than all other groups (*p* < 0.05), # indicates significantly lower than all groups (*p* < 0.05)). Reprinted with permission from Fujioka‐Kobayashi et al.^53^

**FIGURE 13:**
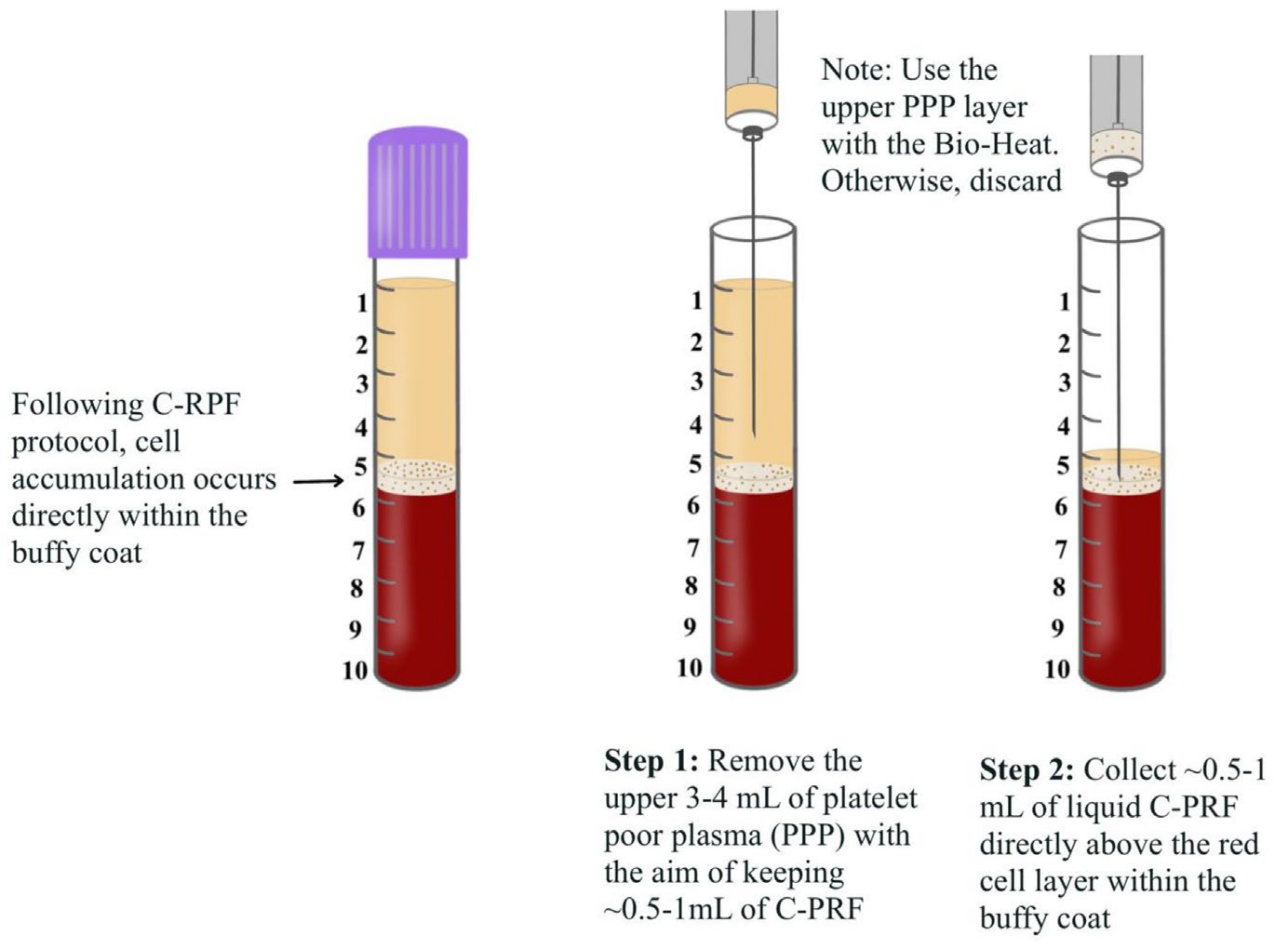
Methods to collect and concentrate C‐PRF. Following centrifugation at higher speeds (2000 *g* for 8 min), the majority of cells are located directly at the buffy coat layer. Instead of attempting to remove this layer using a long needle through the deep layers, it is highly advised to first remove the upper 4 mL layer of PPP, followed by collection of the C‐PRF buffy coat layer. Reprinted from Miron et al.^32^

This section reviews several studies that investigated the properties of the buffy coat layer and its upper PPP layer, prepared using horizontal centrifugation in different contexts.^53^, ^54^, ^55^ In a first study, Fujioka‐Kobayashi et al. compared the upper 1‐mL layer obtained through standard i‐PRF protocols at low centrifugation speeds (300 *g* for 5 min on a horizontal centrifuge) with 1 mL of PRF collected from the buffy coat layer using a high centrifugation protocol (3000 *g* for 8 min on a horizontal centrifuge) to specifically concentrate the cells within the platelet/leukocyte‐rich buffy coat layer.^53^ The results revealed a significant increase in growth factor release at the higher RCF protocol with the PRF taken from the buffy coat across all time points examined, particularly for PDGF‐AA, TGF‐β1, and EGF, which showed the highest levels compared to standard liquid‐PRF. Additionally, the higher RCF buffy coat protocol significantly enhanced fibroblast migration and proliferation compared to the control tissue culture plastic group (Figure 12).^53^ Owing to this discovery, this novel protocol and method to produce a more concentrated version of liquid‐PRF using higher RCF values with horizontal centrifugation was termed concentrated PRF (C‐PRF) which has since been clinically recommended and utilized (Figure 13).

Additionally, a recent study by Kargarpour et al. evaluated the distribution of fibrinogen in liquid H‐PRF prepared at three different RCFs (300, 700, and 2000) for 8 min.^55^ The fibrinogen concentration was found to be higher in the PPP compared to the buffy coat fraction of liquid H‐PRF, and it further decreased in the red fraction. When examining clottable H‐PRF fractions, the PPP and buffy coat fractions were found to contain 10.2% and 25.3% clottable matrix, respectively. Since more than half of the weight of the clottable buffy coat was attributed to cellular components, the results indicated that PPP is the main source of fibrinogen, while the buffy coat is primarily the source of cells.^55^

Kargarpour et al. also examined TGF‐β activity in the H‐PRF membranes—either in total or separated into the PPP and buffy coat fractions.^54^ They prepared the membranes using horizontal centrifugation at 210 *g*, 650 *g*, and 1500 *g* for 12 min, followed by repeated freeze‐thawing to prepare lysates for analysis. They found that the PPP exhibited significantly less TGF‐β activity than the buffy coat fraction at both high‐speed protocols (650 *g* and 1500 *g*). They concluded that TGF‐β activity in PRF lysates produced via horizontal centrifugation followed a gradient, with increasing concentration from the PPP to the buffy coat layer,^54^ likely due to the higher cellularity found within the buffy coat layer. Furthermore, it was noted by Kargarpour et al. that simply a fractionated clot demonstrated a significant ability to regenerate tissues.^66^ These findings may inspire future research to learn more about the biological properties of the unfractionated clot to further our understanding of the natural healing process, which may contribute to further optimization and understanding of PRF.

#### 3.2.3 Albumin gel with liquid platelet‐rich fibrin (Alb‐PRF)

Several studies have also been conducted to characterize Alb‐PRF (a mixture of heated PPP and C‐PRF) and to evaluate its efficacy in various contexts using the horizontal centrifugation method.^56^, ^57^, ^58^, ^59^ For instance, Fujioka‐Kobayashi et al. recently characterized the biological properties of Alb‐PRF, showing that the final mixture exhibited an even cell distribution while being capable of a slow and gradual release of growth factors (particularly TGF‐β1 and PDGF‐AA/AB) and significantly higher fibroblast proliferation at 5 days when compared to the control group.^56^ Additionally, Alb‐PRF induced significantly increased messenger ribonucleic acid (mRNA) expression of TGF‐β at 3 and 7 days and collagen type 1a2 at 7 days.^56^ Zeng et al. also demonstrated the potential of Alb‐PRF in cartilage tissue engineering.^59^ The results revealed that Alb‐PRF possesses a porous structure with a sustained release of growth factors, effectively supporting the proliferation, migration, adhesion, phenotype maintenance, and extracellular matrix secretion of rabbit chondrocytes. In vivo, Alb‐PRF facilitated chondrogenesis, resulting in regenerative cartilage that closely resembled natural knee joint cartilage.^59^ To enhance the bone regenerative potential of Alb‐PRF, Lima Barbosa et al. also incorporated nanostructured carbonated hydroxyapatite microspheres (ncHAp) into Alb‐PRF.^58^ However, their findings revealed that adding ncHAp spheres diminished the biological activity of Alb‐PRF, adversely affecting its early impact on osteoblast behavior.^58^ In a recent case report, Alb‐PRF was also used instead of a free gingival graft (FGG) for vestibuloplasty to eliminate the need for harvesting tissue from a donor site and, consequently, reduce related complications in a 25‐year‐old patient.^57^ After nine weeks, an increase in vestibule depth and keratinized tissue width was observed, and the authors reported that the use of Alb‐PRF in vestibuloplasty facilitated predictable surgical outcomes without significant complications.^57^

#### 3.2.4 Growth factors and cytokines release

**FIGURE 14:**
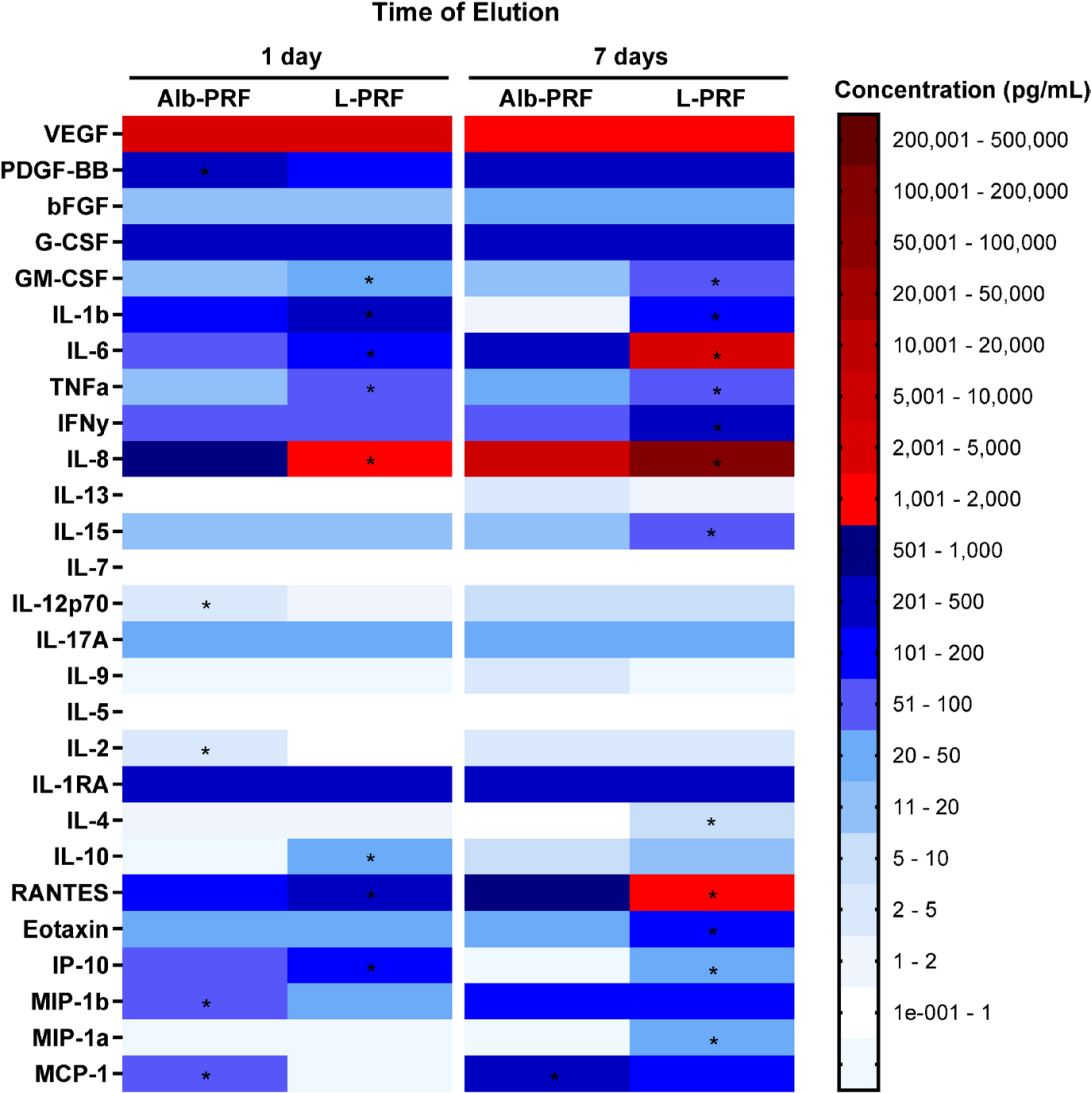
Heatmap of the variation of analyte concentrations in culture media from 1 to 21 days of incubation of fibrin membranes. Reprinted with permission from Lourenço et al.^60^

One study exclusively evaluated the release of growth factors and cytokines from PRF membranes produced via horizontal centrifugation.^60^ In this study, Lourenço et al. monitored the release of 27 growth factors and cytokines over three weeks using a multiparametric immunoassay.^60^ A substantial release of growth factors such as PDGF‐BB, FGF‐2, and VEGF was observed within the first 24 h, followed by a continual release of growth factors over a three‐week period.^60^ Both anti‐inflammatory and pro‐inflammatory cytokines displayed distinct release peaks, sustaining high elution rates for up to 21 days (Figure 14).^60^

#### 3.2.5 Anti‐inflammatory properties

Several studies have explored the anti‐inflammatory properties of PRF prepared using horizontal centrifugation.^61^, ^62^, ^63^, ^64^, ^65^, ^66^, ^67^ Nasirzadeh et al. evaluated the influence of H‐PRF on macrophage polarization, a key process during inflammation resolution.^63^ This study utilized murine primary macrophages and RAW 264.7 cells exposed to saliva and lipopolysaccharides (LPS) with and without PRF lysates or PRF‐conditioned medium.^63^ PRF lysates and conditioned medium reduced pro‐inflammatory markers such as IL‐1β and IL‐6, inhibited p65 nuclear translocation, and enhanced M2 macrophage markers like arginase‐1 and chitinase‐like 3, promoting a shift from the pro‐inflammatory M1 phenotype to the pro‐resolving M2 phenotype.^63^ Further research investigated the anti‐inflammatory effects of H‐PRF on mesenchymal cells.^65^ Lysates from liquid PPP, the buffy coat layer, and solid PRF were exposed to various mesenchymal cells alongside inflammatory cytokines. The results demonstrated that these PRF lysates significantly reduced the expression of pro‐inflammatory mediators such as IL‐6 and nitric oxide synthase (iNOS), as well as the phosphorylation and nuclear translocation of p65 in murine‐derived mesenchymal cells.^65^ However, these PRF lysates were not effective in reducing inflammation in human gingival fibroblasts or epithelial cells.^65^ Additionally, Kargarpour et al. further demonstrated that the fractions of liquid H‐PRF, including the buffy coat and PPP layers, were both rich in active TGF‐β; however, this activity was found to be heat‐sensitive.^62^

Another study also examined whether H‐PRF and its fractions could neutralize hydrogen peroxide, a key damage signal at sites of chronic inflammation.^61^ This study evaluated the ability of PRF, PPP, and the buffy coat to counteract hydrogen peroxide‐induced toxicity and oxidative stress in gingival fibroblasts.^61^ The results showed that lysates from PRF, PPP, and the buffy coat effectively neutralized hydrogen peroxide toxicity, while heated PPP lacked this effect. These findings underscored the potential of H‐PRF and its components in mitigating oxidative stress associated with chronic inflammatory conditions.^61^ Li et al. also examined the effects of liquid H‐PRF relative to hyaluronic acid (HA), a well‐established anti‐inflammatory agent commonly used in cartilage regeneration.^64^ Results showed that both liquid H‐PRF and HA treatments significantly suppressed the expression of catabolic mediators, such as ADAMTS‐5 and matrix metalloproteinases 13 (MMP‐13), whose inhibition could help prevent osteoarthritis progression. Additionally, both treatments reduced inflammation; mRNA expression levels of IL‐6, IL‐1β, Tumor necrosis factor alpha (TNF‐α), and cyclooxygenase 2 (COX‐2) were significantly downregulated. Notably, the 5% H‐PRF conditioned medium with a buffy coat demonstrated similar or slightly better effects on lowering inflammation when compared to HA.^64^

Although the anti‐inflammatory properties of H‐PRF have been evidenced using the studies mentioned above, the components mediating this effect remain unclear.^67^ In this regard, Kargarpour et al. investigated the role of the lipid fraction of H‐PRF.^67^ They demonstrated that lipids extracted from solid and liquid H‐PRF significantly reduced cytokine‐induced expression of IL‐6, CCL2, and CCL5 in bone marrow stromal cells, as well as LPS‐induced expression of IL‐1β, IL‐6, and CCL5 in primary bone marrow macrophages.^67^ The lipid fraction also lowered IL‐6 protein levels and inhibited the phosphorylation of key inflammatory signaling molecules, including p38, JNK, and NFκB‐p65, in bone marrow stromal cells.^67^ These findings suggest that the lipid fraction partially mediates the anti‐inflammatory properties of H‐PRF in vitro.^67^

The last study also compared H‐PRF membranes and unfractionated blood clots (UBC) in terms of their bioactivity and structural properties.^66^ Both lysates activated TGF‐β signaling in gingival fibroblasts and demonstrated anti‐inflammatory effects. However, while UBC lysates matched H‐PRF in bioactivity, their membranes lacked structural integrity, disintegrating rapidly caused by the erythrocytes.^66^ This underscores the necessity of centrifugation to produce durable H‐PRF membranes suitable for clinical applications.^66^

#### 3.2.6 Antibacterial properties

**FIGURE 15:**
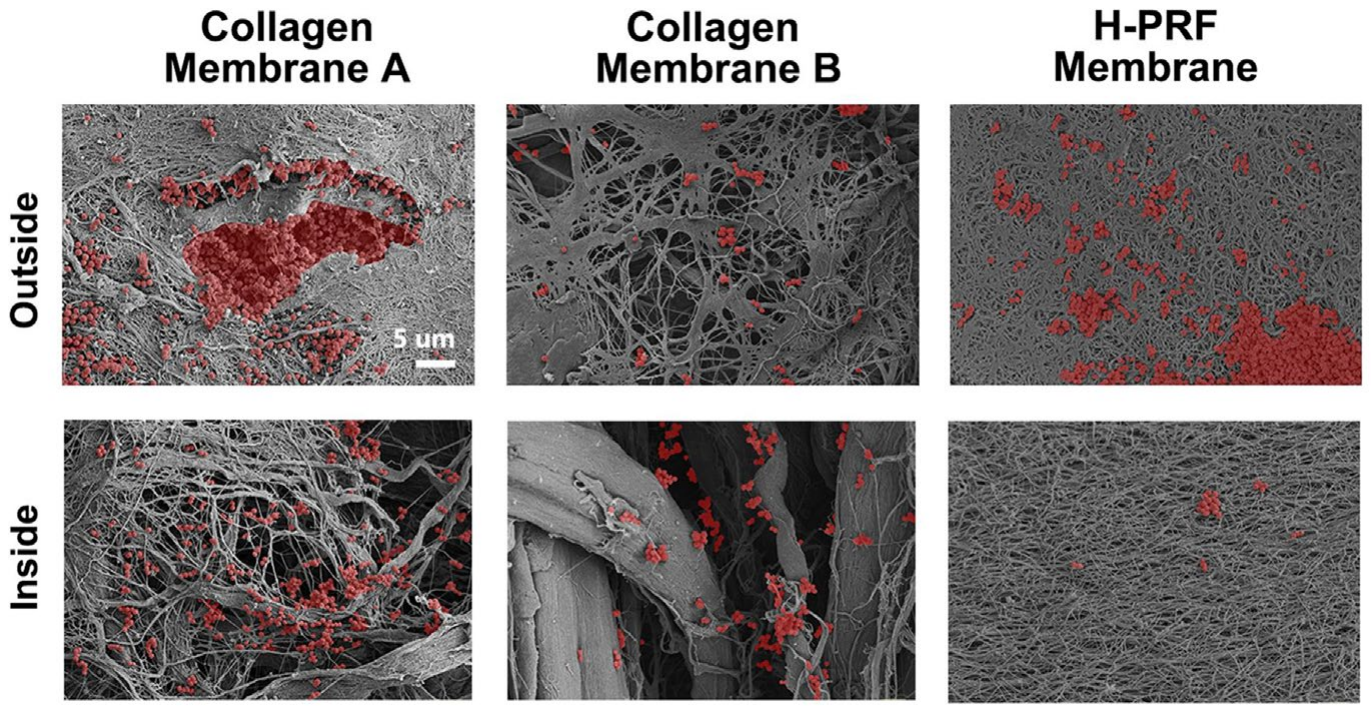
SEM images showing *S. aureus* penetration and the morphologies of 3 membranes at 48 h. *S. aureus* are labeled in red pseudo‐color. Reprinted with permission from Qiu et al.^68^

A recent study evaluated the barrier functionality of three widely used membranes, including H‐PRF, against bacterial invasion, comparing them to two commercially available resorbable collagen membranes.^68^ H‐PRF matrices were prepared using a centrifugation protocol of 700 *g* for 8 min, followed by compression into membranes. For the barrier function test, membranes were positioned between an inner and outer chamber, inoculated with *S. aureus*, and assessed for bacterial colony‐forming unit (CFU) counts and SEM analysis at 2, 24, and 48 h post‐inoculation.^68^ Results indicated that *S. aureus* minimally attached to or invaded either collagen membrane within the first 2 h but started degrading quickly thereafter, particularly on rougher collagen surfaces. Although H‐PRF showed higher CFU counts at 2 h, no significant bacterial penetration or degradation was observed in the H‐PRF membranes at 24 and 48 h. After 48 h, the collagen membranes showed considerable morphological changes due to bacterial presence, while H‐PRF membranes displayed minimal alterations (Figure 15).^68^ It was therefore suggested that H‐PRF membranes may be a useful addition in clinical surgeries where pre‐existing bacteria are present.

#### 3.2.7 Wound healing and soft tissue regeneration

**FIGURE 16:**
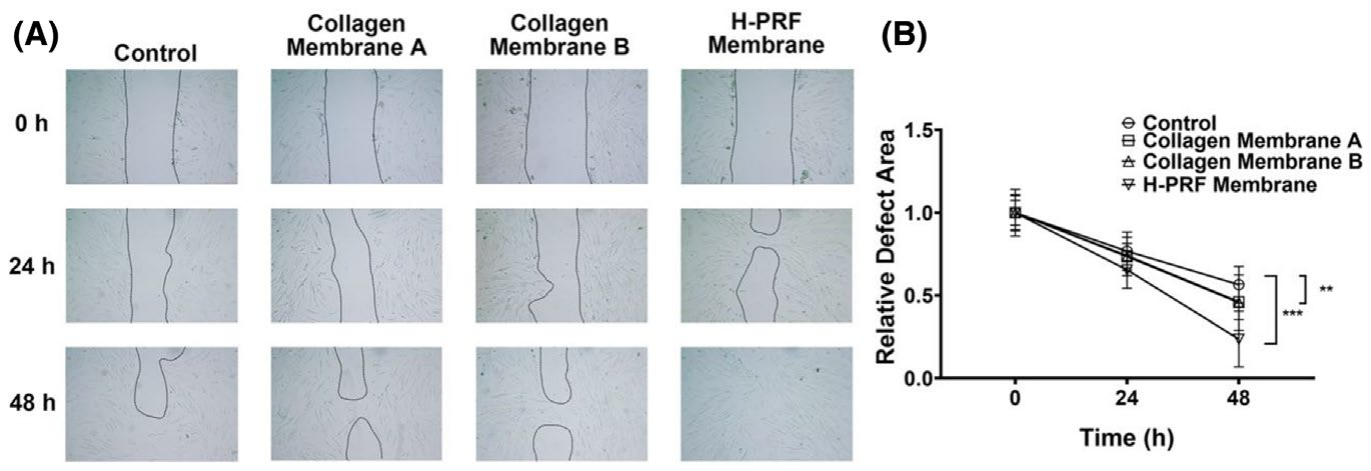
(A) Effects of leachates of three membranes on the migratory capacity of human gingival fibroblasts. Photos of scratch wound healing assay for HGF. (B) Quantitative analysis of the scratch wound healing assay. (**p < 0.01, and ***p < 0.001). Reprinted with permission from Qiu et al.^68^

Three studies evaluated the effects of H‐PRF on human gingival fibroblasts.^68^, ^69^, ^70^ In the first study, Qiu et al. compared the wound healing properties of solid H‐PRF against two commercially available resorbable collagen membranes.^68^ Through an in vitro scratch wound healing assay, they demonstrated that H‐PRF exhibited significantly faster wound closure rates than the two collagen membranes tested (Figure 16).^68^ In a second study, the effects of liquid H‐PRF, compound hyaluronic acid (CHA), and their 1:1 combination (CHA‐PRF) were also examined on human gingival fibroblasts for soft tissue regeneration.^69^ The results showed that the CHA‐PRF combination reduced the coagulation time of liquid H‐PRF and promoted the highest cell proliferation across all time points.^69^ The scratch wound healing and transwell assays also demonstrated that while liquid H‐PRF significantly enhanced cell migration compared to CHA alone, the CHA‐PRF group yielded the best overall results. Additionally, gene expression analysis of collagen type 1A1 and focal adhesion kinase (FAK) supported these findings, indicating that while liquid H‐PRF significantly increased the expression of these genes compared to CHA alone, CHA‐PRF demonstrated the highest expression levels.^69^

In a recent study, Imani et al. exposed gingival fibroblasts to lysates derived from H‐PRF membranes and H‐PRF serum and conducted bulk RNA sequencing to evaluate the activity retained in H‐PRF membranes versus H‐PRF serum.^70^ They identified that H‐PRF membrane lysates induced a more complex response in gingival fibroblasts compared to H‐PRF serum, though both enhanced chemokine expression.^70^

#### 3.2.8 Oral cancer treatment

A study examined the impact of lysates derived from solid H‐PRF membranes on inflammation in oral squamous carcinoma cell lines (HSC2 and TR146) and primary oral epithelial cells.^71^ In HSC2 cells, H‐PRF lysates significantly reduced chemokine expression (e.g., CXCL‐1, CXCL‐2, CXCL‐8, CXCL‐10, and CCL‐5) and attenuated p65 nuclear translocation induced by IL‐1β and TNF‐α. Although less pronounced, H‐PRF lysates also reduced IL‐1β‐ and TNF‐α‐induced chemokine expression in TR146 cells.^71^ In primary oral epithelial cells, however, H‐PRF lysates were found to elevate the basal expression of chemokines such as CXCL‐1, CXCL‐2, and CXCL‐8.^71^ This indicates that H‐PRF may have a biphasic effect, suppressing chemokine expression in oral squamous cell carcinoma lines while enhancing it in healthy oral epithelial cells.^71^

#### 3.2.9 Cartilage regeneration

Only one study to date, conducted by Li et al., explored the potential of H‐PRF matrices on cartilage regeneration.^64^ In this study, the effects of liquid H‐PRF were evaluated in comparison to HA, a common clinical treatment option for cartilage regeneration.^64^ Liquid H‐PRF was prepared at 500g for 8 min, and the effects of its conditioned medium—with and without the buffy coat—were compared to HA on chondrocyte activity. They reported that liquid H‐PRF had a notable impact on chondrocytes, influencing their proliferation and chondrogenic differentiation, with effects comparable to those in the HA group. The H‐PRF group also exhibited significantly higher expression of chondrogenic markers, including collagen type 2A1, compared to HA‐treated cells, while aggrecan expression was significantly greater in the HA group.^64^

#### 3.2.10 Orthopedic treatment

To date, five clinical studies (two retrospectives, two case reports, and one case series) have utilized H‐PRF as part of their treatment approach for orthopedic‐related conditions, demonstrating its potential in enhancing tissue healing and regeneration.^72^, ^73^, ^74^, ^75^, ^76^ In a retrospective clinical study, Ogéus evaluated the effect of H‐PRF matrices on the treatment of knee and hip osteoarthritis.^72^ He reported 2‐year follow‐up data from 104 patients with hip and knee osteoarthritis who received combined intra‐articular injections with PRF (C‐PRF and Alb‐PRF), prepared using horizontal centrifugation. The effectiveness of the treatment was assessed using the Western Ontario and McMaster Universities Arthritis Index (WOMAC) at 6 and 24 months, along with radiographic imaging at 6 months.^72^ Statistically significant improvements in WOMAC scores were observed for both hip and knee osteoarthritis. Additionally, radiographic images showed an increase in joint space, with a mean increase of 2 mm for knees and 1.6 mm for hip joints.^72^ In the other retrospective clinical study, Ogéus evaluated the effectiveness of H‐PRF matrices—a combination of i‐PRF, C‐PRF, and Alb‐PRF—in patients diagnosed with lateral epicondylitis, commonly known as tennis elbow.^73^ The analysis focused on numerical rating scale (NRS) scores from various clinical orthopedic tests and sonographic images taken before treatment, as well as at 1 month, 3 months, and 12 months post‐treatment.^73^ The findings revealed that H‐PRF matrices significantly reduced NRS pain scores and improved clinical orthopedic test outcomes. Additionally, the sonographic images showed notable improvements by 3 months post‐treatment. These results, combined with 1‐year follow‐up data, suggest significant long‐term benefits.^73^

There were also two published case reports as well as a case series on this topic. In the first case report, Ogéus treated a 13‐year‐old boy with a 3‐year history of severe Osgood–Schlatter disease, marked by significant ossification of the patellar tendon at the tuberositas tibiae.^74^ The treatment involved injections of C‐PRF and Alb‐PRF prepared using horizontal centrifugation. The patient reported significantly reduced pain levels 3 months post‐treatment and exhibited no palpation pain upon examination. Furthermore, the patient exhibited near‐complete remission of patellar tendon ossification after 2 months and was able to resume sports after a 3‐year hiatus.^74^ In the other case report, a 33‐year‐old professional boxer with a fractured humerus exhibiting delayed union and a partially torn supraspinatus tendon was treated with combined injections of C‐PRF and Alb‐PRF prepared using horizontal centrifugation.^75^ Within 1 month, he showed near‐complete recovery from both injuries and was able to return to professional boxing after 3 months.^75^ In the case series study, three patients with chronic pain and reduced physical function due to bone or cartilage degeneration were treated with a series of intra‐articular injections.^76^ Two of the patients received injections containing C‐PRF and Alb‐PRF prepared using horizontal centrifugation, SVF, and amniotic‐derived exosomes. Both patients experienced a significant reduction in pain and an improvement in physical function during the follow‐up period after the injections.^76^

#### 3.2.11 Bone regeneration

Several studies have explored the bone regenerative potential of PRF prepared using horizontal centrifugation in diverse contexts.^77^, ^78^, ^79^, ^80^, ^81^, ^82^ A recent in vivo study compared the osteoconductive properties of human and rat liquid C‐PRF prepared via horizontal centrifugation. In that study, Apaza Alccayhuaman et al. soaked resorbable collagen membranes in C‐PRF matrices before placing them over calvarial defects in rats.^77^ After three weeks, micro‐computed tomography (micro‐CT) and histological analyses were conducted.^77^ The findings revealed that rat PRF promoted new bone growth underneath the membrane and hybrid bone formation with collagen fibers embedded in the new bone, while human PRF lacked histological features of new bone formation in the center of the defect and showed minimal bone formation at the defect margins. Additionally, rat PRF resulted in significantly higher bone volume compared to human PRF.^77^

In another study, Anaya‐Sampayo et al. employed horizontal centrifugation and a lyophilizer to incorporate PRF into nano‐hydroxyapatite/chitosan/gelatin (nHAp‐CH‐GEL) scaffolds, with and without alginate, to enhance their biocompatibility.^81^ PRF‐supplemented scaffolds notably improved the viability of dental pulp stem cells (DPSCs) and osteoblasts derived from DPSCs (OB‐DPSCs).^81^ Among the groups tested, the nHAp‐CH‐GEL‐PRF scaffold demonstrated the most favorable physical and biological characteristics, highlighting lyophilized PRF's potential to enhance scaffold biocompatibility for bone tissue regeneration.^81^ A clinical study also compared the effectiveness of 70:30 nHAp and beta‐tricalcium phosphate (β‐TCP) combined with PRF to demineralized freeze‐dried bone allograft (DFDBA) with PRF in small maxillofacial osseous defects.^80^ Thirty patients were divided into two groups: Group A received nHAp + β‐TCP + PRF, and Group B received DFDBA + PRF. Postoperative evaluations showed no significant differences between the groups, with both demonstrating improvements in pain and swelling over time. A technetium‐99m methylene diphosphonate scan at 3 months also revealed similar graft integration in both groups. The study concluded that nHAp + β‐TCP + PRF offers comparable outcomes to DFDBA + PRF while being more cost‐effective and readily available.^80^

In addition to the aforementioned studies, solid H‐PRF has demonstrated the ability to inhibit osteoclastogenesis in vitro.^78^ A recent study investigated the effects of soluble extracts of H‐PRF membranes on osteoclast formation in murine bone marrow cultures.^78^ Osteoclastogenesis, induced by RANKL, macrophage colony‐stimulating factor (M‐CSF), and TGF‐β1, was significantly suppressed in the presence of PRF extracts, as evidenced by reduced tartrate‐resistant acid phosphatase (TRAP) staining and decreased pit formation.^78^ Gene expression analyses also showed downregulation of osteoclast markers, including TRAP, Cathepsin K, dendritic cell‐specific transmembrane protein (DCSTAMP), nuclear factor of activated T‐cells 1 (NFATc1), and osteoclast‐associated receptor (OSCAR).^78^ In addition to the previous study,^78^ the effects of liquid H‐PRF were also investigated on inflammation and osteoclast formation.^79^ Liquid H‐PRF, consisting of PPP, a cell‐rich buffy coat, and a red clot layer, was tested for its ability to suppress inflammatory responses and osteoclastogenesis in vitro.^79^ The results showed that lysates from PPP and the buffy coat layer significantly reduced the expression of inflammatory markers, including IL‐6 and COX‐2, in macrophages exposed to inflammatory agonists. Additionally, lysates from PPP, the buffy coat, and the red clot layer inhibited osteoclastogenesis by decreasing the expression of osteoclast marker genes (TRAP and Cathepsin K) and reducing osteoclast formation.^79^ These findings suggest that both solid and liquid H‐PRF exhibit potent anti‐osteoclastogenic effects, offering potential benefits for promoting bone regeneration.^78^, ^79^

Another study also investigated whether solid H‐PRF can enhance bone morphogenetic protein 2 (BMP‐2) expression in mesenchymal cells during bone regeneration.^82^ Kargarpour et al. blocked TGF‐β receptor 1 kinase in gingival fibroblasts exposed to H‐PRF lysates and found a significant increase in BMP‐2 expression.^82^ Additionally, fractions of liquid H‐PRF, specifically PPP and the buffy coat layer, induced BMP‐2 expression in fibroblasts, while heated PPP did not. The study also demonstrated that H‐PRF lysates activated canonical BMP signaling by inducing nuclear translocation and phosphorylation of Smad1/5.^82^ These findings suggest that PRF could activate TGF‐β receptor 1 kinase, which in turn promotes BMP‐2 production in mesenchymal cells.^82^

#### 3.2.12 Sinus floor augmentation

**FIGURE 17:**
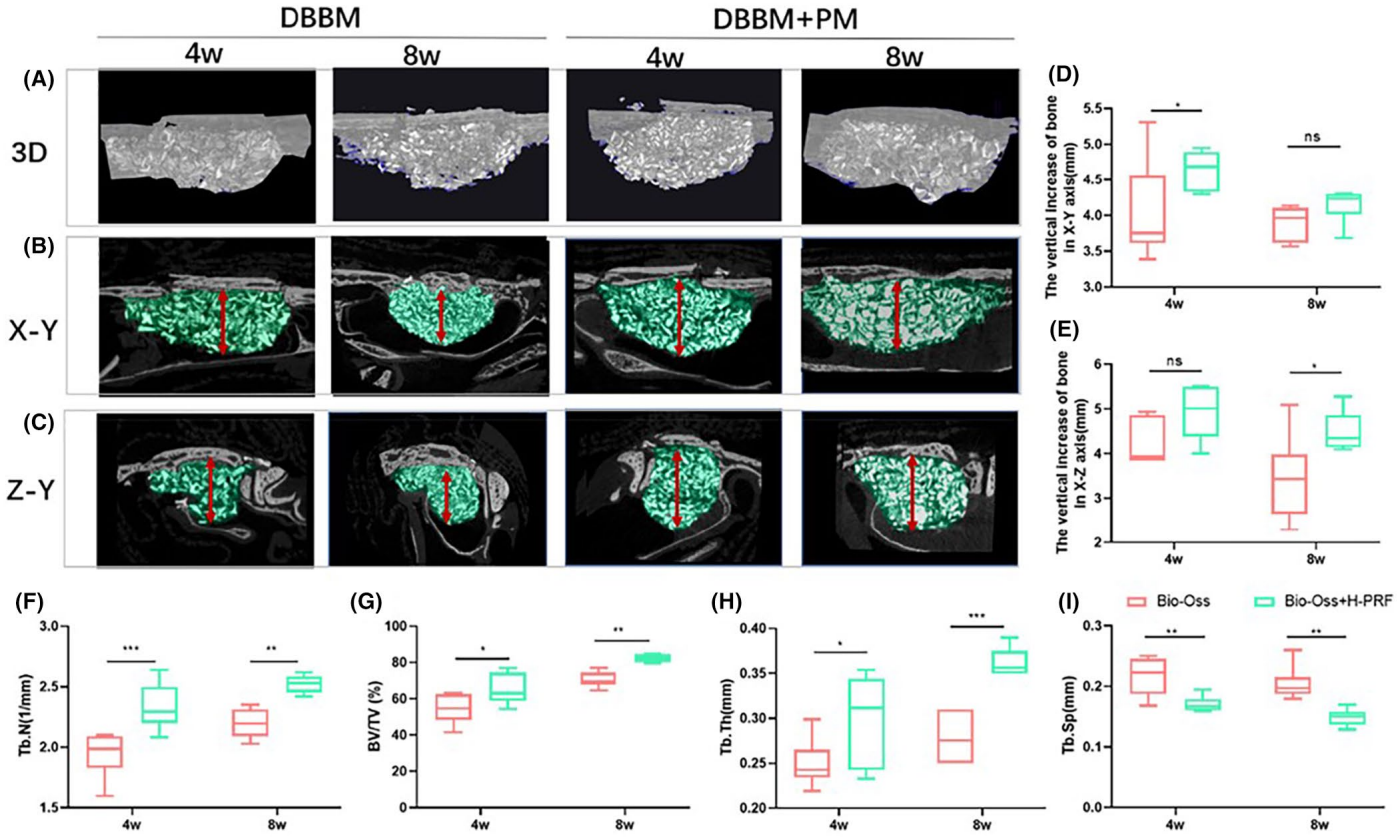
Micro‐CT views of augmented sinuses and statistical analysis of quantitative parameters. (A) 3D image of augmented sinuses. (B, C) The *X*–*Y* and *Z*–*Y* axis view of augmented sinuses separately. The red arrows show the measured distance. (D, E) the vertical increase of bone in the *X*–*Y* and *X*–*Z* axis separately (F) the volume of bone per unit volume of the total augmented volume (BV/TV, %), (G) trabecular thickness (Tb. Th, mm), (H) trabecular number (Tb. N, mm^−1^), and (I) trabecular separation (Tb. Sp, mm) within the augmented sinus (*n* = 6). *p < .05, **p < .01, and ***p < .001 (*n* = 6). Reprinted with permission from Yu et al.^83^

**FIGURE 18:**
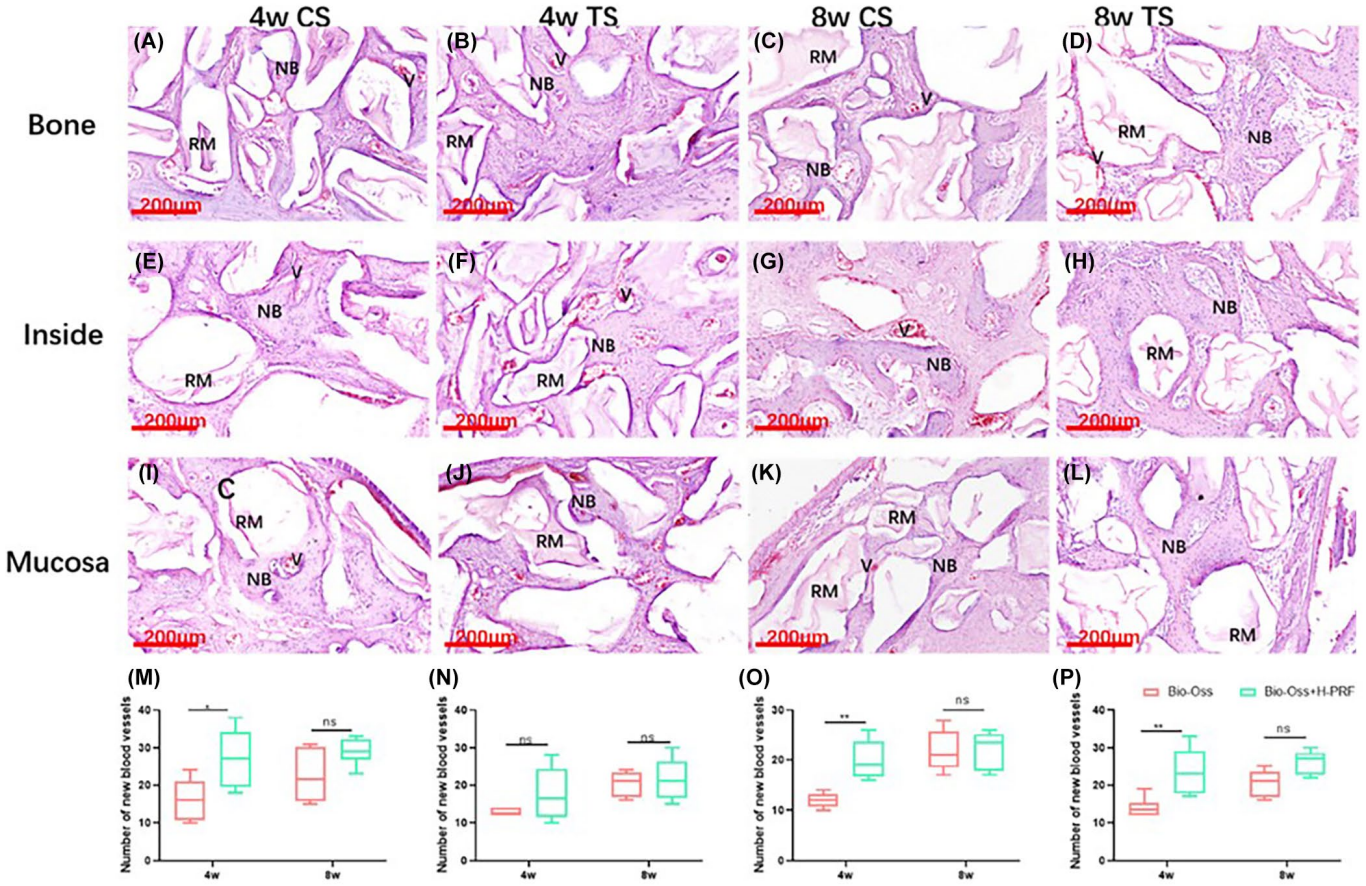
Photomicrograph of hematoxylin and eosin (H&E) staining showing the sinus augmentation region at 4 weeks (A, B, E, F, I, J) and 8 weeks (C, D, G, H, K, L). Three different areas were chosen in each group for comparison: Bone (close to the antrostomy); Inside (in the center of the elevated space); and Mucosa (subjacent to the sinus mucosa). B, DBBM residuum; NB, newly formed bone; rB, replaceable bone; V, vessel. (×20); Neovascularization of different areas (M) close to the antrostomy; (N) in the center of the elevated space; (O) subjacent to the sinus mucosa. (P) the total situation calculated by hematoxylin and eosin (H&E) staining at week 4 and week 8 (*n* = 6). ns: not statistically significant versus control group, *p < .05, **p < .01, and ***p < .001. Reprinted with permission from Yu et al.^83^

The effect of H‐PRF on sinus floor augmentation has also been evaluated in two studies, which compared the impact of deproteinized bovine bone mineral (DBBM) with and without H‐PRF in rabbits.^83^, ^84^ In both studies, H‐PRF was prepared at 700 *g* for 8 min and then mixed with 0.1 g of DBBM to create the H‐PRF bone blocks.^83^, ^84^ In the first study, Yu et al. performed micro‐CT and histological analyses after 4 and 8 weeks.^83^ They demonstrated that the H‐PRF bone block group showed significantly higher vertical bone gain in the sinus floor, with increased bone volume/total volume (BV/TV) percentage, trabecular thickness (Tb.Th), and trabecular number (Tb.N), along with lower trabecular separation (Tb.Sp) at both time points compared to the DBBM group (Figure 17). Additionally, a greater number of new blood vessels and more osteoclasts were observed in the H‐PRF bone block group than in the DBBM group at both time points, particularly in areas close to the bone plate. The H‐PRF bone block group also exhibited more new bone formation and less material residue at the 8‐week mark (Figure 18).^83^ In their second study, Yu et al. investigated the effects of DBBM and DBBM combined with H‐PRF (H‐PRF bone block) on healing and immune responses during sinus floor augmentation at early time points.^84^ They collected maxillary samples at 3‐, 7‐, and 14 days post‐surgery and conducted histological analyses.^84^ The findings revealed that the H‐PRF bone block group had a higher number of immune cells at 3‐ and 7 days post‐surgery compared to the DBBM‐only group, particularly in areas near the mucosal lining and bone plates. Moreover, at 14 days, the H‐PRF bone block group demonstrated a significantly greater number of new blood vessel formations and early signs of osteoclast development.^84^ Additionally, they examined the impact of H‐PRF bone blocks on the migration of osteoblasts and THP‐1 macrophages using a Transwell assay in vitro, confirming that the culture medium from H‐PRF bone blocks significantly enhanced the migration of both osteoblasts and THP‐1 macrophages.^84^

#### 3.2.13 Dental implant

Since PRF is commonly used in conjunction with dental implants and collagen membranes, Di Summa et al. conducted an interesting study to explore whether H‐PRF activity adheres to titanium surfaces and collagen membranes.^85^ They found that titanium surfaces and collagen membranes exposed to H‐PRF, followed by thorough washes with buffered saline, resulted in a significant upregulation of IL‐11, NOX‐4, and PRG‐4 gene expression in gingival fibroblasts. These findings suggest that TGF‐β derived from H‐PRF binds to biomaterials like titanium and collagen membranes.^85^ Furthermore, recognizing the frequent use of PRF as a coating for dental implants, the authors also examined whether the temperature during bone drilling affects H‐PRF activity.^85^ They stimulated gingival fibroblasts with heated (65°C and 95°C for 5 min) or unheated H‐PRF and found that heating H‐PRF at 56°C for 5 min did not alter the expression of target genes compared to control H‐PRF at room temperature. However, heating at 95°C for 5 min completely abolished TGF‐β activity. They suggested that H‐PRF‐derived TGF‐β activity is not significantly influenced by the surgical procedure itself.^85^

#### 3.2.14 Treatment of the palatogingival groove

Three case reports have examined the application of PRF prepared using horizontal centrifugation for the treatment of the palatogingival groove (PGG), a rare anatomical malformation that often leads to endodontic‐periodontal lesions.^86^, ^87^, ^88^ In one case, PGG in a lateral incisor was managed with root canal therapy, apicectomy, groove sealing with Biodentine, and the application of a bone graft combined with an H‐PRF membrane.^87^ At a 24‐month follow‐up, clinical outcomes showed improved attachment levels, reduced pocket depth, and significant bone deposition in the osseous defect.^87^ Another report described the management of a PGG in a maxillary lateral incisor with two canals, which resulted in combined periodontal and endodontic lesions.^88^ The treatment involved endodontic therapy, surgical intervention, and the application of bioactive materials, including H‐PRF. H‐PRF facilitated the regeneration of the attachment apparatus, showcasing its cost‐effective and regenerative potential.^88^ In the third case, a maxillary incisor with an endodontic‐periodontal lesion associated with PGG was treated using root conditioning with tetracycline, groove sealing with mineral trioxide aggregate (MTA), and the application of a bone graft combined with an H‐PRF membrane.^86^ Clinical and radiographic follow‐ups confirmed complete healing, demonstrating the importance of H‐PRF application in preventing tooth loss.^86^

#### 3.2.15 Treatment of external root resorption and radicular cysts

Several studies have also investigated the effect of PRF prepared using horizontal centrifugation for the treatment of external root resorption and radicular cysts in diverse contexts.^89^, ^90^, ^91^, ^92^, ^93^ A case report described the management of external root resorption and a large periapical lesion in a 28‐year‐old male with a history of trauma.^92^ The treatment involved root canal therapy followed by surgical curettage of the lesion. Biodentine and H‐PRF were used to seal the resorption site. A 3‐year follow‐up demonstrated successful healing, suggesting that the combination of Biodentine and H‐PRF can be an effective approach for treating external root resorption and associated periapical defects.^92^ Another case reported the management of a radicular cyst and external root resorption in a dilacerated maxillary central incisor in a 17‐year‐old boy.^91^ Treatment involved root canal therapy, cyst enucleation, apicoectomy, and placement of H‐PRF. At a 1‐year follow‐up, significant healing was observed, with a reduction in the size of the radiolucency, highlighting the utility of H‐PRF in the successful management of radicular cysts and associated root resorption.^91^ Similarly, another case involved a 26‐year‐old male with a large periapical lesion in the maxillary anterior region.^90^ Following root canal therapy, periapical surgery was performed, and the surgical defect was filled with H‐PRF. At an 8‐month follow‐up, clinical examination showed uneventful wound healing, and radiographs revealed substantial bone regeneration, further supporting the regenerative potential of H‐PRF in managing periapical lesions and enhancing tissue repair in endodontic surgery.^90^ The other case report also presented a periapical endodontic surgery performed on a 45‐year‐old male with swelling in the upper front teeth region and a large bony defect.^89^ The defect was filled with a combination of H‐PRF and HAp bone graft crystals, covered with an H‐PRF membrane. Clinical evaluation showed uneventful healing, and radiological follow‐up at 2 years confirmed the complete replacement of HAp crystals with new bone.^89^

In contrast, Johri et al., in a randomized clinical trial, compared the healing potential of H‐PRF with the amniotic membrane in endodontic surgery.^93^ Thirty‐four patients with periapical bony lesions underwent surgical curettage, followed by filling with HAp graft and coverage with either amniotic membrane or H‐PRF. The results demonstrated that the amniotic membrane significantly improved angiogenesis at 1 month and showed a greater reduction in lesion surface area at 6 months, compared to H‐PRF. However, both biomaterials exhibited similar osteogenic potential.^93^

#### 3.2.16 Bone healing after root hemisection

A case report examined the use of hemisection combined with PRF prepared through horizontal centrifugation in a 25‐year‐old male patient with severely carious first and second mandibular molars and furcation involvement. After endodontic treatment and hemisection, H‐PRF was placed in the extraction socket to prevent alveolar ridge resorption, and a fixed prosthesis was placed at 3 months. The patient was monitored for 12 months, with clinical and radiographic assessments at 1, 3, 6, and 12 months. Radiographs showed minimal resorption, with favorable healing, improved occlusion, and function, highlighting H‐PRF's effectiveness in preserving the socket and supporting healing after hemisection.^94^

#### 3.2.17 Tooth auto transplantation

In one case report, a 22‐year‐old male was diagnosed with a horizontally impacted left central incisor located just below the nasal floor, accompanied by an odontoma.^95^ The odontoma was extracted, and the central incisor was carefully removed from its socket without sectioning, with minimal bone removal. The resulting bony defect was filled with a mixture of allograft and an H‐PRF clot, and the incisor was replanted with the aid of splinting. In addition to the allograft‐PRF mixture, an H‐PRF membrane was used as an autologous barrier membrane to cover the defect. A one‐year follow‐up showed no signs of bone loss, root resorption, or ankylosis, confirming the promising results of combining allograft with H‐PRF products.^95^

#### 3.2.18 Regenerative endodontic treatments

Various studies have demonstrated the application of PRF prepared through horizontal centrifugation in regenerative endodontic treatments, including vital pulp and revitalization therapies.^96^, ^97^, ^98^, ^99^, ^100^ In a case series, two patients with symptomatic irreversible pulpitis in permanent mandibular molars were treated using vital pulp therapy.^100^ After pulpotomy and caries excavation, H‐PRF was applied as a medicament, followed by Biodentine pulp capping and composite resin restorations. At 6‐, 12‐, and 24‐month follow‐ups, both teeth showed positive pulp sensibility and normal periodontal ligament space in radiographs, highlighting the potential of H‐PRF in vital pulpotomy.^100^

For revitalization, a case report described a 9‐year‐old boy with a fractured immature maxillary central incisor that developed pulpal necrosis and apical periodontitis.^96^ After canal irrigation with sodium hypochlorite and chlorhexidine, triple antibiotic paste was placed during the initial phase. H‐PRF was then applied to the canal, followed by 3 mm of grey MTA. After 3 days, the tooth was sealed with glass ionomer and composite restoration. At the 1‐year follow‐up, the tooth was asymptomatic, with positive pulp test responses. Radiographs showed root lengthening, thickened dentinal walls, regression of the periapical lesion, and apical closure.^96^ Similarly, a 14‐year‐old boy with a traumatized upper right central incisor, featuring an immature root, open apex, and periapical radiolucency, was treated with the same procedure.^98^ At 3‐, 6‐, 9‐, 12‐, and 14‐month follow‐ups, the patient was asymptomatic, with no sensitivity to percussion or palpation, and radiographs revealed regression of the periapical lesion and initiation of root‐end closure.^98^

Another case report described a novel approach to pulp revascularization using photodynamic therapy for canal disinfection combined with H‐PRF for pulp revitalization.^97^ A 9‐year‐old boy with necrotic upper central incisors was treated with sodium hypochlorite irrigation, photodynamic therapy, and H‐PRF application, followed by gray MTA and permanent filling. At the 10‐month follow‐up, clinical examination showed no sensitivity to percussion or palpation. Radiographs revealed root lengthening, dentinal wall thickening, regression of the periapical lesion, and apical closure; however, the tooth was not responsive to electric pulp testing.^97^ Lastly, a failed revascularization case was managed with apexification using an H‐PRF membrane as an apical matrix barrier to stabilize MTA.^99^ After removing the old MTA and disinfecting the canal with calcium hydroxide, H‐PRF was placed at the root tip, followed by a 5‐mm MTA apical plug. The canal was subsequently obturated with thermoplasticized gutta‐percha. Follow‐ups at 6 months and 2 years showed periapical radiolucency reduction and functional tooth restoration. This case highlights one‐step apexification with H‐PRF and MTA as a predictable alternative for managing long‐term revascularization failures.^99^

#### 3.2.19 Drug delivery

One study also investigated the drug delivery capabilities of liquid PRF matrices produced using horizontal centrifugation.^101^ In this research, Monika et al. incorporated metronidazole, a cell proliferative agent, into C‐PRF (prepared at 2000 *g* for 8 min on a horizontal centrifuge) and i‐PRF (prepared at 300 *g* for 5 min on a horizontal centrifuge).^101^ They then evaluated the drug delivery effectiveness of these liquid PRF matrices in comparison to C‐PRF and i‐PRF alone. The results indicated that cell proliferation of fibroblasts was escalated by the addition of C‐PRF, i‐PRF metronidazole incorporation, and the C‐PRF metronidazole incorporation group, while the i‐PRF group showed a decreasing proliferation cell count.^101^

#### 3.2.20 Corneal regeneration

A single animal study has investigated the effectiveness of H‐PRF membranes for corneal regeneration in a case series involving dogs.^102^ In this study, H‐PRF membranes were prepared at 700 *g* for 8 min and used in corneal reconstruction surgery. Before the procedure, hematology and fibrinogen levels were analyzed in each dog to ensure H‐PRF membrane quality. The membranes were then applied to the corneal ulcer bed, with post‐surgical evaluations conducted at 5–7, 12–14, and 30–40 days, and followed by a long‐term assessment.^102^ Positive healing outcomes with a “good” quality PRF membrane were observed in six out of seven dogs. In one dog, fibrinogen levels were below the normal range, leading to a “poor” quality PRF membrane, and the dog developed a descemetocele 13 days post‐surgery, requiring additional surgical intervention. The average healing time for all dogs was 9 ± 5.5 days. Minimal scarring, pigmentation, and vascularization were noted at the long‐term follow‐up, conducted 288 ± 44 days after surgery.^102^

#### 3.2.21 Skin and hair regeneration

A recent case series by Shashank and Bhushan also demonstrated the efficacy of i‐PRF prepared via horizontal centrifugation in treating various dermatological conditions.^103^ The study highlighted its effectiveness in managing androgenetic alopecia, rejuvenating the under‐eye area, temporarily correcting facial skin folds, and promoting the healing of challenging wounds and ulcers.^103^ A comparative study also evaluated the effects of PRP and i‐PRF, both prepared using horizontal centrifugation, in treating androgenetic alopecia.^104^ Fifteen patients in each group received monthly treatments over 3 months. Hair density increased by 18% in the PRP group and 24% in the i‐PRF group at 3 months, with results sustained at 6 months (mean density: 185.53 ± 68.20 hairs/cm^2^ for PRP, 198.53 ± 68.20 hairs/cm^2^ for i‐PRF).^104^ Both treatments improved hair growth and reduced hair loss, but i‐PRF showed superior results.^104^

#### 3.2.22 Myringoplasty

Two prospective randomized clinical studies conducted by Riaz et al. and Sharma et al. evaluated the efficacy of topical H‐PRF in enhancing myringoplasty outcomes.^105^, ^106^ In the first study by Sharma et al., 100 patients underwent myringoplasty. In 50 patients, H‐PRF was placed over the graft and in the external auditory canal during the surgery, while the remaining 50 patients underwent the procedure without H‐PRF.^105^ The study demonstrated that H‐PRF improved the overall success rate of myringoplasty and resulted in a significant improvement in the air‐bone gap (ABG) at 500 Hz, 1000 Hz, and 2000 Hz frequencies.^105^ In the other study by Riaz et al., 50 participants underwent myringoplasty using the underlay technique, with 25 receiving H‐PRF drops and 25 serving as controls.^106^ After 3 months, graft uptake was higher in the H‐PRF group (78% vs. 52%), mean hearing improvement was significantly greater (18 dB vs. 6 dB), and postoperative infection rates were significantly lower (8% vs. 32%).^106^

#### 3.2.23 Further optimization of H‐PRF

Recent studies have aimed to enhance the therapeutic potential of H‐PRF by optimizing tube inner surface materials, adjusting resting and compression times post‐centrifugation, developing innovative techniques, and establishing suitable heating protocols to prolong the degradation of H‐PRF matrices.^107^, ^108^, ^109^, ^110^, ^111^ In this section, we discuss the current findings in the literature addressing these optimization efforts.

**FIGURE 19:**
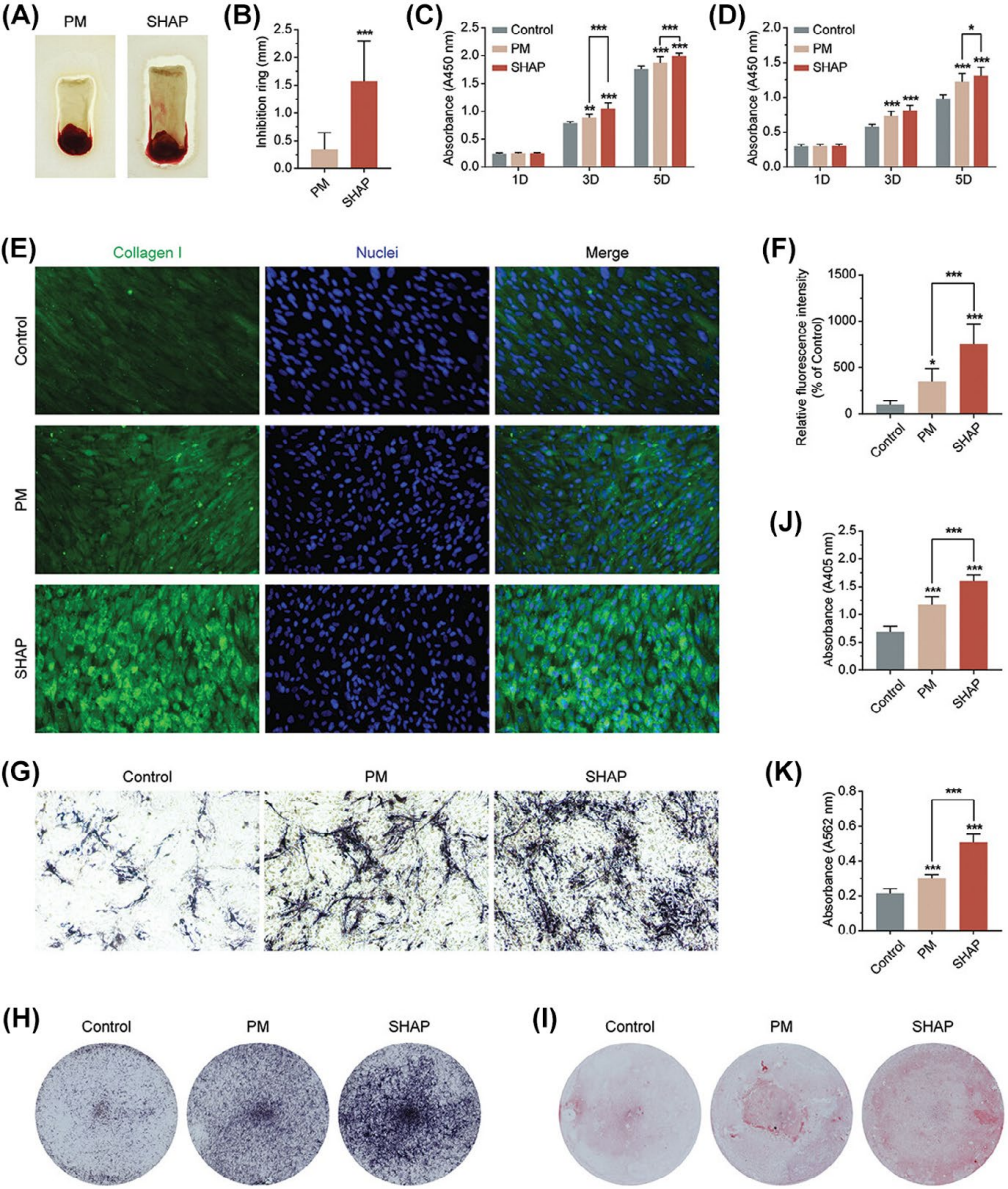
H‐PRF prepared by SHAP tubes has better antibacterial, collagen secretion, and mineralization promotion effects. (A) Comparison of the inhibition rings of H‐PRF membranes prepared by PM and SHAP tubes. (B) Quantification of the inhibition rings in (A). (C) CCK‐8 experiment to analyze the effect of H‐PRF prepared by PM and SHAP tubes on the proliferation of human gingival fibroblasts. (D) CCK‐8 experiment to analyze the effect of H‐PRF prepared by PM and SHAP tubes on the proliferation of human osteoblasts; (E) fluorescence staining of the effect of H‐PRF prepared by PM and SHAP tubes on collagen secretion of human gingival fibroblasts. (F) Quantification of fluorescence staining in (E). (G, H). ALP staining of human osteoblasts after 7 days of mineralization. (I) Alizarin red staining of human osteoblasts after 14 days of mineralization. (J) ALP activity analysis of human osteoblasts after 7 days of mineralization. (K) Quantification of staining in (I). (*n* = 6, ***p < 0.01 and ***p < 0.001). Reprinted with permission from Wei et al.^107^

For tube optimization, Wei et al. recently optimized tube surfaces by modifying the inner wall of glass tubes with gas plasma treatment (plasmatrix [PM] tubes) and adjusting the gas environment to create super‐hydrophilic anaerobic plasmatrix tubes (SHAP tubes).^107^ These tubes were designed to provide an anaerobic environment for H‐PRF preparation, enhancing both their mechanical strength and bioactivity. Characterization tests showed that this anaerobic setting stimulated platelet activation within the tubes.^107^ After compression, the resulting H‐PRF membrane exhibited a fibrous, cross‐linked network with high fracture strength, favorable degradation characteristics, and a notable increase in size. The H‐PRF membranes prepared using SHAP tubes significantly enhanced collagen synthesis in human gingival fibroblasts and osteoblast mineralization, while also demonstrating excellent biocompatibility and antibacterial properties (Figure 19).^107^

To provide optimal guidance on H‐PRF clinical preparation, Wei et al. assessed how varying resting (0, 1, 3, 5, 7, and 10 min) and compression (10, 30, 60, 90, 120, and 180 s) times post‐centrifugation affected the physical properties of H‐PRF membranes.^108^ Results showed that both the weight and volume of H‐PRF clots and membranes peaked at 3 min, followed by a decrease after 7 min of resting. The maximum strain of H‐PRF membranes was significantly reduced in the 10‐minute group compared to the 3‐ and 5‐minute groups, with maximum stress observed at 3 min, decreasing with extended resting time. SEM revealed that a compression time under 60 s resulted in a looser fibrous structure, whereas optimal internal fiber cross‐linking and maximum stress occurred with a 3‐min resting period followed by 120 s of compression.^108^

Regarding heating protocols for prolonging the degradation time of H‐PRF matrices, two studies have explored the optimal temperatures, each targeting either the liquid or solid form of H‐PRF.^109^, ^110^ For liquid H‐PRF, Zheng et al. investigated optimal heating temperatures to form H‐PRF gels with enhanced structural, mechanical, and biological properties.^110^ In this study, the 2‐mL upper layer of H‐PRF was collected and heated at temperatures of 45°C, 60°C, 75°C, and 90°C for 10 min before being combined with the 2‐mL lower layer.^110^ Results showed that H‐PRF gels heated at 75°C for 10 min solidified significantly faster (over tenfold increase compared to unheated controls) and had optimal degradation resistance, with over 90% cell viability. Additionally, findings indicated that higher heating temperatures increased gel density and enhanced mechanical properties compared to unheated controls.^110^ For solid H‐PRF, Wu et al. investigated the effects of heating on the biological and mechanical properties of H‐PRF membranes, aiming to identify the optimal temperature for thermal treatment.^109^ They applied a range of temperatures—room temperature (37°C), 50°C, 75°C, 90°C, and 105°C — for 10 s on both sides of H‐PRF membranes and showed that heating above 90°C significantly prolonged membrane degradation to 3–4 weeks and increased mass stress. However, at 105°C, cell viability within the H‐PRF membrane decreased by over 50%, significantly diminishing the biological effects on human osteoblasts.^109^

To eliminate the need for a compression device, a new approach for preparing H‐PRF membranes was developed using a collagen sheet scaffold.^111^ In this technique, PRF was prepared using a horizontal centrifuge and immediately transferred using a pipette onto a sterile, dry collagen sheet placed over a sheet of paraffin‐impregnated gauze in a petri dish. The setup was left undisturbed for approximately 20 min.^111^ To evaluate its efficacy, the membranes were tested on 15 patients with chronic non‐healing ulcers lasting over 3 months. The method demonstrated comparable efficacy to compression‐based techniques. Significant wound healing improvements were noted at 0, 3, and 6 weeks post‐op.^111^ The incorporation of a collagen sheet also enhanced wound healing, making this method simple, effective, and reliable.^111^

## 4 DISCUSSION

This review represents the first comprehensive review of studies on PRF prepared specifically using horizontal centrifugation. By systematically examining the unique properties and regenerative applications of H‐PRF, an in‐depth analysis of horizontal centrifugation outcomes was provided, including exploratory studies focused on further optimizing PRF matrices created through this approach. We also conducted critical comparisons with the more commonly used fixed‐angle centrifugation method. These comparisons enabled an evidence‐based assessment regarding which centrifugation technique better supported various biological attributes, including cellular concentration and distribution, fibrin network structure, growth factor and cytokine release, and overall regenerative potential, thereby offering valuable insights into the most effective PRF preparation strategies in regenerative medicine.^36^, ^37^, ^48^

In recent years, there has been a growing interest among researchers and clinicians in the use of horizontal centrifugation for the preparation of PRF matrices. This technique, which is routinely employed in advanced research laboratories and medical settings, offers superior separation of cellular layers and types based on their density compared to traditional fixed‐angle centrifugation devices, which are primarily designed for pelleting.^33^ This advantage has led to a series of studies comparing the effectiveness of horizontal centrifugation with fixed‐angle methods, aiming to assess the differences in biological properties and overall regenerative potential of the resulting PRF matrices.

**FIGURE 20:**
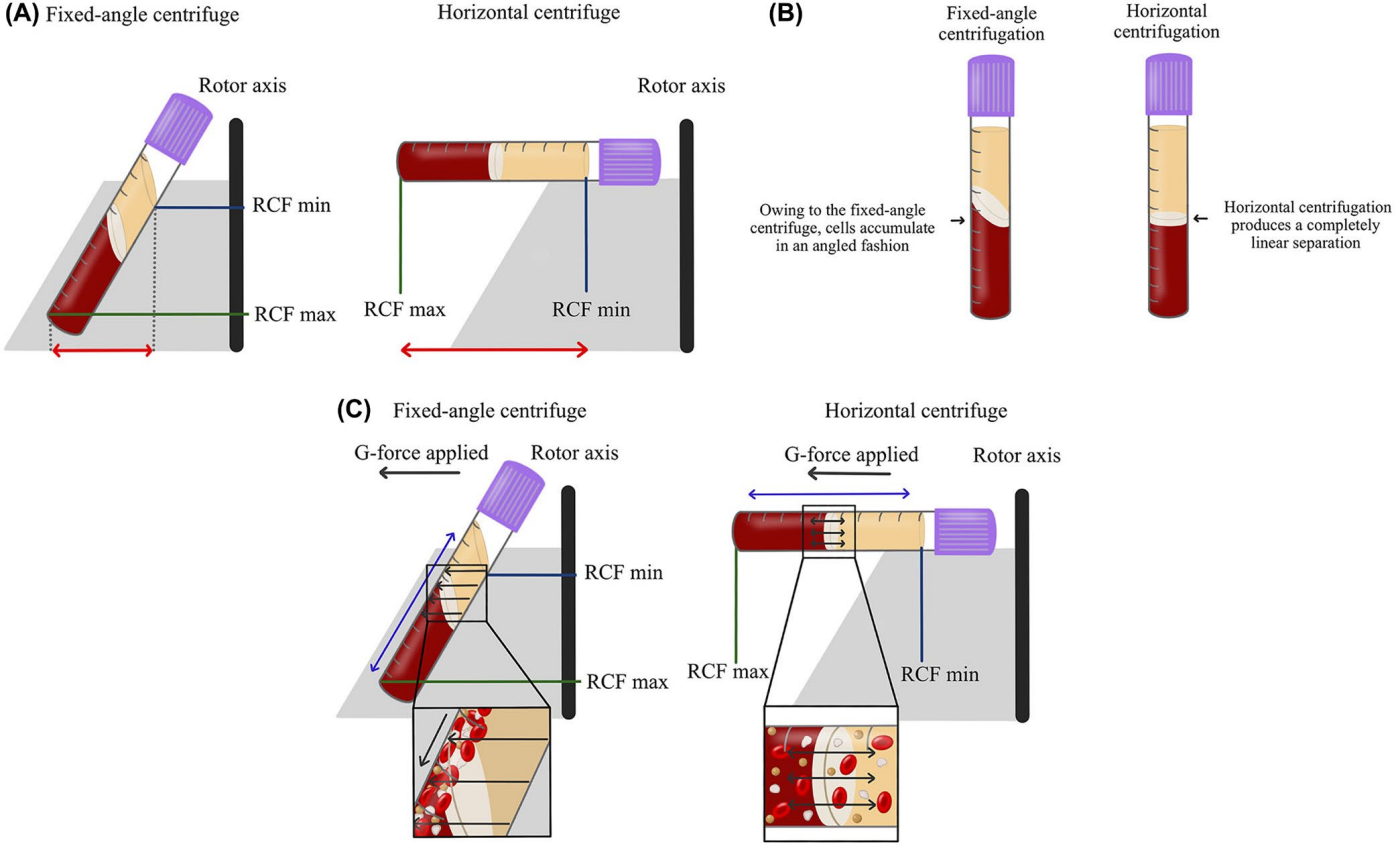
Illustrations comparing fixed‐angle and horizontal centrifuges. (A) With fixed‐angle centrifuges, a greater separation of blood layers based on density is achieved owing to the greater difference in RCF‐min and RCF‐max. (B) Following centrifugation on fixed‐angle centrifuges, blood layers do not separate evenly and as a result, an angled blood separation is observed. In contrast, horizontal centrifugation produces an even separation. (C) Owing to the large RCF values (~200–700 *g*), cells are pushed toward the outside and downward. On a fixed‐angle centrifuge, cells are pushed toward the back of centrifugation tubes and then downward/upward based on cell density. These g‐forces produce additional shear stress on cells as they separate based on density along the back walls of centrifugation tubes. In contrast, horizontal centrifugation allows for the free mobility of cells to separate into their appropriate layers based on density allowing for more optimal cell separation as well as less trauma/shear stress on cells. Reprinted with permission from Miron et al.^40^

Most of these comparative studies have previously focused on quantifying and analyzing the distribution of cells within the PRF matrices.^35^, ^36^, ^37^, ^40^, ^41^, ^42^, ^43^, ^45^, ^46^, ^47^, ^48^ The majority of studies have shown that horizontal centrifugation can significantly lead to greater concentrations of cells, particularly leukocytes, when compared to the PRF matrices produced using available fixed‐angle devices.^36^, ^40^, ^43^, ^46^ It has also been demonstrated that horizontal centrifugation allows for a more even distribution of cells compared to fixed‐angle centrifugation.^35^, ^41^, ^42^, ^46^, ^48^ In fixed‐angle centrifuges, cells are forced toward the back of the centrifugation tubes and then move either downward or upward according to their density. This process generates additional shear stress on the cells as they separate along the back walls of PRF tubes. Notably, cells are concentrated/clumped together with difficulty in properly separating (Figure 20).^35^ In contrast, horizontal centrifugation allows for unrestricted cell movement, facilitating their separation into distinct layers according to density. This results in enhanced cell separation and reduced trauma and shear stress on the cells.^35^ This improved separation is also attributed to the greater difference in RCF‐min and RCF‐max in horizontal centrifugation compared to the fixed‐angle method (Figure 20).^33^ It is also worth noting that the separation and accumulation of leukocytes within the PRF matrix are much more difficult than that of platelets. This is particularly true when using fixed‐angle centrifugation since the number of RBCs typically outnumbers white blood cells (WBCs) by about 1000:1. As a result, most cells tend to accumulate along the back distal surface of the PRF tubes, making accurate separation challenging.^40^

Since several studies have highlighted the superiority of horizontal centrifugation in the quantification and distribution of cells compared to fixed‐angle centrifugation methods,^35^, ^36^, ^40^, ^41^, ^42^, ^43^, ^46^, ^48^ researchers have begun to explore optimized centrifugation protocols for the preparation of solid and liquid H‐PRF. Miron et al. conducted a CBC analysis to evaluate 24 protocols by varying centrifugation forces (100, 200, 400, 700, 1000, and 1200 *g*) and times (3, 5, 8, and 12 min) for preparing both solid and liquid forms of H‐PRF.^38^ They concluded that horizontal centrifugation at 700 *g* for 8 min achieved optimal cell distribution and the highest cell concentration in solid H‐PRF matrices. Additionally, horizontal centrifugation at 200–400 RCF for 5 min was suggested as the most effective protocol for maximizing platelet and leukocyte concentrations in liquid H‐PRF matrices.^38^

In terms of growth factor and cytokine release, although there are findings that demonstrate horizontal centrifugation can release growth factors as well as anti‐ and pro‐inflammatory cytokines and chemokines relevant to tissue repair,^36^, ^37^, ^48^, ^60^, ^61^, ^63^, ^64^, ^65^, ^66^, ^67^ studies comparing horizontal and fixed‐angle centrifugation remain limited. While some findings suggest that horizontal centrifugation enhances the release of specific growth factors and cytokines in certain contexts compared to fixed‐angle centrifugation,^36^, ^37^, ^48^ one study reported no differences between the two methods.^45^ These discrepancies may stem from variations in study designs and the centrifugation protocols employed. Additionally, H‐PRF has demonstrated significantly better antibacterial activity compared to both L‐PRF produced by fixed‐angle centrifugation and commonly used resorbable collagen membranes.^43^, ^68^ This enhanced antibacterial activity has been attributed to the high concentration of immune cells present in the H‐PRF matrices.^43^

Regarding the comparative physical characteristics of PRF, such as size and weight, produced by horizontal and fixed‐angle centrifugation, it is important to note that the relevance of these characteristics does not pose much impact on final cell numbers as previously discussed. It is well known that a higher RCF protocol will lead to a larger PRF clot but also pushes the cells unevenly towards the buffy coat zone or the bottom of the PRF tube, especially as higher g‐forces and times are utilized. Furthermore, it is well agreed that the PRF tubes and their hydrophilic surfaces can have quite a significant impact on the final size of PRF clots, irrespective of the protocol utilized.^112^, ^113^

Recent findings also indicated that the low‐speed centrifugation concept (LSCC) that was proposed in the 2016–2018 era^114^, ^115^, ^116^, ^117^ is not the most effective method for increasing platelet and leukocyte concentrations in final liquid PRF matrices. Higher cell concentrations can be achieved by pushing cells specifically to the buffy coat region using higher RCF values.^118^ This method demonstrated an over 10‐fold increase in baseline platelet and leukocyte concentrations within the 0.3–0.5 mL buffy coat layer above the RBC corpuscle layer, compared to the 1.5‐ to 2.5‐fold increase achieved with standard i‐PRF produced on fixed‐angle centrifuges. The PRF obtained specifically from this harvesting technique was given the working name Concentrated‐PRF or C‐PRF.^118^ It should be noted that the initial study on C‐PRF did use fixed‐angle centrifugation;^118^ to validate these findings under horizontal centrifugation, Fujioka‐Kobayashi et al. compared C‐PRF with the standard i‐PRF protocol.^53^ Their findings showed that C‐PRF, obtained from the buffy coat layer using higher centrifugation protocols, led to a threefold increase in growth factor release, significantly enhancing gingival fibroblast migration, proliferation, gene expression, and COL I synthesis.^53^ Other findings have also confirmed higher concentrations of cells in the buffy coat layer (C‐PRF) compared to the upper layer (PPP).^55^ In addition, a study tracing TGF‐β activity found that the buffy coat layer exhibits higher TGF‐β activity than the PPP layer, further supporting the evidence of a greater cell presence in the C‐PRF layer.^54^

**FIGURE 21:**
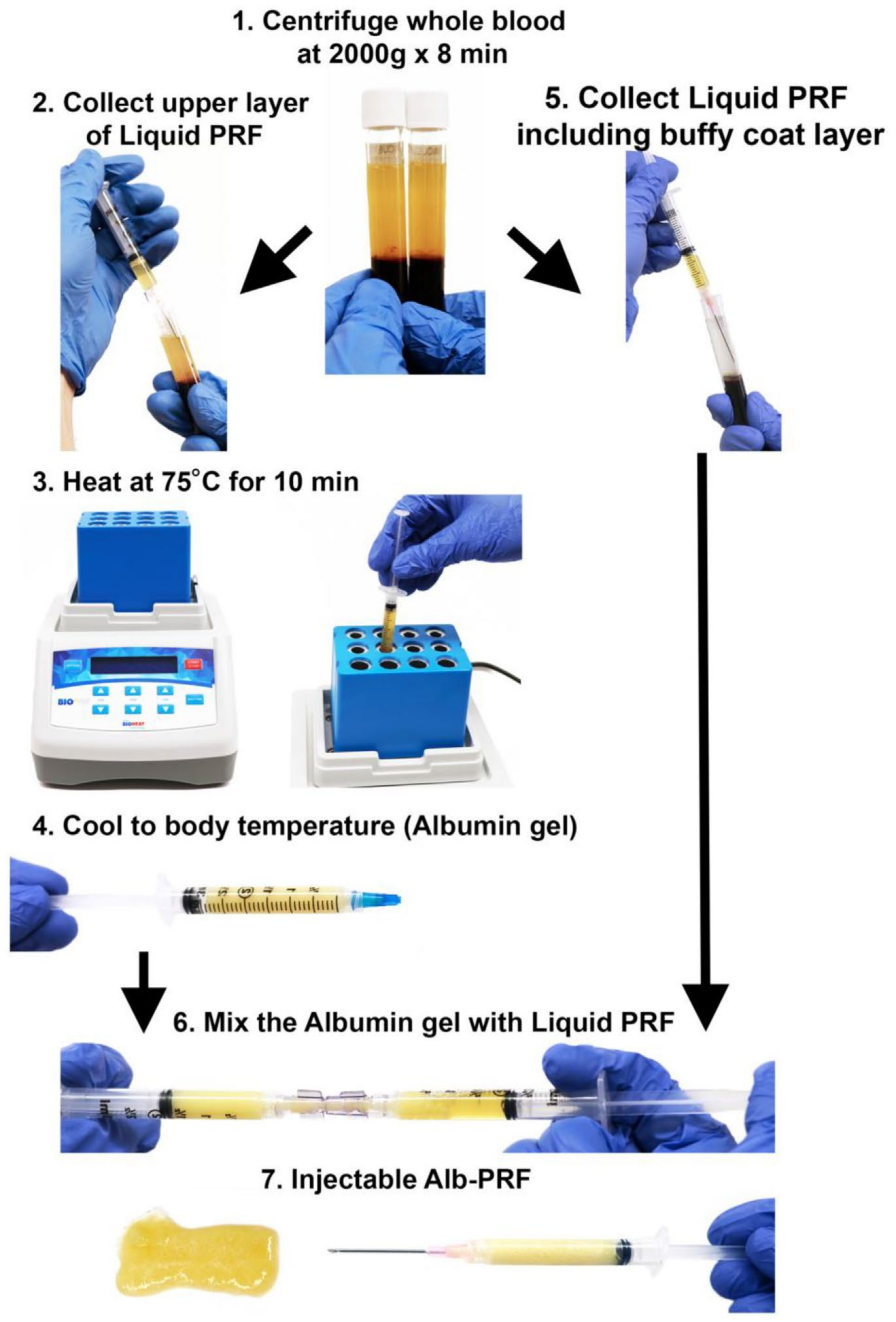
e‐PRF or Alb‐PRF preparation protocol. (1) Whole blood was centrifuged at 2000 *g* for 8 min. The upper layer (yellow layer) shows the liquid plasma layer. (2) The uppermost layer of platelet‐poor plasma (PPP) was collected in a syringe. (3) The collected PPP was heated in a heat block device at 75°C for 10 min and thereafter (4) cooled to room temperature for approximately 10 min. An injectable albumin gel was then prepared. (5) The liquid platelet‐rich layer (liquid‐PRF), including the buffy coat layer with accumulated platelets and leukocytes, was collected in a separate syringe. (6) The albumin gel and native liquid PRF were then thoroughly mixed by utilizing a female–female luer lock connector. (7) Injectable e‐PRF in final ready form. Reprinted with permission from Fujioka‐Kobayashi et al.^56^

Another breakthrough development in the space of PRF therapy has been the novel method developed to enhance the structural properties of the PRF matrix, extending its stability for 4–6 months.^22^ This approach involved heating the uppermost layer of PPP for 10 min after centrifugation, denaturing the serum albumin to enhance the resorption properties of the albumin gel and significantly extending its stability. However, this denaturation process inactivates collected growth factors and causes cell apoptosis due to high temperatures.^22^ In this regard, three different studies by Kargarpour et al. have also shown that the biological properties of PPP prepared using horizontal centrifugation are heat‐sensitive, causing this layer to lose its biological activity after heating.^61^, ^62^, ^79^ To address this limitation, a new protocol was designed to reintroduce viable cells and growth factors back into the albumin gel. The buffy coat or C‐PRF layer, rich in cells and growth factors, is collected after centrifugation and mixed following the cooling of the albumin gel (Figure 21).^22^, ^56^ This enhanced product is referred to as extended PRF (e‐PRF) commercially or Alb‐PRF scientifically.^22^, ^56^ Given that horizontal centrifugation has demonstrated superior separation and concentration of platelets and leukocytes compared to fixed‐angle centrifugation,^36^, ^40^, ^43^, ^46^ and the current evidence supports the efficacy of Alb‐PRF prepared using this method,^56^, ^57^, ^59^ it is the preferred approach for producing Alb‐PRF for clinical use.

In recent years, numerous in vitro, in vivo, and clinical studies on solid and liquid H‐PRF matrices have validated their effectiveness across various regenerative applications, including bone, periodontal, cartilage, skin, and hair regeneration, corneal repair, wound healing, and soft tissue regeneration, as extensively discussed throughout this comprehensive systematic review. Nevertheless, only 13 of the studies compared H‐PRF directly to PRF produced via fixed‐angle centrifugation, and additional studies are further needed, especially human randomized clinical trials.

## 5 CONCLUSION

This comprehensive review was the first of its kind investigating all studies related to horizontal centrifugation of PRF, with special attention placed on its comparison to fixed‐angle centrifugation. This included all comparative and non‐comparative data on cell density/concentrations, fibrin matrix structure, growth factor and cytokine release, cellular interaction, antibacterial and anti‐inflammatory properties, bone, periodontal, cartilage, skin, and hair regeneration, regenerative endodontics, corneal repair, wound healing, and soft tissue regeneration, amongst others. Of the 13 studies comparing horizontal to fixed‐angle centrifugation, 84.6% (11 studies) favored horizontal centrifugation, 15.4% (2 studies) reported no difference, and none favored fixed‐angle centrifugation over horizontal centrifugation. Further clinical studies are indeed needed to validate the superior advantages of H‐PRF over a wide array of clinical applications/indications.

## 6 CLINICAL RELEVANCE

The clinical relevance of this review lies in the demonstrated advantages of horizontal centrifugation to produce PRF. While the current evidence remains limited and further clinical trials are necessary, several studies have now demonstrated that the use of horizontal centrifugation, compared to fixed‐angle centrifugation, can result in higher cell concentrations, more uniform cell distribution, and increased growth factor release. These advantages suggest that the use of H‐PRF may lead to enhanced clinical outcomes when the application of PRF is indicated in various regenerative procedures. Since horizontal centrifugation can also lead to better cell separation, it should also be the preferred method for producing C‐PRF, which allows for higher concentrations of platelets and leukocytes, and Alb‐PRF, an extended variation of PRF that combines albumin gel.

## AUTHOR CONTRIBUTIONS

All authors made substantial contributions to the conception and design of the manuscript. NF and KAAA performed the literature search. All authors drafted the work and revised it critically for important intellectual content, agreed to be accountable for all aspects of the study design and its content, and approved the final submitted version.

## CONFLICT OF INTEREST STATEMENT

Richard J. Miron is the founder of Miron Research and Development in Dentistry LLC that holds intellectual property on the production of PRF. All other authors declare that they have no competing interest.

## ETHICAL APPROVAL

No ethics approval was required for this study since it was a systematic review.

## INFORMED CONSENT

No informed consent was required.

## Data Availability

Data sharing is not applicable to this article as no new data was created or analyzed in this study.
